# Bioactive metabolites and botanical preparations from plants used as medicines and foods in ulcerative colitis: a mechanism-oriented narrative review of multi-target regulation

**DOI:** 10.3389/fphar.2026.1915609

**Published:** 2026-10-30

**Authors:** Jiaxue Chen, Jihong He, Ziwen Xu, Xiaohan Yi, Zixuan Xiao, Guoren Chen, Peiyu Xiong, Yu Lai, Ping Ma

**Affiliations:** School of Basic Medicine, Chengdu University of Traditional Chinese Medicine, Chengdu, China

**Keywords:** intestinal barrier, multi-target regulation, plant-derived metabolites, plants used as medicines and foods, traditional Chinese medicine, ulcerative colitis

## Abstract

Ulcerative colitis (UC) is a chronic relapsing inflammatory bowel disease with a complex pathophysiology involving immune dysregulation, gut microbiota dysbiosis, intestinal barrier impairment, oxidative stress, and regulated cell death. Current therapeutic strategies, including 5-aminosalicylic acid, corticosteroids, immunosuppressants, biologics, and small-molecule agents, have improved disease management; however, incomplete or lost response, adverse effects, and the need for long-term treatment remain important clinical challenges. Bioactive metabolites and botanical preparations from medicinal and food plants have gained increasing attention because of their diverse pharmacological activities and potential to modulate multiple pathological processes involved in UC. This mechanism-oriented narrative review critically summarizes recent evidence on the effects of these plant-derived metabolites, botanical preparations, and combination strategies in UC using a framework that links major UC pathological modules to molecular targets/pathways and functional outcomes, with particular emphasis on anti-inflammatory and antioxidant actions, regulation of ferroptosis and pyroptosis, modulation of autophagy and endoplasmic reticulum stress, protection of the intestinal barrier, and regulation of gut microbiota. The reported mechanisms recurrently converge on signaling networks involving NF-κB, MAPK, Nrf2/ARE, and PI3K/Akt. Evidence from isolated-metabolite cellular studies, extract- and preparation-level studies, animal models, and available human studies varies by the study type. We interpret it according to its mechanistic and translational relevance. Although the available literature indicates that these metabolites may regulate interconnected UC-related pathways through multi-target actions, most mechanistic evidence remains preclinical. Important limitations include incomplete reporting of dose ranges, controls, sample sizes, and quantitative effect estimates; inconsistent documentation of taxonomic authentication and extract characterization; limited metabolite-specific pharmacokinetic and bioavailability data; insufficient translational validation of advanced delivery strategies; variability in microbiota-related findings among experimental models; and limited clinical validation. Further well-designed pharmacological and clinical studies are required to clarify the most robust mechanisms and identify plant-derived metabolites with the greatest potential for further development in UC.

## 1 Introduction

Ulcerative Colitis (UC) is a lifelong inflammatory disease that affects the rectum and colon to varying degrees. In 2023, the prevalence of UC was estimated at 5 million cases worldwide, and incidence is increasing (Le Berre et al., 2023). UC can affect individuals at any age, but it is most commonly diagnosed in people aged 20–40 years. In North America, approximately 1.5 million people currently have a UC diagnosis (Voelker, 2024; Krugliak Cleveland et al., 2022). The pathological features of UC include active erosion of the intestinal mucosa, ulceration, proliferation of granulation tissue, and crypt abscesses. During remission, the features include mucosal atrophy, aberrant crypt architecture, a decreased number of glands, and Paneth cell metaplasia (Liu M. et al., 2024). Common clinical manifestations include abdominal pain, diarrhea, rectal bleeding, weight loss, and fatigue, and long-standing disease is associated with an increased risk of colorectal cancer (Voelker, 2024; Gros and Kaplan, 2023). Diagnosis is based on an integrated assessment of clinical manifestations, inflammatory markers, endoscopic findings, and histological features (Le Berre et al., 2023; Ogata et al., 2025). Current therapeutic strategies include 5-aminosalicylic acid preparations, corticosteroids, immunosuppressants, biologic agents, small-molecule drugs, and surgery for selected severe or refractory cases. Although these therapies have substantially improved disease management, incomplete or lost response, adverse effects, treatment resistance, and the need for long-term therapy remain important clinical challenges (Le Berre et al., 2023; Liu et al., 2022).

Traditional Chinese medicine (TCM) has an extensive history of addressing UC. TCM may successfully mitigate symptoms such as stomach discomfort, diarrhea, and rectal bleeding in people with UC. Previous clinical and experimental studies have investigated formulations and botanical drugs such as Shenling Baizhu powder (SBP), Qingchang Huashi formula (QCHS), and *Sophora flavescens* Aiton in UC (Chen J. et al., 2022; Shen et al., 2021; Chen et al., 2020). These findings provide a basis for further pharmacological investigation. However, they should not be interpreted as evidence that the relevant plant species or metabolites are unique to TCM or that all such preparations have established efficacy and safety. The traditional Chinese concept commonly translated as “medicine and food share the same origin” broadly refers to biological resources used in both dietary and medicinal contexts (Panossian and Lemerond, 2025). Examples described in this context include *Lycium barbarum* L., *Crataegus pinnatifida* Bunge, and *Ziziphus jujuba* Mill. In this review, we refer to these materials as plants used as medicines and foods. Because some of these plants are also consumed as foods, their derived metabolites and preparations have drawn interest for potential long-term applications; however, their safety should be evaluated based on pharmacological and clinical evidence rather than inferred from food use alone. Given the complex, multifactorial pathophysiology of UC, plant-derived metabolites and botanical preparations may influence multiple interconnected pathways and targets, supporting investigation of their potential role in multi-target therapeutic strategies. Accordingly, this article is positioned as a mechanism-oriented narrative review rather than a catalog of individual metabolites or botanical preparations. The review organizes the available evidence around major UC pathological modules, including immune and inflammatory dysregulation, oxidative stress, intestinal barrier impairment, regulated cell death, autophagy and endoplasmic reticulum stress (ERS), and microbiota–host interactions, while considering genetic, dietary, and environmental factors as important modifiers of disease. Within this framework, mechanistic evidence is interpreted in a common sequence: from pathological modules to molecular targets/signaling pathways and then to functional outcomes or phenotypes. The review further considers botanical preparations, extracts, isolated plant-derived metabolites, and combination strategies, while distinguishing evidence obtained from cellular studies, animal models, and available human studies. Particular attention is given to the extent to which the reported findings support mechanistic interpretation and potential clinical translation, along with the limitations related to study design, pharmacological exposure (dose or concentration and duration), use of appropriate controls, model relevance, sample size and quantitative outcomes, pharmacokinetics, bioavailability, delivery strategies, taxonomic authentication, extract characterization, and reproducibility of microbiota-related findings.

To support this narrative synthesis, we conducted a targeted literature search in PubMed, Web of Science, and Google Scholar for publications mainly from 2021 to 2025, including key earlier background references when necessary. The search combined UC-related terms with terms describing botanical preparations, extracts, and plant-derived metabolites, together with mechanism-related terms. A representative search structure was: (“ulcerative colitis” OR “UC”) AND (“traditional Chinese medicine” OR “medicinal and edible” OR “medicine-food homology” OR “plants used as medicines and foods” OR “botanical drug*” OR “botanical preparation*” OR extract* OR metabolite* OR monomer*) AND (inflamm* OR “oxidative stress” OR “intestinal barrier” OR microbiota OR ferroptosis OR pyroptosis OR autophagy OR “endoplasmic reticulum stress”). We adapted database-specific syntax and term combinations where necessary and used simplified combinations of these terms in Google Scholar. Legacy terms including “traditional Chinese medicine,” “medicinal and edible,” “medicine–food homology,” and “monomer*” were retained only as search terms to capture earlier literature and are not used as preferred terminology in this review. We considered studies relevant when they investigated botanical preparations, extracts, plant-derived metabolites, or combinations in UC-related cellular, animal, or human settings and reported pharmacological, mechanistic, barrier-related, immunological, oxidative, cell-death-related, or microbiota-related outcomes. Mechanistic interpretation focused mainly on primary experimental studies, whereas existing reviews and meta-analyses were used mainly for background and clinical context rather than as primary mechanistic evidence. We treated *in silico* and network-pharmacology findings as hypothesis-generating evidence rather than equivalent experimental evidence. We did not include duplicate or clearly irrelevant reports in the narrative synthesis. Where available, dose or concentration, experimental model, controls, treatment duration, sample size, quantitative outcomes, characterization of materials and extracts, taxonomic information, pharmacokinetics, bioavailability, delivery strategies, and translational limitations were examined. The search was targeted and non-exhaustive and was intended to support a narrative synthesis; no formal systematic-review or meta-analytic protocol was applied.

## 2 The pathogenic factors of UC

The exact pathogenesis of UC remains unclear; however, its development is widely linked to immune dysregulation, imbalances in the intestinal microbiota, disruption of the intestinal barrier, genetic predispositions, dietary influences, and environmental factors. These interrelated factors shape the intestinal microenvironment and influence disease susceptibility and progression. In this review, we consider them major pathogenic factors and disease modifiers, providing context for the core molecular and cellular mechanisms discussed in Section 2 and for interpreting the actions of plant-derived metabolites and botanical preparations in subsequent sections.

### 2.1 Immune imbalance

Dysregulation of T-cell subsets, specifically over-activation of Th17 cells and reduced activity of regulatory T-cells (Tregs), is a key feature of the complex pathophysiology of UC. By recruiting neutrophils to the intestinal epithelium and amplifying inflammatory signaling, Th17 cells, a major source of pro-inflammatory cytokines such as IL-17 and IL-22, contribute to chronic intestinal inflammation. In contrast, Tregs suppress excessive immune responses by secreting IL-10 and TGF-β, and reduced Treg activity may contribute to impaired immune tolerance and sustained inflammation in patients with UC. Experimental evidence indicates that modulation of the Th17/Treg balance can reduce inflammation and promote mucosal healing. In a mouse model, for example, Astragalus polysaccharide (APS) was shown to modulate the TIGIT/CD155 signaling axis, decreasing Th17 activity and increasing Treg responses, thereby significantly alleviating colitis (Wan J. et al., 2024). These findings support the importance of the Th17/Treg balance as a potential intervention node in UC-related immune dysregulation. However, the evidence is predominantly preclinical, and its relevance to human UC requires further validation. Excessive secretion of pro-inflammatory cytokines, such as TNF-α, IL-6, and IL-1β, is another important feature of immune dysregulation in UC. These cytokines promote immune cell infiltration into the intestinal mucosa and can disrupt epithelial barrier integrity, thereby sustaining inflammatory responses. TNF-α can impair tight junction (TJ) integrity, whereas IL-6 and IL-1β further amplify inflammatory signaling and may influence the Th17/Treg balance. Experimental approaches targeting this cytokine network have shown beneficial effects in preclinical models. For example, a reactive oxygen species (ROS)-responsive nanotherapeutic strategy reduced the inflammatory cytokine levels and improved mucosal barrier integrity in a mouse model of colitis (Yan H. et al., 2023). These findings support the close interaction among cytokine signaling, oxidative stress, and barrier dysfunction. However, most evidence comes from experimental models, and its translational relevance requires further evaluation.

Abnormal plasma-cell responses may also contribute to persistent mucosal inflammation in UC. Plasma cells in the mesenteric lymph nodes and intestinal lamina propria have been reported to show increased activation and altered distribution during intestinal inflammation. Increased IgG-secreting plasma-cell responses have been associated with disease activity. At the same time, a type-1 inflammatory microenvironment characterized by IFN-γ and CXCL9/CXCL10 may further promote B-cell and plasma-cell activation (Uzzan et al., 2022). These findings indicate that abnormal humoral immune responses may participate in the amplification of intestinal inflammation.

Nevertheless, the relative contribution of plasma cells to UC pathogenesis and their importance across disease stages remain unclear. An imbalance between the tissue-repairing M2 and pro-inflammatory M1 phenotypes exacerbates chronic inflammation, underscoring macrophage dysfunction as another critical factor in immune dysregulation in UC (Qu et al., 2025). High levels of IL-12, IL-23, and TNF-α secreted by M1 macrophages in UC promote neutrophil recruitment and long-term tissue damage. Conversely, M2 macrophages promote tissue repair by secreting IL-10 and TGF-β. In a murine colitis model, an M2-mimicking Janus nanomotor microsphere delivery system was reported to modulate macrophage polarization, reduce inflammatory burden, and improve mucosal healing (Luo et al., 2024). This study supports the relevance of macrophage polarization as a potential intervention node, although the evidence remains preclinical. The pathogenic immune dysregulation in UC arises from the complex interaction of dysregulated T-cell subsets, excessive cytokine production, abnormal plasma-cell activity, and altered macrophage polarization. The interconnected nature of these processes indicates that modulating a single immune pathway may be insufficient to address the complexity of mucosal inflammation fully. Accordingly, these immune components should be considered as interacting intervention nodes within the broader pathological network of UC. However, most mechanistic evidence remains preclinical, and further human studies are required to determine their translational and therapeutic relevance.

### 2.2 Gut microbiota dysbiosis

Gut microbiota dysbiosis is closely associated with UC pathogenesis and can influence microbial composition, intestinal immune homeostasis, and epithelial barrier function. UC has been linked to alterations in potentially beneficial commensal bacteria, including *Lactobacillus* and Bifidobacterium, along with increases in opportunistic microorganisms such as *Escherichia coli* and members of Proteobacteria. Such changes may disturb interactions between the gut microbiota and intestinal epithelial cells, impair mucosal immune tolerance, and contribute to inflammatory responses. Microbiota-derived metabolites also regulate immunity. For example, 12-ketolithocholic acid was reported to inhibit group-3 innate lymphoid cell (ILC3)-mediated IL-17A production, indicating a potential link between microbial metabolism and intestinal immune responses (Li N. et al., 2023).

Beyond changes in microbial composition, dysbiosis can alter microbial metabolic functions important for intestinal homeostasis. Short-chain fatty acids (SCFAs), particularly butyrate, contribute to epithelial barrier integrity, immune regulation, and goblet-cell function. Reduced SCFA production may, therefore, contribute to impaired tight junction integrity and altered inflammatory signaling. In DSS-induced colitis, QCHS was reported to restore SCFA production and promote goblet-cell repair of TJs (Hu J. et al., 2021). *Atractylodes macrocephala* Koidz. volatile oil was also reported to alleviate experimental colitis by modulating gut microbiota composition and microbial metabolism. However, its effects were not primarily attributed to restoration of SCFAs or goblet-cell function (Cheng H. et al., 2023). These findings underscore the role of gut microbiota metabolism in UC pathogenesis and support investigating microbiota-targeted therapies designed to restore metabolic balance. Beyond these specific examples, botanical preparations have also been reported to influence microbial diversity, inflammatory responses, and metabolic homeostasis (Wang Y. et al., 2024), further supporting the gut microbiota as an important component of the pathological network of UC. However, microbiota findings should be interpreted cautiously because microbial composition can vary with diet, housing conditions, animal strain, sex, and experimental model. Consequently, changes in individual bacterial taxa observed in a single study may not be generalizable across experimental systems or to patients with UC. In this review, we, therefore, place greater emphasis on microbiota changes that recur across studies or are supported by corresponding functional outcomes, such as alterations in microbial metabolites, inflammatory responses, or intestinal barrier function, whereas isolated taxonomic changes are interpreted more cautiously. Further studies under comparable experimental conditions, with appropriate clinical validation, are needed to determine which microbiota-related effects are reproducible and translationally relevant.

### 2.3 Intestinal barrier impairment

The intestinal epithelial barrier is essential for maintaining intestinal homeostasis by separating luminal microorganisms and antigens from the mucosal immune system. In UC, intestinal epithelial cells (IECs) face persistent inflammatory and oxidative stress, which can impair epithelial integrity and increase mucosal permeability. IEC damage may also amplify immune responses. For example, mitochondrial DNA (mtDNA) released from injured epithelial cells can bind mitochondrial transcription factor A (TFAM) and be taken up by dendritic cells, thereby activating the STING/IRF3/NF-κB pathway and increasing the production of IL-12 family cytokines. These signals can further promote Th1 and Th17 responses and aggravate intestinal inflammation (Cai et al., 2024). This mechanism illustrates the reciprocal relationship between epithelial injury and immune activation in UC. However, this mechanistic evidence is primarily preclinical, and its relevance to human UC remains to be established.

Disruption of tight junction proteins represents another important feature of barrier impairment. Proteins such as occludin and claudin-1 regulate paracellular permeability, and altered expression or distribution of these proteins can facilitate microbial translocation and exposure to inflammatory mediators. Mechanotransduction mediated by Piezo1 and downstream ROCK signaling has been implicated in regulating claudin-1 and epithelial barrier function (Jiang et al., 2021). In a DSS-induced colitis model, mangiferin was reported to increase tight junction protein expression, improve epithelial barrier integrity, and modulate macrophage polarization (Xiao et al., 2024a). These findings support the close interaction between epithelial barrier regulation and mucosal immune responses. However, most evidence comes from animal models, and validation in patients with UC remains limited. The mucus layer provides an additional protective barrier between the intestinal microbiota and the epithelial surface. MUC2, the major secreted mucin produced by colonic goblet cells, is essential for maintaining this separation. Altered MUC2 production or mucus organization may expose IECs to luminal microorganisms and inflammatory stimuli, thereby contributing to mucosal injury. Strategies involving dietary intervention, fecal microbiota transplantation, and experimental mucus repair have been investigated for their effects on the MUC2 barrier and its interaction with the gut microbiota (Yao et al., 2021). However, these approaches differ substantially in mechanism and the level of evidence, and their effects on mucus restoration should, therefore, be interpreted separately rather than considered equivalent. Overall, intestinal barrier impairment in UC involves epithelial injury, tight junction disruption, and mucus layer dysfunction. These abnormalities can increase microbial exposure and immune activation, while inflammatory and oxidative stress can further aggravate barrier damage. Thus, barrier dysfunction should be viewed as part of an interconnected pathological process rather than as an isolated consequence of UC. Restoration of barrier integrity is, therefore, an important functional outcome when evaluating potential interventions, although changes in individual barrier markers should be interpreted together with histological, inflammatory, and functional evidence.

### 2.4 Genetic factors

Genetic susceptibility contributes to the development and heterogeneity of UC through interactions with immune regulation, epithelial barrier function, and environmental exposures. Genome-wide association studies have identified multiple susceptibility loci associated with UC, supporting the involvement of pathways related to host immunity and epithelial homeostasis. Variants involving CXCR2 and genes within the MHC class-III region have been associated with altered inflammatory and immune responses (Chen B. et al., 2024). Environmental exposures may further influence these pathways through epigenetic regulation, indicating that genetic susceptibility and environmental factors interact to shape disease risk. However, the functional consequences of many risk loci remain incompletely understood, and translating genetic findings into mechanistically informed therapies remains challenging. Beyond loci related to epithelial and innate immune regulation, genetic variation affecting cytokine pathways may also contribute to UC susceptibility and inflammatory responses. Variants involving genes such as IL-10 and TNFSF15 have been associated with altered immune regulation and persistent intestinal inflammation (Sun et al., 2024). However, these associations do not indicate that individual genetic variants independently determine disease development. Rather, genetic factors are likely to modify susceptibility and disease phenotype in combination with immune, microbial, dietary, and environmental influences.

### 2.5 Dietary, environmental, and psychological factors

Dietary patterns may influence intestinal inflammation, gut microbiota composition, and metabolic function in UC. Diets high in refined carbohydrates, saturated fats, and other inflammatory components have been associated with increased inflammatory markers, including C-reactive protein and IL-6 (Yin S. et al., 2025). In contrast, low-fat, high-fiber dietary patterns have been associated with reduced inflammatory activity and changes in gut microbiota diversity, particularly in patients with UC in remission (Fritsch et al., 2021). These findings have encouraged the development of individualized nutritional strategies as adjunctive approaches in UC management (Herrador-López et al., 2023). However, dietary effects may vary according to disease activity, baseline nutritional status, gut microbiota composition, and concurrent treatment, and associations observed in individual studies should not be interpreted as evidence of a uniform therapeutic effect. Dietary fiber may be particularly relevant because microbial fermentation of certain fibers contributes to SCFA production, including butyrate, which can influence epithelial barrier integrity and inflammatory responses. A study of patients with UC found that higher soluble dietary fiber intake was associated with lower disease activity and increased SCFA production (Martínez-Vázquez et al., 2024). Experimental studies have also shown that different fiber types can alter gut microbiota composition, SCFA levels, and inflammatory cytokines such as TNF-α and IL-6 (Cao et al., 2024). Nevertheless, differences in fiber type, dose, intervention duration, baseline diet, and experimental model may influence these outcomes and limit direct comparison across studies. Environmental exposures may also modify UC susceptibility or disease activity through inflammatory and epigenetic mechanisms. Smoking has been associated with changes in DNA methylation at loci including DNMT3A and AHRR, which may influence immune and inflammatory regulation (Zhang H. et al., 2024). Exposure to fine particulate matter (PM2.5) has also been associated with increased outpatient visits for UC and may interact with inflammatory or epigenetic pathways (Duan et al., 2021). However, these observations are primarily associative and do not by themselves establish a direct causal relationship between a specific environmental exposure and the onset or progression of UC. Psychological factors, including anxiety and depression, may interact with intestinal inflammation through the gut–brain axis and changes in the gut microbiota. Transfer of fecal microbiota from patients with UC to mice was associated with reduced colonic neuropeptide Y (NPY) expression, accompanied by increased anxiety- and depression-like behaviors (Joo et al., 2024). Patients with UC and psychological comorbidities have also shown alterations in gut microbial composition and metabolomic profiles, including changes in tryptophan metabolism (Yuan et al., 2021). These findings support potential bidirectional interactions among the psychological status, microbial metabolism, and intestinal inflammation. However, the available evidence remains heterogeneous, and microbiota alterations should not be interpreted as direct causes of psychological symptoms without further mechanistic and clinical validation.

## 3 The pathogenesis of UC

The pathological modules discussed above converge on several interconnected mechanistic processes, including inflammatory signaling, oxidative stress, ferroptosis, pyroptosis, autophagy, and ERS. These processes are not independent; rather, they interact with and reinforce one another to sustain mucosal injury in UC. This section outlines the key mechanistic processes that provide the molecular context for interpreting the effects of plant-derived metabolites and botanical preparations discussed later. Most evidence summarized here is derived from preclinical models, and human validation remains limited.

### 3.1 Inflammatory response

Abnormal inflammatory signaling is a central mechanism contributing to persistent mucosal inflammation and epithelial injury in UC. Activation of TLR4 signaling in colonic epithelium can stimulate the NF-κB pathway and promote the production of inflammatory cytokines, including TNF-α and IL-6. These inflammatory mediators contribute to immune cell recruitment, epithelial barrier impairment, and amplification of intestinal inflammation. Taurine supplementation has been reported to reduce inflammatory and oxidative responses, decrease TLR4/NF-κB signaling, and improve intestinal barrier function by increasing tight junction protein expression and goblet cell density in experimental colitis models (Zheng et al., 2024). The opposing roles of cytokines such as IL-1β and IL-10 highlight the dynamic balance between inflammatory activation and immune regulation in UC (Nakase et al., 2022). These findings indicate that inflammatory signaling represents an important intervention node within the broader pathological network of UC. However, most available evidence is derived from animal models, and the extent to which these mechanisms translate to human UC remains to be established.

### 3.2 Oxidative stress

Oxidative stress is an important component of UC pathophysiology because it promotes inflammatory amplification, mitochondrial dysfunction, and impairment of mucosal integrity.

Excessive production of ROS, primarily driven by immune activation, induces lipid peroxidation, protein modification, and DNA damage, thereby contributing to epithelial injury. Elevated malondialdehyde (MDA) levels, a marker of lipid peroxidation, are associated with disease severity and inflammatory activity and may distinguish active and remission phases in UC (Tratenšek et al., 2024). Mitochondrial dysfunction and altered regulatory factors such as CPT1A may further aggravate oxidative stress and contribute to epithelial barrier impairment (Wu Y. et al., 2025). Experimental modulation of oxidative stress, such as treatment with CaGA nanozymes, has been reported to reduce ROS accumulation, restore tight junction integrity, and improve colitis-related pathological changes in TJs (Lin et al., 2025). These findings highlight the interaction among oxidative stress, inflammation, and intestinal barrier dysfunction in UC. However, most evidence remains preclinical, and standardized biomarkers, pharmacokinetic characteristics, and clinical validation are still limited.

### 3.3 Ferroptosis

Ferroptosis is an iron-dependent form of regulated cell death characterized by iron accumulation, lipid peroxidation, and impaired antioxidant defense, and it has been increasingly implicated in UC pathophysiology. Excessive intracellular iron accumulation and reduced antioxidant capacity can enhance oxidative damage, promote epithelial-cell injury, and compromise mucosal-barrier integrity. Bioinformatics analysis of UC-related genes identified ferroptosis-associated immune genes, including DUOX2, indicating a potential link between ferroptosis and immune dysregulation (Cai et al., 2025). Experimental studies further showed that inhibition of GPX4, a key regulator of ferroptosis, contributes to epithelial injury, whereas HIF-1α-mediated restoration of GPX4 activity reduced inflammation and colonic epithelial-cell death in experimental colitis models (Hu et al., 2025). In addition, immune infiltration analyses have linked ferroptosis-related changes to increased recruitment of neutrophils and macrophages, which may further amplify inflammatory responses (Dong et al., 2024). These findings indicate that ferroptosis represents an important mechanistic link among oxidative stress, epithelial injury, and immune activation. However, most evidence remains preclinical, and further studies are needed to clarify ferroptosis’ specific contribution to human UC and evaluate clinically relevant ferroptosis-related biomarkers and therapeutic strategies.

### 3.4 Pyroptosis

Pyroptosis is an inflammatory form of programmed cell death mediated by gasdermin family proteins and is increasingly recognized as a mechanism involved in UC-associated epithelial injury and inflammatory amplification. Transcriptome analyses combined with experimental validation have identified increased expression of pyroptosis-related genes, including TLR4 and CASP11, in UC and active disease, indicating the involvement of pyroptosis-related pathways in intestinal inflammation (Zhao et al., 2024). In experimental models of colitis, pharmacological inhibition of the TXNIP–NLRP3–GSDMD axis by phenethyl isothiocyanate (PEITC) reduced IL-1β release and attenuated colonic epithelial damage (Wang J. et al., 2025). In addition, natural compounds such as quercetin have been reported to modulate pyroptosis-related signaling by regulating key nodes, including caspase-1 and NF-κB (Zhao et al., 2024; Lu et al., 2025). These findings indicate that pyroptosis may link inflammatory signaling to epithelial injury. However, current evidence is mainly derived from transcriptomic analyses and experimental models, and further studies are needed to clarify the clinical relevance of pyroptosis regulation and its potential as a therapeutic target in human UC.

### 3.5 Autophagy

Autophagy is an essential cellular process that removes damaged organelles and proteins and plays an important role in maintaining intestinal epithelial integrity and immune homeostasis in UC. Impaired autophagic activity may contribute to epithelial-barrier disruption, increased inflammatory responses, and loss of intestinal homeostasis. Conversely, appropriate modulation of autophagy-related pathways may promote mucosal repair and cellular protection. Evidence indicates that autophagy induction through mechanisms such as estrogen receptor β-mediated regulation of branched-chain amino acid transport can promote epithelial-cell turnover and mucosal healing in experimental models (Guo et al., 2025). In addition, autophagy-mediated mitochondrial regulation was reported to alleviate colonic inflammation by restoring enterocyte energy homeostasis and reducing oxidative stress, as observed after treatment with Tou Nong San (TNS) (Li Z. et al., 2025). Natural compounds such as SBP have also been reported to reinforce intestinal barrier function through tight junction stabilization and autophagy modulation (Chen J. et al., 2022). These findings indicate that autophagy represents an important regulatory node linking epithelial homeostasis, mitochondrial function, and inflammation. However, current evidence remains predominantly preclinical, and the context-dependent effects of autophagy modulation require further clarification before clinical translation.

### 3.6 Endoplasmic reticulum stress

ERS is an important cellular stress response involved in UC pathophysiology because it affects intestinal epithelial homeostasis and can intensify inflammatory responses. Accumulation of misfolded proteins in the endoplasmic reticulum activates the unfolded protein response (UPR); however, persistent ERS may promote apoptosis and disrupt epithelial-barrier integrity. Increased expression of glucose-regulated protein 78 (GRP78), a key UPR regulator, has been associated with increased intestinal inflammation and disease severity. In contrast, intestinal epithelial cell-specific ubiquitin-specific peptidase 13 (USP13) has been reported to reduce colonic inflammation by suppressing GRP78-mediated signaling (Qian et al., 2025). Bioinformatics analysis identified ERS-related genes associated with immune-cell infiltration and revealed heterogeneity among ERS-related UC subtypes, indicating potential interactions between ERS and immune regulation; however, these findings require experimental validation (Deng et al., 2023). In murine models, pharmacological interventions such as artesunate have been reported to alleviate experimental colitis by modulating immune responses and reducing ERS (Yin X. et al., 2025). These findings highlight the interaction among ERS, inflammation, immune regulation, and epithelial function in UC. Nevertheless, most evidence remains preclinical, and the clinical relevance of ERS-targeting strategies requires further investigation. Figure 1 places these modules within the immune-microbiota-barrier context.

**FIGURE 1:**
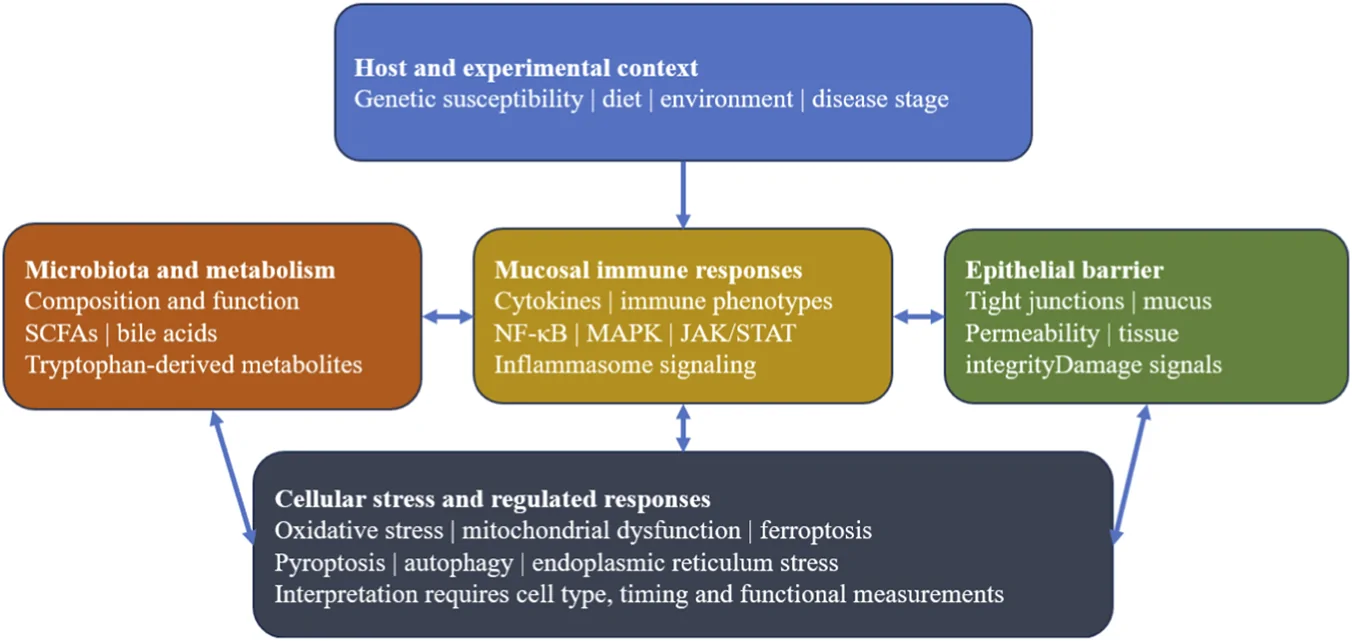
UC pathological modules and their interactions.

## 4 Botanical preparations from plants used as medicines and foods and their multi-target regulation of UC

Researchers have investigated botanical formulations for their effects on several interconnected features of UC, particularly inflammatory injury, epithelial repair, and microbial metabolism. Huanglian-Ganjiang Tang alleviated experimental colitis by increasing vitamin D receptor expression and reducing necroptosis-associated signaling (Xiong et al., 2022). Studies of Lizhong decoction extended this focus to ferroptosis and the gut microbiota: one linked epithelial protection to Nrf2/SLC7A11/GPX4-associated responses (Li F. et al., 2024). At the same time, another found that protection was lost after antibiotic treatment and could be transferred through fecal transplantation (Huang H. et al., 2025). These findings indicate that both epithelial stress responses and microbial activity may contribute to its effects. Ganjiang-Huangqin-Huanglian-Renshen decoction also reduced inflammatory and oxidative injury, accompanied by changes in mitophagy-associated proteins (Zhou C. et al., 2024). Some formulation studies have begun to connect these experimental observations with clinical outcomes. In a prospective cohort, adding Shenling Baizhu San to mesalamine for 8 weeks was associated with a greater decrease in the Ulcerative Colitis Endoscopic Index of Severity than that with mesalamine alone (5.13 *versus* 3.50 points, P < 0.01), together with changes in microbial tryptophan metabolites (Jiao et al., 2022). The non-randomized design limits conclusions about the added benefit, but the study provides a clinical context for further investigation of host–microbial interactions. Other formulations have been examined more directly for their effects on inflammatory responses. Sijunzi decoction reduced NET-associated signals and IL1B/TNF expression in experimental models (Zhang D. et al., 2023), whereas Renshen Baidu powder alleviated TNBS-induced colitis alongside reduced PI3K/Akt/NF-κB phosphorylation (Ye X. et al., 2022). Gegen Qinlian decoction illustrates how inflammatory control and barrier repair may be linked through microbial and host pathways. Hu et al. observed reduced Enterobacteriaceae expansion alongside increased PPAR-γ activity, and attenuation of protection with a PPAR-γ antagonist supported the involvement of this pathway (Hu et al., 2022). A separate study associated improvement with lower IL-6/JAK2/STAT3 signaling and reduced accumulation of both Th17 and Treg cells in lymphoid tissues, indicating a broader adjustment of T-cell responses rather than selective Treg expansion (Zhao et al., 2021). Wang et al. further linked barrier recovery to tryptophan metabolites and AhR/IL-22 signaling, with an AhR antagonist weakening several protective responses (Wang et al., 2023a). Together, these studies support a connection between microbial metabolism, mucosal immunity, and epithelial integrity, although the contribution of each link remains incompletely resolved.

Related findings have been reported for other formulations. Huaihua San reduced inflammatory injury and colonic vascular leakage while altering microbial composition (Liu et al., 2020). Gancao Xiexin decoction likewise improved colitis and microbial metabolic profiles, but its benefit was no longer detectable in antibiotic-treated mice (Luo et al., 2022). Its acetate increase was non-significant, indicating that the metabolic response should not be reduced to a general restoration of short-chain fatty acids. Danggui Buxue decoction improved epithelial and inflammatory outcomes with reduced PI3K/AKT phosphorylation (Liu T. Y. et al., 2025). Da-yuan-yin decoction showed anti-complement activity in a hemolysis assay and reduced complement-, TLR4/NF-κB-, and NET-associated measures in colitic mice (Yang et al., 2024), indicating several possible points of interaction with mucosal inflammation. Epithelial recovery is also evident in studies of Wumei and Shengjiang Xiexin decoctions. Wumei decoction improved barrier function and reduced Th1/Th17-associated responses in both chronic DSS and T-cell-transfer colitis models, with microbial changes observed in the latter (Wu et al., 2022). Shengjiang Xiexin decoction improved epithelial injury in mice and small-intestinal organoid growth, accompanied by increased Wnt/β-catenin-associated regenerative signals (Chen C. et al., 2025), but clinical evidence remains less consistent. A systematic review of Xiangsha Liujunzi Tang reported favorable treatment responses, but it combined trials of monotherapy, add-on treatment, and oral-plus-enema regimens (Yuan et al., 2020). The non-significant add-on subgroup, variable study quality, and sparse adverse-event reporting leave its comparative efficacy and safety uncertain.

Across these studies, reduced inflammation and epithelial recovery recur more consistently than any single molecular mechanism. Antagonist and microbiota-transfer experiments provide stronger mechanistic support than expression changes alone, but the findings remain specific to the preparations and models tested. They cannot establish the contribution of individual constituents or pharmacological synergy. Supplementary Table S1 brings together the treatment conditions, quantitative outcomes, and study limitations needed to interpret these differences.

## 5 Single-resource botanical preparations

Studies of single-resource preparations include the following examples. *Coix lacryma-jobi* L. var. *ma-yuen* (Rom.Caill.) Stapf ameliorates DSS-induced colitis symptoms in mice by modulating T-lymphocyte subsets, restoring innate immune cell populations, reducing inflammatory mediators such as IL-6, and mitigating oxidative stress (Zhou et al., 2021). *Portulaca oleracea* L., rich in polyphenols, increases the abundance of beneficial gut bacteria in UC, thereby indirectly promoting SCFA-related metabolism. Simultaneously, it markedly decreases serum CRP levels and colonic myeloperoxidase (MPO) activity, while inhibiting the aberrant expression of inflammation-related proteins, thereby mitigating inflammation associated with UC (Li Y. et al., 2025). Researchers have determined that *Lonicera japonica* Thunb. ameliorates experimental UC through anti-inflammatory effects, regulation of the gut microbiota, and increased SCFAs, with medium-to-high doses proving more efficacious (Zang et al., 2024). A study examined the effectiveness of *Dendrobium huoshanense* Z.Z.Tang and S.J.Cheng (*D. huoshanense)* in treating UC using network pharmacology and *in vivo* experiments. In DSS-induced UC mice, *D. huoshanense* decreased IL-6/TNF-α levels, elevated IL-10/TGF-β levels, improved colon condition, modulated targets/pathways, and provided preclinical evidence for further evaluation of *D. huoshanense* (Yao et al., 2024). Çimen et al. investigated *Lycium barbarum* L. in a DSS-induced UC model in Wistar rats. The LB + DSS group exhibited elevated p53/Beclin1 levels, reduced TIGAR/MDA levels, increased GSH/CAT levels, and possibly no histopathological evidence of colitis, demonstrating that LB safeguards the colon by modulating apoptosis, autophagy, and anti-oxidative processes (Çimen et al., 2024).

Single-resource preparations have been associated with immune, microbial, and epithelial outcomes in experimental models (Supplementary Table S2). Their relationship to a particular metabolite remains uncertain when the administered material is incompletely characterized. Food-use does not establish safety at experimental doses, and dietary incorporation cannot be inferred from a short-term animal response.

## 6 Metabolites and separately characterized bioactive fractions

### 6.1 Alkaloids

The alkaloid-related literature primarily links inflammatory and immune signaling to epithelial outcomes. Nuciferine, hordenine, magnoflorine, piperlongumine, and other nitrogen-containing metabolites are considered alongside explicitly identified alkaloid-enriched preparations. Ramulus Mori alkaloids are a fraction rather than a single molecule. Supplementary Table S3 records material and model distinctions. Interpretation follows the pathological module, reported intervention node, and functional outcome. Promising approaches, such as mucoadhesive formulations including novel colon-targeted PLGA microspheres modified with sodium alginate and loaded with Ramulus Mori alkaloids, improve therapeutic effects in UC models and target drug delivery to inflammatory sites. Compared with free SZ-A without delivery modulation, SZ-A microspheres showed better results in reducing oxidative stress and DSS-induced colonic inflammation (Wang J. et al., 2025). These findings support a formulation-related benefit, but improved disease outcomes alone do not establish increased bioavailability. Ferroptosis’ newly recognized involvement in enterocyte dysfunction during UC opens new avenues for therapeutic intervention. By activating the Nrf2/GPX4 signaling axis and lowering lipid peroxidation caused by Fe2+ overload, the alkaloid dictamnine has been shown to alleviate DSS-induced colitis (Yuan et al., 2025). Nelumbo nucifera-derived nuciferine effectively reduces inflammation in DSS-induced UC by suppressing the MAPK/NF-κB/NLRP3 pathways, restoring TJ proteins such as ZO-1 and claudin-1 to improve epithelial barrier integrity, and significantly lowering pro-inflammatory cytokines. Furthermore, nuciferine affects gut microbiota composition, which is crucial for maintaining long-term mucosal homeostasis, and regulates immune responses by rebalancing the Th1/Th2 and Treg/Th17 profiles (Zhu et al., 2022; Kulhari et al., 2023). Some studies have investigated hordenine’s role in DSS-induced UC in mice. Hordenine has been shown to reduce DAI, mitigate weight loss and colon shortening, inhibit pro-inflammatory cytokines, protect the intestinal barrier via ZO-1/occludin, and suppress the SPHK1/S1PR1/STAT3 pathway and Rac1 (Xu Y. et al., 2023). Magnoflorine exerts anti-UC effects by suppressing the JAK2/STAT3 signaling pathway, significantly lowering mRNA and protein levels of JAK2, STAT3, and inflammatory markers such as IL-6, IL-16, and TNF-α, while restoring SOCS3 levels (Wen et al., 2024). Although it had little effect on overall colitis symptoms, piperlongumine showed effectiveness in reducing mucosal inflammation and inflammatory factors such as COX-2 and β-catenin (Huang et al., 2020). By altering gut microbiota composition, attenuating oxidative stress markers such as MDA, and upregulating TJ proteins, sanshools, including the tested sanshool preparation, further improved mucosal integrity (Chen J. et al., 2024). Betaine, a natural osmotic agent, showed a notable ability to restore intestinal-barrier integrity and mitigate UC symptoms by inhibiting the NF-κB/MLCK/p-MLC signaling pathway, reducing TNF-α-induced TJ protein disruption, and alleviating systemic inflammation by downregulating IL-6 and TNF-α (Dou et al., 2023; Chen L. et al., 2022). Leonurine has shown potential in treating UC by reducing serum inflammatory factors, downregulating NF-κB, and regulating the gut microbiota (Zheng et al., 2021).

The recurring pattern is concurrent modulation of inflammation and barrier-associated endpoints, but the strength of evidence varies. Changes in cytokines and TJ proteins do not distinguish primary targets from secondary responses to reduced injury. Piperlongumine provides a useful qualification: molecular or mucosal changes were described without a corresponding broad improvement in colitis symptoms (Huang et al., 2020). Delivery-system effects must also be separated from the intrinsic effects of the loaded fraction (Wang M. et al., 2025). These results justify further mechanism testing, not a class-wide claim of safety or clinical efficacy.

### 6.2 Flavonoids

Flavonoid studies span inflammatory signaling, epithelial stress responses, immune phenotypes, and microbial metabolism. The discussion below retains individual examples while distinguishing pathway-associated observations from interventions that test mechanistic dependence. We distinguish single metabolites from anthocyanin mixtures and multicomponent delivery systems (Supplementary Table S4). Puerarin is a major isoflavonoid derived from *Pueraria montana* var. *lobata* (Willd.) Maesen and S.M.Almeida ex Sanjappa and Predeep. Ample evidence associates puerarin treatment with improved intestinal-barrier function, attenuated inflammatory responses, and favorable shifts in gut microbiota and host metabolites during experimental colitis (Wu Y. et al., 2020; Zou et al., 2023). These findings indicate an intricate interplay among epithelial, inflammatory, and microbial vectors, although the precise causal trajectories warrant study-specific interpretation. Quercetin, a flavonoid occurring in multiple plant sources, mitigates UC primarily by activating the aryl hydrocarbon receptor (AHR), upregulating Arnt/NQO1 to diminish reactive ROS and NETs (Wei Q. et al., 2025), inhibiting the NF-κB, cGAS–STING (Gao et al., 2024), and CXCL8–CXCR1/2 pathways (Jiang et al., 2024), repairing TJ proteins to restore the intestinal barrier (Wang X. et al., 2024), and alleviating inflammation, thereby enhancing symptoms of DSS-induced UC. Research indicates that kaempferol can mitigate UC in mice by restoring the intestinal microbiota and inhibiting the LPS–TLR4–NF-κB pathway (Qu S. L. et al., 2021). Researchers engineered a functional chitosan hydrogel to co-deliver rhubarb-derived nanovesicles (RN) and kaempferol to treat UC, with pH/enzyme sensitivity and macrophage-targeting capabilities (Tang L. et al., 2025). Astragalin can mitigate DSS-induced acute UC in mice by correcting gut microbiota dysbiosis, inhibiting NF-κB activation, reducing inflammatory mediators, and enhancing intestinal mucosal-barrier integrity (Peng et al., 2020; Chen et al., 2023). By regulating the gut microbiota, repairing the intestinal barrier, and promoting anti-inflammatory mechanisms, vitexin can alleviate DSS-induced UC (Zhang et al., 2022). Vitexin regulates macrophage polarization, thereby preventing colitis-associated carcinogenesis in mice (Chen Y. et al., 2021). Hypericin treats UC by facilitating autophagy in colonic epithelial cells, modulating MKRN1/PPAR γ signaling, and preserving the Th17/Treg equilibrium to safeguard the intestinal barrier (Cheng et al., 2021). Hyperoside-loaded mixed micelles increased oral bioavailability to 4.15-times that of free hyperoside in the reported experiment (Jin et al., 2024). Nobiletin can modulate intestinal microbiota, mediate HIF-1α to inhibit ferroptosis, restore the colonic mucosal barrier, alleviate UC symptoms, and offer novel targets and concepts for UC treatment (Chen Z. et al., 2025).

Both naringin and naringenin, derived from the TCM *Citrus reticulata* Blanco, protect mice from DSS-induced UC. The latter reduces inflammation and oxidative stress by activating the Nrf2 pathway and blocking NF-κB (Li H. et al., 2025). The former reduces symptoms by preserving the intestinal barrier, reducing inflammation, and controlling the gut microbiota (Cao et al., 2021). Neohesperidin (NEO) mitigates UC by inhibiting the MAPK/NF-κB pathway, reducing inflammatory mediators, increasing intestinal-barrier proteins, and modulating the gut microbiota (Ju et al., 2024). Similarly, some studies synthesize HST-Arg NPs via Mannich reaction and π–π stacking, which target the inflamed colon, scavenge ROS, release NO, alleviate UC better than 5-ASA, and show good gastrointestinal stability and safety (Chi et al., 2024). Isoliquiritin similarly ameliorates TNBS-induced UC in rats by reducing the Disease Activity Index (DAI) score, mitigating body weight loss and colon shortening, decreasing inflammatory mediators, and acting through a caspase-3/HMGB1/TLR4-dependent pathway to inhibit downstream TLR4 signals, thereby alleviating intestinal inflammation and mucosal damage (Miao et al., 2024). Bioinformatics screening and *in vitro* and *in vivo* experiments have shown that quercetin can ameliorate DSS-induced UC in mice by mitigating weight loss, decreasing DAI, alleviating colon shortening, and exerting antioxidant, anti-inflammatory, intestinal barrier-protective, and inhibitory effects on colon cell apoptosis and fibrosis (Wang et al., 2021). Researchers investigated lonicerin along the EZH2/ATG5/autophagy/NLRP3 axis. ATG5 perturbation and EZH2 overexpression experiments supported a role for this pathway in the tested models (Lv et al., 2021). Isorhamnetin mitigates DSS-induced UC by inhibiting ferroptosis in IECs through the activation of the NRF2/HO-1 pathway and iron chelation, with efficacy confirmed in both *in vitro* and *in vivo* studies (Ru et al., 2024). Research indicates that mulberry anthocyanins (MAS) mitigate DSS-induced UC in mice by preserving TJ protein expression in the intestine, modulating inflammatory factor production, suppressing oxidative stress, and rectifying gut microbiota dysbiosis. The high-dose intervention has a more pronounced impact (Mo et al., 2022). Luteoloside may modulate the NCR-ILC3/NCR + ILC3 balance via the Notch signaling pathway, enhance IL-22 production, restore the intestinal barrier, regulate gut microbiota and metabolism, reduce inflammation, and ameliorate weight loss and colon damage, thereby laying the foundation for UC treatment (Yang et al., 2025). Dihydromyricetin (DHM) ameliorates symptoms in UC mice by inhibiting the NLRP3 inflammasome, modulating the gut microbiota, and restoring the intestinal barrier (Jiang et al., 2025). Additionally, another study showed that DHM suppresses NET formation via the HIF-1α/VEGFA pathway, thereby safeguarding the intestinal barrier and emerging as a potential therapeutic candidate for UC treatment (Ma et al., 2024). Apigenin mitigates DSS-induced UC inflammation and intestinal-barrier impairment via modulating PAMP-MRGPRX2-mediated mast cell degranulation (Huang Y. et al., 2025), suppressing ERS in goblet cells (Ji et al., 2025a), and altering the gut microbiota composition (Fu et al., 2022).

Galangin, the primary active compound in *Alpinia officinarum* Hance, may inhibit HSP90β and mitigate UC by modulating fatty acid production and NLRP3 inflammasome activation (Yang L. et al., 2023). Chrysin may be encapsulated in pectin-coated chitosan BSA meta rad nanoparticles to improve gastric protection and mucosal adhesion, thereby enhancing the efficacy of oral therapy for UC (Jori et al., 2025). Relevant studies indicate that phosphatidylserine-functionalized liposomes in microgels facilitate colon-targeted genistein delivery, thereby reducing pro-inflammatory cytokines and promoting colonic mucosal-barrier repair (Yan S. et al., 2023). Additionally, genistein mitigates UC by modulating the gut microbiota and balancing macrophage polarization (Jia et al., 2024). It enhances mitochondrial biogenesis, increases antioxidant activity, and inhibits apoptosis (Alharbi et al., 2024). Acacetin mitigates DSS-induced UC in mice by easing clinical symptoms, suppressing macrophage inflammatory responses and proinflammatory mediators, and correcting gut microbiota dysbiosis to restore microbial balance (Ren et al., 2020). Tricin demonstrates anti-inflammatory effects in LPS-activated RAW264.7 macrophages via the NF-κB pathway and mitigates DSS-induced acute colitis in mice by reducing colonic inflammation and modulating gut microbiota, indicating its potential for treating UC (Li et al., 2021). Eriodictyol shows protective effects against UC in DSS-induced mice and TNBS-induced rats by mitigating inflammation, oxidative stress, and intestinal-barrier damage through the activation of the Shh pathway or inhibition of the TLR4/NF-κB pathway, positioning it as a potential therapeutic agent for UC (Hu L. H. et al., 2021). Tiliroside may ameliorate DSS/TNBS-induced UC in mice by facilitating HIF-1α proteasomal degradation and suppressing macrophage aerobic glycolysis to restore the M1/M2 macrophage equilibrium (Zhuang et al., 2021). According to a study, wogonin may enhance bioavailability and reduce AFM by modifying the efflux transporter BCRP/MRP2 and regulating the Nrf2/TLR4/NF-κB axis to reduce oxidative stress, inflammation, and cell death, thereby exerting an anti-UC effect (Liu W. J. et al., 2025; Zhou et al., 2022). Formononetin is a flavonoid compound derived from *Astragalus membranaceus* (Fisch.) Bge. that can mitigate DSS-induced UC in a dose-dependent manner through the modulation of M1/M2 macrophage polarization (Xiao et al., 2024b), regulation of gut microbiota, enhancement of antioxidant capacity, and promotion of TJ protein expression (Xiao et al., 2024b).

Across flavonoids, inflammatory and barrier-associated responses recur, but not all studies test causality or exposure. Lonicerin was examined in female C57BL/6 mice with 2.5% DSS, with six animals per initial treatment group and a 5-ASA comparator, and the mechanistic experiments included ATG5 perturbation and EZH2-related validation (Lv et al., 2021). This provides more specific support than marker changes alone, while remaining preclinical. The differences between defined flavonoids, anthocyanin mixtures, and loaded formulations preclude a single class-wide efficacy or safety conclusion.

### 6.3 Terpenoids

Terpenoid studies connect redox regulation, regulated cell death, barrier function, and microbial responses. The relevant experiments differ in species, disease induction, and formulation. Food–medicine-derived terpenoids, including oleanolic acid derivatives and the sesquiterpenoid β-caryophyllene, are promising candidates because they can control oxidation, preserve intestinal integrity, and alter gut microbiota. Oleanolic acid 28-O-β-D-glucopyranoside (OAG), derived from *Panax ginseng* C. A. Mey., provides anti-UC benefits through anti-inflammatory, antioxidant, and gut microbiota-modulating mechanisms. In TNBS-induced colitis models, OAG mitigates weight loss, decreases and alleviates histological damage by inhibiting oxidative stress, modulating Nrf2/x-CT/GPX4-mediated ferroptosis, and restoring intestinal epithelial-barrier integrity. Dietary monoterpenes and sesquiterpenes, such as β-caryophyllene from *Syzygium aromaticum* (L.) Merr. and L. M. Perry and (−)-anethole from *Foeniculum vulgare* Mill., have significant anti-inflammatory, antioxidant, and microbiota-modulating activities. β-caryophyllene restores intestinal equilibrium by enhancing anti-inflammatory effects and beneficial mucins, promoting colonic motility, and reducing. Likewise, (−)-fenchone has anti-inflammatory and barrier-protective properties in TNBS-induced UC models (Araruna et al., 2024). Ursolic acid, a potent compound derived from the TCM *Mentha haplocalyx* Briq., was investigated in DSS-induced mouse colitis and SDS-induced intestinal injury in *Drosophila* by modulating gut microbiota, inflammatory and fatty acid metabolism pathways (Sheng et al., 2021), inhibiting JNK/JAK/STAT signaling, and diminishing oxidative stress and immune-cell infiltration (Wei et al., 2022). Zhuang and colleagues recently synthesized betulinic acid-loaded carboxymethyl chitosan/sodium alginate (BA-CS-SA) microspheres. These microspheres were compared with free betulinic acid and showed improved outcomes in mitigating DSS-induced colitis through intestinal-barrier protection and anti-inflammation effects (Zhuang et al., 2025).

Furthermore, 18β-glycyrrhetinic acid derived from *Glycyrrhiza uralensis* Fisch. improved epithelial-barrier integrity in UC through activation of the Wnt/β-catenin pathway, thereby promoting the expression of TJ proteins (Peng et al., 2025). Similarly, in the DSS-induced UC model, pachymic acid mitigated intestinal injury by decreasing pro-inflammatory cytokine expression and alleviating oxidative stress (Zhang D. et al., 2025). Patchouli alcohol is a sesquiterpene found in *Pogostemon cablin* (Blanco) Benth. that ameliorates DSS-induced UC pathology in rodents by suppressing inflammation and minimizing intestinal injury (Wu Z. et al., 2020). Notably, Zhuge et al. demonstrated that geniposide, a principal bioactive constituent of *Gardenia jasminoides* J. Ellis, interacts with KEAP1 to activate the Nrf2/ARE pathway, inhibits NF-κB signaling, and alleviates DSS-induced colitis by reducing oxidative stress, inflammation, and impairment of the intestinal barrier (Zhuge et al., 2023). Meanwhile, the natural iridoid glycoside sweroside alleviates DSS-induced UC by modulating the VEGF and Hippo pathways, inhibiting abnormal angiogenesis of endothelial cells, enhancing intestinal mucosal microcirculation, and restoring intestinal barrier integrity while reducing inflammation (Li L. et al., 2025). Furthermore, Puppala et al. reported that perilla alcohol exhibits anti-inflammatory and antioxidant properties by modulating the TLR4/NF-κB and JAK2/STAT3 pathways, thereby significantly ameliorating DSS-induced UC and associated behavioral and neurotransmitter disturbances in mice subjected to chronic restraint stress (Puppala et al., 2022). Ganoderic acid derived from *Ganoderma lucidum* (Curtis) P. Karst. alleviates inflammatory bowel disease and UC in DSS-induced murine models by modulating gut microbiota composition, strengthening intestinal barrier function through the regulation of TJ proteins (Ye et al., 2025), and influencing tryptophan metabolism, AHR signaling pathways, or beneficial metabolites (Kou et al., 2024). Geniposide enema was also examined in relation to microbial and bile-acid pathways (Zheng et al., 2025), and this route-specific evidence should not be merged with oral dosing results.

Additional terpenoid examples include limonene, evaluated for inflammatory and gastrointestinal outcomes (Adriana Estrella et al., 2021), and 1,8-cineole, investigated in relation to macrophage polarization and the HSP90/NLRP3/SGT1 complex (Ma et al., 2023). Studies of α-terpineol and ar-turmerone examined inflammatory, apoptosis-associated, or microbial responses (Khan et al., 2023; Li C. et al., 2022). Asperuloside is an iridoid glycoside investigated through Nrf2/HO-1 and NF-κB-associated responses (Chen Y. E. et al., 2021), while β-myrcene was examined in relation to MAPK/NF-κB signaling (Almarzooqi et al., 2022). These examples are grouped with terpenoids consistently in Supplementary Table S5. The terpenoid literature supports several proposed routes for epithelial protection, but results obtained with different induction protocols and delivery systems are not directly comparable. Geniposide is an iridoid glycoside and is considered here separately from lignans. Its cellular Nrf2-intervention experiments provide pathway-dependence evidence, whereas changing the administration route or combining it with a carrier requires separate exposure and safety evaluation.

### 6.4 Polysaccharides

Polysaccharides are macromolecular preparations. Their biological effects may depend on molecular-weight distribution, monosaccharide composition, linkage structure, purity, and residual co-extractives. They are, therefore, considered separately from chemically defined small-molecule metabolites. The main recurring modules are microbial metabolism, immune responses, and barrier function (Supplementary Table S6).

Purslane polysaccharides have shown efficacy in mitigating DSS-induced UC by fostering intestinal homeostasis, augmenting TJ protein expression, and reducing pro-inflammatory cytokine levels (Ning et al., 2024). Such interplay of immunomodulation and microbiota restoration underscores the relevance of polysaccharide preparations in UC management. Polysaccharides, including Astragalus polysaccharide, Codonopsis polysaccharide, and Coix seed polysaccharide, not only restore gut homeostasis but also ameliorate experimental UC by modulating immune responses and promoting SCFA synthesis. APS mitigated DSS-induced colitis by regulating the Th17/Treg equilibrium via the microbiota-dependent TIGIT/CD155 signaling pathway (Wan J. et al., 2024). Furthermore, APS corrected microbial dysbiosis by increasing SCFA-producing bacteria, thereby increasing intestinal SCFA levels, which improved immune homeostasis and mucosal repair (Zhang H. et al., 2025). Similarly, *Codonopsis pilosula* polysaccharide modulated the SCFA-GPR-NLRP3 pathway while inhibiting inflammation and epithelial injury (Zhou et al., 2025). Polysaccharides from *Coix lacryma-jobi* mitigated UC by restoring gut microbial diversity and enhancing intestinal metabolic equilibrium, perhaps by augmenting SCFA synthesis (Ji et al., 2025b).

Dioscorea polysaccharide restored gut microbial diversity while reducing oxidative stress and metabolic dysregulation in DSS-treated rats, an action associated with maintaining gut microecological stability and TJ integrity (Zhang Z. et al., 2025). Mulberry polysaccharides mitigate gut inflammation in acute inflammatory bowel disease models by modulating macrophage and T-cell activities, showcasing their potential to target both innate and adaptive immune pathways (Feng et al., 2021). Meanwhile, polysaccharides derived from *Platycodon grandiflorus* (Jacq.) A. DC. have been shown to modulate mesenteric lymphatic circulation, decrease immune-cell infiltration and inflammatory cytokine levels in colonic tissue, and ameliorate UC (Liu et al., 2023). *Lycium barbarum* L.-derived acidic polysaccharides exhibit notable therapeutic effects on DSS-induced UC, primarily through the modulation of intestinal microbiota (Liu et al., 2025a). Poria cocos polysaccharides exhibited protective benefits by mitigating gut dysbiosis and enhancing beneficial Firmicutes populations, hence improving gut homeostasis in DSS-treated rats (Wan Q. et al., 2024). Polysaccharides obtained from steamed *Polygonatum sibiricum* Redouté enhance the expression of TJ proteins, including ZO-1 and occludin, while reducing pro-inflammatory cytokines such as TNF-α and IL-6, modulating the gut microbiota, and showing significant therapeutic effects in protecting the intestinal mucosal barrier (Li D. et al., 2025).

In addition to their microbiota-centric effects, polysaccharides from *Houttuynia cordata* Thunb. and *Phyllanthus emblica* L. have been investigated for metabolic and immunomodulatory effects. Polysaccharides from *Houttuynia cordata* markedly suppress NF-κB activation, enhance ZO-1 and MUC2 expression to safeguard the intestinal barrier, and modulate gut microbiota. Its mechanism is intricately linked to the preservation of intestinal homeostasis (Cen et al., 2022). Concurrently, *Phyllanthus emblica* L. polysaccharides in Yuganzi enhance colitis treatment by modulating intestinal microbiota composition; influencing pyrimidine, β-alanine, and purine metabolic pathways; regulating inflammatory cytokine levels; and repairing the intestinal barrier, thereby contributing to optimal immune regulation (Li X. et al., 2024). *Crataegus pinnatifida* polysaccharides augmented arginine metabolism, thereby supporting immune homeostasis and epithelial regeneration (Wei F. H. et al., 2025). Specifically, seabuckthorn polysaccharides and alginate microspheres have distinct transport and targeted action mechanisms. Seabuckthorn polysaccharides significantly alleviated colonic damage by reducing inflammatory cytokines and enhancing serum antioxidant capacity (Ouyang et al., 2024). Researchers used an alginate microsphere system to deliver epidermal growth factor to the intestine (Chen Z. et al., 2024). Without an appropriate carrier control, its effects cannot be attributed to alginate alone. *Angelica sinensis* polysaccharide nanoparticles, engineered for colon-specific delivery, demonstrably mitigate colitis through the inhibition of pro-inflammatory cytokine release, the reduction of oxidative stress, the preservation of the intestinal mucosal barrier, and the modulation of the gut microbiota (Xu Z. et al., 2023). Dandelion polysaccharides alleviate UC through both gut microbiota-dependent and -independent mechanisms, including activation of the Nrf2/HO-1/NQO-1 pathway to mitigate oxidative stress, inhibition of intestinal epithelial ferroptosis, modulation of gut microbiota composition, and enhancement of short-chain fatty acid production (Yan X. et al., 2023).

Notably, turmeric polysaccharide enhances tryptophan metabolism to generate indole-3-acetic acid (IAA) by promoting the growth of probiotics such as *Lactobacillus*, activates the AHR/IL-22 pathway, upregulates the expression of TJ proteins ZO-1 and occludin to restore the integrity of the intestinal barrier, and decreases the levels of inflammatory mediators in colitis (Yang et al., 2021). Furthermore, *Dendrobium officinale* polysaccharides augment the proliferation of *Lactobacillus*, modulate purine and arachidonic acid metabolism, elevate SCFA levels, and promote goblet-cell density to preserve intestinal-barrier integrity (Zeng et al., 2024). *Panax quinquefolius* polysaccharides (WQP) mitigate DSS-induced UC in murine models. High-dose WQP increases Bacteroidetes, decreases Firmicutes, enhances SCFAs, equilibrates inflammatory cytokines, upregulates TJ proteins, fortifies the intestinal barrier, and alleviates UC symptoms (Ren et al., 2023). *Rehmannia glutinosa* polysaccharide mitigates colitis by enhancing the genera *Lactobacillus* and *Alistipes*, facilitating the synthesis of SCFAs, suppressing the activation of the NF-κB signaling pathway, diminishing the secretion of inflammatory mediators, and consequently restoring the intestinal barrier (Lv et al., 2023). Ophiopogonis Radix fructan-selenium nanoparticles were evaluated using antioxidant, inflammatory, and cancer-cell endpoints (Jia et al., 2025). Chemical radical scavenging and cancer-cell apoptosis alone do not establish UC treatment or cancer prevention in patients.

Polysaccharide studies are especially sensitive to material comparability and dietary context. Preparations sharing a botanical name may differ in molecular-weight distribution, branching, purity, or co-extracted substances. Fermentation-associated effects should be distinguished from direct host-cell actions, and the same microbial taxon need not change in the same direction across models. These limitations prevent extrapolation from one preparation to all polysaccharides from a species.

### 6.5 Saponins

Saponins and related sapogenins have been investigated in epithelial injury, inflammatory signaling, cellular stress, and immune regulation. Chemically defined substances, such as astragaloside IV and mogroside V, are distinguished from total saponin fractions (Supplementary Table S7). The following studies examine distinct saponins, a total saponin fraction, and a related sapogenin in experimental colitis. By preventing WDR5-mediated H3K4me3 alteration of ENO1 and obstructing the PI3K/AKT pathway to restore the intestinal epithelial barrier, astragaloside IV reduces UC (Wu S. X. et al., 2025; Zhang T. et al., 2024). Codonopsis whole saponins prevent UC by inhibiting NF-κB signaling, controlling inflammatory cytokines, and having anti-lipid peroxidation properties (Liu J. et al., 2021). Mogroside V improved ERS-related and barrier outcomes when orally administered at doses of 25, 50, and 100 mg/kg in female C57BL/6J mice exposed to 3% DSS for 7 days (\(n = 6\) per initial group), an effect weakened by tunicamycin (Tan et al., 2024). Dioscin was associated with improved intestinal-barrier function, reduced NF-κB/MAPK/NLRP3 signaling, and modulated autophagy through the AMPK–mTOR axis (Cai et al., 2021; Li H. et al., 2022). Meanwhile, platycodin D attenuated experimental UC and altered macrophage polarization, accompanied by increased PI3K/AKT phosphorylation and reduced NF-κB activation; however, cellular AMPKα knockdown weakened its effects on polarization (Guo et al., 2021). Ginsenoside Rb1 was investigated in relation to VDR, PPARγ, and NF-κB signaling (Zhou Y. et al., 2024), whereas Rg1 studies examined microbial metabolism and Bcl-6/Blimp-1-associated memory T-cell responses (Zhang S. et al., 2025; Zhong et al., 2023). Ruscogenin, a steroidal sapogenin, reduced caspase-1-dependent pyroptosis and the TLR4/NF-κ B-associated responses (Li J. et al., 2024). Finally, ophiopogonin D improved inflammatory and barrier-related outcomes and altered gut microbiota in experimental colitis, although a separate propionate intervention that also improved colitis did not establish that propionate directly mediated the effects of ophiopogonin D (Zhang X. et al., 2024).

These studies converge on inflammation and epithelial outcomes, but their mechanisms should not be treated as interchangeable. In particular, measurements in different cell populations can show different directions of PI3K/AKT regulation. Such findings require cell-specific interpretation rather than a universal activation or inhibitory mechanism. Total saponin fractions also require compositional standardization before attributing their effects to a defined molecule.

### 6.6 Phenols

Phenolic metabolites are discussed separately from phenolic-enriched mixtures, such as purple yam polyphenols (Supplementary Table S8). Specifically, purple yam polyphenol extracts mitigate UC and colitis-associated colorectal cancer by inhibiting the NF-κB/p65 and STAT3 signaling pathways (Yang et al., 2023a). Gallic acid provides protective benefits through multiple mechanisms, including regulating gut microbiota and bile-acid metabolism to enhance group-3 innate lymphoid cells, along with inhibiting the NLRP3 inflammasome (Leng et al., 2024; Yu S. et al., 2023). Ellagic acid (EA) alleviates UC by inhibiting the ROS/NLRP3 pathway and modulating intestinal microbiota (Xiong et al., 2025). Chebulagic acid mitigates oxidative stress and inflammation by blocking the MAPK/NF-κB pathways and activating NRF2/HO-1, while also modulating gut microbiota equilibrium (Zhang H. et al., 2025). Furthermore, 6-gingerol inhibits ferroptosis by modulating ALOX5, ALOX15, and PTGS2, and it regulates the metabolism of linoleic acid and arachidonic acid (Li et al., 2024a). Furthermore, the active constituents ferulic acid and caffeic acid in the medicinal and edible Chinese medication Goji berries also modulate UC. Among these, ferulic acid exhibits anti-inflammatory, antioxidant, and anti-apoptotic properties by inhibiting the LPS–TLR4–NF-κB and NF-κB–iNOS–NO signaling pathways. It also influences the TXNIP/NLRP3 pathway to mitigate injury to intestinal endothelial cells (Ghasemi-Dehnoo et al., 2023; Yu T. Y. et al., 2023). Caffeic acid was associated with protection in a *Drosophila* intestinal-injury model, including attenuation of the Toll/Imd signaling cascade and mitochondrial-mediated apoptosis, modulation of gut microbiota equilibrium, reduced pathogenic bacterial proliferation, and restoration of intestinal-barrier integrity (Xiu et al., 2025). Gastrodin, a phenolic glycoside, was associated with *Akkermansia muciniphila* and microbial tryptophan metabolism and AhR/NLRP3 signaling in separate reports (Zhang Y. et al., 2025; Zhang X. et al., 2025). These microbial and host responses require interpretation in the context of each study.

In addition, rosmarinic acid regulates the PI3K/AKT/Nrf2 pathway to enhance TJ protein production, thereby augmenting intestinal-barrier integrity and mitigating mucosal inflammatory infiltration (Tang R. et al., 2025). Resveratrol modulates the MDM2/P53/PUMA pathway to prevent excessive colonocyte apoptosis, improve intestinal-barrier integrity, and alleviate UC-related pathological damage (Tang X. et al., 2025). Vanillic acid exerts dual therapeutic actions: first, it modulates gut microbiota, promotes M1 to M2 macrophage polarization, and inhibits the cPLA2–COX2–PGE2 pathway to alleviate inflammation (Zhao et al., 2025); second, it interacts with CA9 and regulates the STIM1–INSIG2–SREBP1–SCD1 axis to suppress ferroptosis, thereby restoring intestinal epithelial homeostasis (Ni et al., 2024). Curcumin, a well-defined phenolic molecule, has been investigated in distinct inflammatory and immune pathways: it alleviates UC severity by inhibiting the sphingosine kinase 1/NF-κB signaling pathway and alters regulatory B-cell activity via the TLR/MyD88 pathway to reestablish immunological equilibrium (Zhang C. et al., 2025; Huang et al., 2023). Protocatechuic acid mitigates DSS-induced UC in mice via modulating gut microbiota equilibrium and inhibiting ferroptosis, a critical pathogenic contributor to intestinal epithelial-cell injury (Yang et al., 2023b). Moreover, acteoside, an essential phenolic monomer, alleviates DSS-induced UC by modulating the HO-1/HMGB1 signaling pathway, thereby suppressing excessive inflammatory responses (Guo et al., 2022). Isochlorogenic acid A was investigated in relation to STAT3/NF-κB signaling, inflammatory-cell infiltration, and tight-junction proteins (Tang et al., 2023). Chlorogenic acid nanoparticles were evaluated for colon retention, antioxidant responses, and barrier outcomes (Hu et al., 2024). Supplementary Table S8 includes these phenolic interventions. Nanoparticle findings remain formulation-specific.

Phenolic interventions involve overlapping redox, inflammatory, epithelial, and microbial pathways. Repeated downstream changes across metabolites identify hypotheses for convergence, not proof that the molecules share a direct target or act synergistically. The *Drosophila* and tumor-related experiments in this literature are mechanistic or adjacent evidence and should not be presented as direct demonstrations of efficacy in human UC.

### 6.7 Coumarins

Coumarins provide examples involving MAPK signaling, immune-cell responses, and barrier repair. Their interpretation is organized around the proposed pathway and associated functional outcome, distinguishing marker changes from pathway dependence (Supplementary Table S9).

Coumarin metabolites, including imperatorin, isoimperatorin, scopoletin, isofraxidin, and 7-hydroxycoumarin, have been investigated in experimental colitis through pathways associated with inflammation, immune regulation, and barrier function. However, modulation of several pathways does not by itself establish pharmacological synergy or clinical efficacy. Specifically, 7-hydroxycoumarin mitigates UC by blocking the MAPK pathway, decreasing inflammatory cytokines, and improving gut microbiota dysbiosis (Liu X. et al., 2024). Imperatorin was studied at 15, 30, and 60 mg/kg in TNBS-treated rats, with assessments on days 3, 7, and 14. The reported Nrf2/ARE/HO-1-associated response increased on day 3 but decreased on day 14 (Luo and Luo, 2021). This time-dependence should not be reduced to a single activating or inhibitory arrow. Isoimperatorin stimulates Treg cell formation and enhances intestinal mucosal repair by expediting colonic epithelial-cell migration, exhibiting a Treg-dependent therapeutic effect (Fang et al., 2025). Isofraxidin inhibits IL-17 signaling by targeting the G-protein-coupled receptor S1PR1, consequently reducing intestinal inflammation and epithelial-cell death (Huang et al., 2024).

Additionally, scopoletin activates PPAR*γ*, inhibits the NF-κB pathway and NLRP3 inflammasome, modulates the gut microbiota, and improves intestinal-barrier integrity by upregulating TJ proteins (Alqudah et al., 2025). These reports describe effects in experimental colitis models, but the extent of mechanistic validation varies. Interpretation requires consideration of the cell type, intervention timing, comparator, and functional outcome. Pathway-dependent immune responses provide different evidence from changes in total protein expression, and differences in response direction should not be resolved by assuming a universal class effect.

### 6.8 Lignans

Lignans are a class of phenylpropanoid-derived metabolites, distinct from the structural polymer lignin. The following studies address inflammatory, epithelial, and tumor-related endpoints (Supplementary Table S10). Dehydrodiisoeugenol specifically targets NOD2 to bidirectionally suppress NF-κB/MAPK-mediated inflammation at low concentrations and promote autophagy in CRC cells at high concentrations (Yi et al., 2025). Sesamin improves UC by regulating the DUSP1/ERK feedback loop and restoring gut microbial equilibrium (Cao et al., 2025). Syringaresinol enhances intestinal epithelial-barrier integrity and reduces inflammation by blocking the PI3K-Akt/MAPK/Wnt signaling pathway (Liu Y. et al., 2024). Moreover, schisandrin safeguards against UC by inhibiting the SGK1/NLRP3 pathway and modulating gut microbiota and bile-acid metabolism (Wang et al., 2023b).

Lignan studies involve different endpoints, including inflammation, barrier function, and tumor-related processes. Antiproliferative or pro-apoptotic effects in cancer cells cannot be equated with protection of non-neoplastic intestinal epithelium. Cross-study synthesis, therefore, requires separating colitis and cancer outcomes and confirming the dose and cell type.

### 6.9 Others

Other metabolites include chemically diverse substances that should not be treated as one pharmacological class (Supplementary Table S10). Initially, myristicin suppresses ERS markers and the NF-κB pathway while enhancing the Nrf2/HO-1 axis, thereby mitigating oxidative damage and DNA fragmentation (Ismail Abo El-Fadl and Mohamed, 2022). Cinnamaldehyde inhibits Th17 cell differentiation through the S1P2–Rho–GTP signaling pathway and modulates the inflammatory response by regulating the balance of lncRNA H19/MIAT (Qu Y. et al., 2021). Sodium houttuyfonate promotes β-glucan exposure in *Candida albicans* (Ma et al., 2021), stimulates dectin-1/TLR2/4-mediated fungus elimination, and modulates the TNF-α/IL-10 equilibrium (Cheng T. et al., 2023). Ligustilide directly interacts with the His386 site of EGR1, blocking its nuclear translocation and association with the ADAM17 promoter, and this impedes the EGR1–ADAM17–TNF-α pathway, which inhibits macrophage-driven intestinal inflammation and restores intestinal mucosal-barrier integrity (Li J. et al., 2025).

Shikimic acid mitigates UC by suppressing NF-κB/MAPK pathways, improving intestinal-barrier integrity, and regulating gut microbiota (Li X. et al., 2023). Sulforaphane reduces oxidative stress and inhibits NLRP3 inflammasome activation, thereby suppressing the caspase-1/IL-1β axis (Zhou Z. J. et al., 2024). Panaxynol, a polyacetylene rather than a terpenoid, was investigated in acute and chronic mouse colitis with structural, microbial, and immune endpoints (Bullard et al., 2024). This remaining group is chemically heterogeneous and does not imply a shared mechanism. Ligustilide was investigated using EGR1-related mechanistic experiments (Li H. et al., 2025), while the sulforaphane study included oral doses of 25 and 50 mg/kg/day and sulfasalazine as a positive comparator over 7 days (Zhou Y. et al., 2024). These examples illustrate the need to connect the proposed pathway to a specified dose, model, and functional response rather than infer class-wide activity.

## 7 Extracts from medicinal and food resources

Extracts provide preparation-specific evidence and are considered separately from defined metabolites (Supplementary Table S12). Seabuckthorn (*Hippophae rhamnoides L.*) berries significantly reduced DSS-induced colitis by increasing butyrate biosynthesis mediated by *Faecalibaculum rodentium*, as shown by improved clinical scores and decreased colonic inflammatory markers (Song et al., 2025). Furthermore, by addressing both immune signaling and microbiota modulation, *Glycyrrhiza uralensis* Fisch. has demonstrated strong effects on UC. It works by significantly inhibiting NF-κB signaling, which reduces the recruitment of inflammatory cytokines such as TNF-α and IL-1β (Shi et al., 2022). By controlling the JNK/TLR4 pathway, *Eucommia ulmoides* Oliv. aqueous extract reduces cognitive dysfunction in mice with DSS-induced colitis. It reduces oxidative stress, inflammation, and apoptosis and enhances intestinal barrier, mitochondrial, and cholinergic function, fecal SCFA, and colitis symptoms (Lee et al., 2024). *Poria cocos* (Schw.) Wolf alleviates DSS-induced UC by lowering pro-inflammatory cytokines, restoring the intestinal barrier, and modulating the gut microbiota through NF-κB signaling (Song et al., 2024). In TNBS-ethanol-induced UC model rats, *Codonopsis pilosula* (Franch.) Nannf. aqueous extract significantly decreased inflammatory markers such as IL-6, inhibited PI3K/Akt pathway overactivation, and encouraged mucosal repair (Li et al., 2024b). By inhibiting inflammatory signaling via PLCB2 and preventing pyroptosis, *Astragalus membranaceus* (Fisch.) Bge. extract, on the other hand, showed its effectiveness in improving intestinal epithelial stability (Shen et al., 2024).

*Portulaca oleracea* L. ethanol and water extracts with varying molecular weights can reduce colitis in mice by regulating gut microbiota, acting as an antioxidant, and reducing pro-inflammatory factors such as TNF-α and IL-17. These effects are statistically significant (Alfwuaires et al., 2021; Ning et al., 2023). By blocking the MAPK pathway, restoring the intestinal epithelial barrier, and lowering inflammatory factor levels, *Amomum villosum* extract helps mice with DSS-induced colitis (Choi et al., 2025). Zhao et al. found that *Piper wallichii* ethanol extract ameliorates DSS-induced murine UC, identifying 14 components via UPLC-QE-Orbitrap-MS, acting by protecting the intestinal barrier, regulating inflammation/immunity, and inhibiting the TLR4/NF-κB/COX-2 pathway (Zhao et al., 2023). Mume fructus extract promoted colonic mucosal healing in DSS-induced colitis by enhancing cPLA2-mediated LPC synthesis and epithelial migration, with 5-ASA serving as the positive control in the experimental setup (Zhang J. et al., 2023).

By modifying inflammatory and genetic pathways, *Morus macroura* Miq. fruit extract was shown to control miRNA-223 expression and inhibit the TNF-α/NF-κB/NLRP3 axis, thereby preventing acetic acid-induced colitis (Salama et al., 2022). By boosting the production of SCFAs such as acetate and propionate and controlling gaseous homeostasis, *Polygonatum odoratum* (Mill.) Druce methanol extract (YZM) showed that it could modulate microbial dysbiosis and ultimately improve epithelial-barrier integrity (Ye Z. et al., 2022). By increasing antioxidant activity and decreasing cellular biomarkers of oxidative stress in TNBS-induced colitis models, *Gardenia jasminoides* J. Ellis fruit extract also significantly reduced mucosal inflammation (Liu X. F. et al., 2021).

The extract literature connects material composition to inflammation, microbial metabolism, and epithelial repair. However, preparations from the same species cannot be assumed equivalent when the plant part, solvent, extraction conditions, or chemical profiles differ. The evidence supports preparation-specific hypotheses. It does not establish that all extracts from a resource are suitable for food use or that an observed effect is attributable to a named metabolite.

## 8 Combined interventions and delivery systems

Combined interventions require explicit identification of the active materials and the delivery system’s role. Piperine–glycyrrhizic acid nanocrystals were investigated for macrophage-associated effects (Wu et al., 2024). Rhein and emodin were incorporated into a colon-directed system with β-1,3-D-glucan (Wang T. et al., 2024). Other reports examined *Platycodon grandiflorus* polysaccharides with hesperidin (Liu et al., 2025b), sodium alginate with chlorogenic acid (Niu et al., 2022), and *Astragalus membranaceus* with *Codonopsis pilosula* polysaccharides (Tang et al., 2021). These interventions differ in material complexity and should be distinguished as combinations of defined metabolites, macromolecular preparations, or delivery systems.

Evidence of a greater response to a formulation must be separated from evidence of pharmacological synergy. Appropriate comparisons include the individual agents, the unformulated mixture, and the carrier alone, with the dose and exposure considered. A carrier can alter retention, release, or uptake, and some carriers may have their own biological effects. Table 1 summarizes the interpretation of these combinations, while Supplementary Table S13 retains the detailed inventory.

**TABLE 1:** Combined interventions and limits of synergy claims.

Intervention	Material category	Reported focus	Critical comparison
Piperine + glycyrrhizic acid (Wu et al., 2024)	Nanocrystal combination	Macrophage-associated and inflammatory effects	Confirm single-agent, physical-mixture, and formulation controls before quantifying synergy
Rhein + emodin (Wang T. et al., 2024)	Combination in glucan-based system	Inflammatory, microbial, and immune responses	Separate combined-agent effects from carrier and exposure effects
Platycodon polysaccharides + hesperidin (Liu et al., 2025a)	Macromolecular preparation + metabolite	Immune, oxidative, and barrier endpoints	Define polysaccharide preparation and inspect dose-matched comparators
Sodium alginate + chlorogenic acid (Niu et al., 2022)	Polymer-associated preparation	Inflammatory, oxidative, and microbial outcomes	Clarify carrier *versus* active-material contribution
Astragalus + Codonopsis polysaccharides (Tang et al., 2021)	Two polysaccharide preparations	AhR/IL-22, microbial metabolites, and barrier outcomes	Preparation identity and interaction evidence required

## 9 Discussion

### 9.1 Convergent mechanisms and strength of evidence

Across chemical classes, inflammatory signaling, redox regulation, epithelial repair, and microbial metabolism emerge as interconnected processes (Figure 2). Their convergence supports investigating interventions that address multiple components of multifactorial UC, although shared downstream responses do not identify common direct targets or establish synergy or superiority over single-target therapy. Pathway perturbation provides stronger mechanistic support: ATG5-dependent effects of lonicerin (Lv et al., 2021), Nrf2 inhibition in geniposide experiments (Zhuge et al., 2023), and reversal of mogroside V protection by an ERS activator (Tan et al., 2024) help connect molecular responses with functional outcomes (Table 2).

**FIGURE 2:**
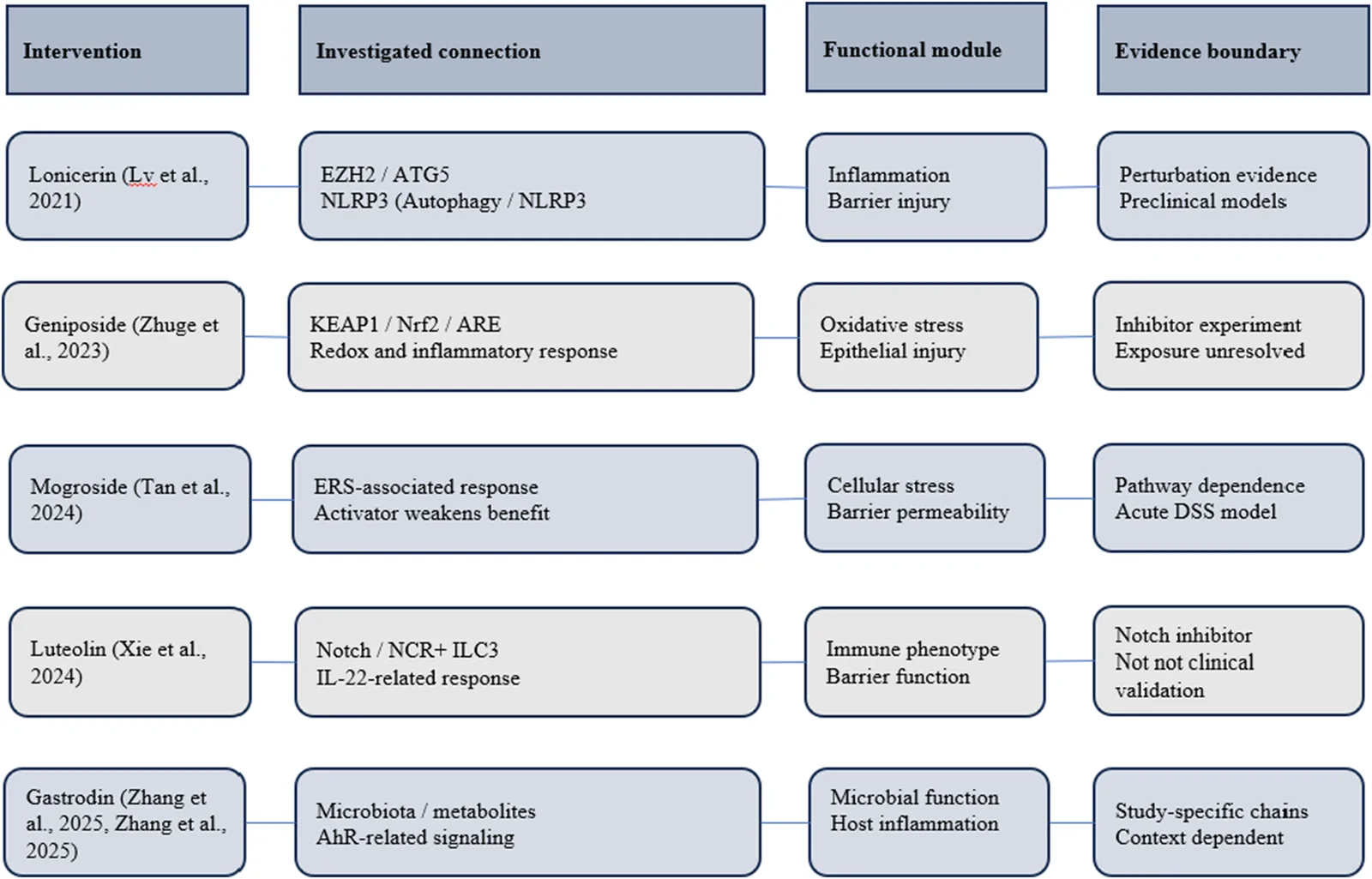
Selected intervention chains and evidential limits. Each row is a study-specific chain, which is not evidence that all agents share a direct target. Multi-pathway responses do not establish pharmacological synergy. Clinical efficacy and safety cannot be inferred from these mechanistic links alone.

**TABLE 2:** Representative mechanistic evidence and interpretation.

Intervention	Experimental context	Mechanistic or functional result	Interpretation and limit
Lonicerin (Lv et al., 2021)	Female C57BL/6; DSS; 3/10/30 mg/kg; initial n = 6/group; 5-ASA control	EZH2/ATG5-related intervention experiments; inflammatory and colitis outcomes	Pathway and target-related validation; acute animal evidence, not clinical efficacy
Geniposide (Zhuge et al., 2023)	DSS mice and LPS-treated Caco-2 cells	Nrf2/ARE-associated response; ML385 abolished cellular protection	Inhibitor supports pathway involvement; human exposure not established
Mogroside V (Tan et al., 2024)	Female C57BL/6J; 3% DSS; oral 25/50/100 mg/kg; 7 days; n = 6/group	Reduced ERS and inflammatory readouts; improved barrier endpoints; tunicamycin weakened benefit	Dose–response and perturbation; short-term model; reporting inconsistencies require care
Luteolin (Xie et al., 2024)	DSS mouse model; Notch inhibitor comparison	NCR + ILC3, IL-22, and barrier-related responses attenuated by LY-411575	Pathway dependence; docking does not establish affinity
Quercetin or chlorogenic acid (Maslin et al., 2022)	Separate dietary arms with 6% pectin; 0.45% quercetin or 0.05% chlorogenic acid	Molecular responses without overt injury protection	Negative functional result; not a combined-agent experiment

Most evidence, nevertheless, remains preclinical. Cellular experiments clarify specific relationships, whereas animal models capture integrated responses whose relevance depends on the disease stage and treatment timing. The frequent use of DSS models and male animals limits generalization. Clinical findings for formulations require separate interpretation and cannot establish the efficacy or mechanism of individual constituents. Non-randomized allocation in the Shenling Baizhu San cohort further limits causal inference (Jiao et al., 2022). Heterogeneous preparations, variable reporting of randomization and blinding, and possible publication bias constrain this narrative synthesis. Cross-study comparisons, therefore, require treatment conditions, sample sizes, and effect estimates with uncertainty, rather than significance alone. A pooled efficacy ranking is not justified.

### 9.2 Microbiota findings and reproducibility

Microbial findings appear more context-dependent than the recurrent improvements in inflammation and barrier function. *Phyllanthus emblica* and *Panax quinquefolius* polysaccharides produced different directions of phylum-level change despite reported benefit (Li F. et al., 2024; Ren et al., 2023), arguing against a universal beneficial Firmicutes/Bacteroidetes ratio. Diet, housing, strain, sex, induction protocol, and sampling methods may contribute to these differences. SCFAs, tryptophan metabolites, and bile acids provide a functional basis for comparison, but the associations require support from depletion, transfer, or metabolite/receptor interventions before mediation can be inferred. Reproducibility across comparable conditions remains uncertain. The lack of protection against overt injury in separate quercetin and chlorogenic acid arms using pectin-containing diets also shows why favorable molecular readouts must be considered alongside tissue outcomes (Maslin et al., 2022).

### 9.3 Exposure and clinical translation

Translation depends on whether active exposures can be achieved safely. Absorption, metabolism, plasma half-life, and tissue distribution remain unevenly characterized across the reviewed materials. Low plasma exposure may coexist with local intestinal activity, but neither local exposure nor achievable *in vitro* concentrations can be assumed. Hyperoside mixed micelles increased oral bioavailability 4.15-fold relative to free hyperoside (Jin et al., 2024), providing formulation-specific pharmacokinetic support. For the other delivery approaches summarized in Table 3, improved release, retention, or disease outcomes alone do not establish an exposure advantage or readiness for clinical testing.

**TABLE 3:** Delivery strategies and pharmacokinetic interpretation.

Intervention	Available evidence	Interpretation	Information needed
Hyperoside mixed micelles (Jin et al., 2024)	Male SD rats; 300 mg/kg orally; n = 5/group. Micelles vs. free drug: AUC_0_–t, 10.30 vs. 2.48 μg·h/mL; Cmax, 1.41 vs. 0.73 μg/mL (both P < 0.05); t_1_/_2_, 10.00 vs. 9.31 h. Mouse oral LD_50_: 3.349 g/kg	4.15-fold relative oral exposure; absolute bioavailability and long-term safety remain unestablished	Intestinal parent/metabolite exposure, PK in colitis, and repeated-dose safety
Ramulus Mori alkaloid microspheres (Wang M. et al., 2025)	Greater IR-820 fluorescence retention at 12 h–24 h than free dye; enhanced adhesion to inflamed colon. Ten-day oral dosing caused no evident weight loss or organ histological injury	Supports formulation retention and short-term tolerability; does not quantify alkaloid exposure	Local/systemic alkaloid PK and longer-term safety
Kaempferol-containing hydrogel (Tang X. et al., 2025)	Fluorescently labeled formulation showed sustained colonic accumulation through 24 h and low blood fluorescence in colitic mice	Tracer distribution does not establish kaempferol exposure; effects involve both kaempferol and rhubarb-derived vesicles	Kaempferol/metabolite PK, quantitative tissue exposure, and repeated-dose safety
Genistein delivery system (Yan H. et al., 2023)	Colonic Cy7 fluorescence persisted to 48 h. Organ histology and ALT/AST/BUN showed no evident abnormalities after the 21-day regimen	Supports prolonged tracer retention and short-term tolerability; genistein bioavailability was not quantified	Parent/metabolite PK, intestinal exposure, and longer-term safety
Betulinic acid microspheres (Zhuang et al., 2025)	Greater release in simulated colonic fluid; improved colitis outcomes *versus* free betulinic acid at an equivalent oral dose of 20 mg/kg/day for 14 days	Supports formulation-related efficacy; increased tissue exposure or bioavailability was not demonstrated	Comparative plasma/colonic PK, carrier-only controls, and repeated-dose safety

Hyperoside PK values are the means (micelles *versus* free drug); LD_50_ refers to the micellar formulation. Fluorescence retention does not quantify drug exposure. The other four studies did not report conventional drug PK parameters; short-term tolerability does not establish long-term safety.

The next step is to connect reproducible functional benefit with characterized preparations, relevant parent and metabolite exposure, and safety over the intended treatment duration. Delivery and combination studies need dose-matched comparators and carrier controls to distinguish pharmacokinetic effects from pharmacodynamic interaction. Food use does not establish the safety of concentrated extracts, purified metabolites, or novel carriers, particularly with concomitant medication. Defined formulations with adequate exposure and tolerability data should be prioritized for controlled clinical studies using meaningful disease outcomes.

## 10 Conclusion

Natural products associated with resources used in foods and medicines have been investigated across interconnected UC-related pathological modules. The evidence supports candidate mechanisms and experimentally testable hypotheses, predominantly in preclinical systems. Chemically defined metabolites, macromolecular fractions, extracts, and formulations require separate interpretation. More convincing translation will depend on accurate material characterization, reproducible functional effects, causal mechanism testing, and exposure-informed safety and efficacy studies.

## Glossary

5-ASA: 5-aminosalicylic acid
AhR: aryl hydrocarbon receptor
Akt: protein kinase B
AMPK: AMP-activated protein kinase
ARE: antioxidant response element
ATG5: autophagy-related protein 5
cGAS: cyclic GMP–AMP synthase
DAI: disease activity index
DSS: dextran sulfate sodium
ERS: endoplasmic reticulum stress
EZH2: enhancer of zeste homolog 2
GPX4: glutathione peroxidase 4
HIF-1α: hypoxia-inducible factor 1α
HMGB1: high-mobility group box 1
HO-1: heme oxygenase-1
IECs: intestinal epithelial cells
ILC3: group 3 innate lymphoid cell
KEAP1: Kelch-like ECH-associated protein 1
LC3: microtubule-associated protein 1 light chain 3
MAPK: mitogen-activated protein kinase
MDA: malondialdehyde
MLCK: myosin light chain kinase
MPO: myeloperoxidase
mTOR: mechanistic target of rapamycin
MyD88: myeloid differentiation primary response 88
NETs: neutrophil extracellular traps
NF-κB: nuclear factor kappa B
NLRP3: NLR family pyrin domain-containing 3
NQO1: NAD(P)H:quinone oxidoreductase 1
Nrf2: nuclear factor erythroid 2-related factor 2
PI3K: phosphatidylinositol 3-kinase
PLGA: poly(lactic-co-glycolic acid)
PPARγ: peroxisome proliferator-activated receptor gamma
SCFAs: short-chain fatty acids
SLC7A11: solute carrier family 7 member 11
STING: stimulator of interferon genes
TJ: tight junction
TNBS: 2,4,6-trinitrobenzenesulfonic acid
TXNIP: thioredoxin-interacting protein
VDR: vitamin D receptor
ZO-1: zonula occludens-1
ALT: alanine aminotransferase
AST: aspartate aminotransferase
**AUC**_**0**_ **–t**: area under the concentration–time curve from time zero to the last measured time point
BUN: blood urea nitrogen
Cmax: maximum concentration
**LD**_**50**_: median lethal dose
PK: pharmacokinetics
**t**_**1**_ **/**_**2**_: elimination half-life

## References

[B1] Adriana Estrella G. R. María Eva G. T. Alberto H. L. María Guadalupe V. D. Azucena C. V. Sandra O. S. (2021). Limonene from Agastache mexicana essential oil produces antinociceptive effects, gastrointestinal protection and improves experimental ulcerative colitis. J. Ethnopharmacol. 280, 114462. 10.1016/j.jep.2021.114462 34324951

[B2] Alfwuaires M. A. Algefare A. I. Afkar E. Salam S. A. El-Moaty H. I. A. Badr G. M. (2021). Immunomodulatory assessment of Portulaca oleracea L. extract in a mouse model of colitis. Biomed. Pharmacother. 143, 112148. 10.1016/j.biopha.2021.112148 34560553

[B3] Alharbi T. S. Alshammari Z. S. Alanzi Z. N. Althobaiti F. Elewa M. a. F. Hashem K. S. (2024). Therapeutic effects of genistein in experimentally induced ulcerative colitis in rats *via* affecting mitochondrial biogenesis. Mol. Cell. Biochem. 479 (2), 431–444. 10.1007/s11010-023-04746-8 37084167

[B4] Almarzooqi S. Venkataraman B. Raj V. Alkuwaiti S. a. A. Das K. M. Collin P. D. (2022). β-Myrcene mitigates Colon inflammation by inhibiting MAP kinase and NF-κB signaling pathways. Molecules 27 (24), 8744. 10.3390/molecules27248744 PMC9782154 36557879

[B5] Alqudah A. Qnais E. Gammoh O. Bseiso Y. Wedyan M. Alqudah M. (2025). Exploring scopoletin's therapeutic efficacy in DSS-induced ulcerative colitis: insights into inflammatory pathways, immune modulation, and microbial dynamics. Inflammation 48 (2), 575–589. 10.1007/s10753-024-02048-9 PMC12053357 38918333

[B6] Araruna M. E. C. Alves Júnior E. B. De Lima Serafim C. A. Pessoa M. M. B. De Souza Pessôa M. L. Alves V. P. (2024). (-)-Fenchone ameliorates TNBS-induced colitis in rats *via* antioxidant, immunomodulatory, and cytoprotective mechanisms. Pharm. (Basel) 18 (1), 18. 10.3390/ph18010018 PMC11769309 39861081

[B7] Bullard B. M. Mcdonald S. J. Cardaci T. D. Vanderveen B. N. Mohammed A. D. Kubinak J. L. (2024). Panaxynol improves crypt and mucosal architecture, suppresses colitis-enriched microbes, and alters the immune response to mitigate colitis. Am. J. Physiol. Gastrointest. Liver Physiol. 326 (5), G591–G606. 10.1152/ajpgi.00004.2024 PMC11376977 38469632

[B8] Cai J. Liu J. Fan P. Dong X. Zhu K. Liu X. (2021). Dioscin prevents DSS-Induced colitis in mice with enhancing intestinal barrier function and reducing colon inflammation. Int. Immunopharmacol. 99, 108015. 10.1016/j.intimp.2021.108015 34339962

[B9] Cai Y. Li S. Yang Y. Duan S. Fan G. Bai J. (2024). Intestinal epithelial damage-derived mtDNA activates STING-IL12 axis in dendritic cells to promote colitis. Theranostics 14 (11), 4393–4410. 10.7150/thno.96184 PMC11303083 39113810

[B10] Cai D. Hu W. Cai Y. Fang T. Chen X. (2025). Integrated analysis of ferroptosis and immune infiltration in ulcerative colitis based on bioinformatics. J. Inflamm. Res. 18, 3535–3549. 10.2147/jir.S501651 PMC11908401 40093944

[B11] Cao R. Wu X. Guo H. Pan X. Huang R. Wang G. (2021). Naringin exhibited therapeutic effects against DSS-induced mice ulcerative colitis in intestinal barrier-dependent manner. Molecules 26 (21), 6604. 10.3390/molecules26216604 PMC8588024 34771012

[B12] Cao J. Qin L. Zhang L. Wang K. Yao M. Qu C. (2024). Protective effect of cellulose and soluble dietary fiber from Saccharina japonica by-products on regulating inflammatory responses, gut microbiota, and SCFAs production in colitis mice. Int. J. Biol. Macromol. 267 (Pt 1), 131214. 10.1016/j.ijbiomac.2024.131214 38580029

[B13] Cao S. Guo X. Xin M. Wang X. Huo J. Yue Y. (2025). Sesamin ameliorates ulcerative colitis by modulating the DUSP1/ERK feedback loop and restoring gut microbiota homeostasis. Phytomedicine 147, 157188. 10.1016/j.phymed.2025.157188 40876127

[B14] Cen L. Yi T. Hao Y. Shi C. Shi X. Lu Y. (2022). Houttuynia cordata polysaccharides alleviate ulcerative colitis by restoring intestinal homeostasis. Chin. J. Nat. Med. 20 (12), 914–924. 10.1016/s1875-5364(22)60220-6 36549805

[B15] Chen B. Li D. Tong B. Wang L. Lin H. Xu H. (2024). Oral alginate microspheres for the efficient site-specific delivery of epidermal growth factor attenuated murine ulcerative colitis *via* repairing the mucosal barrier. Int. J. Pharm. 661, 124394. 10.1016/j.ijpharm.2024.124394 38944169

[B16] Chen C. Lin X. H. Xie Y. M. Xiong S. L. Hou S. Z. Huang S. (2025). Shengjiang Xiexin decoction ameliorates DSS-induced ulcerative colitis *via* activating Wnt/β-Catenin signaling to enhance epithelium renovation and modulating intestinal flora. Phytomedicine 139, 156456. 10.1016/j.phymed.2025.156456 39914063

[B17] Chen M. Ding Y. Tong Z. (2020). Efficacy and safety of Sophora flavescens (Kushen) based traditional Chinese medicine in the treatment of ulcerative colitis: clinical evidence and potential mechanisms. Front. Pharmacol. 11, 603476. 10.3389/fphar.2020.603476 PMC7758483 33362558

[B18] Chen J. Zhong K. Qin S. Jing Y. Liu S. Li D. (2023). Astragalin: a food-origin flavonoid with therapeutic effect for multiple diseases. Front. Pharmacol. 14, 1265960. 10.3389/fphar.2023.1265960 PMC10619670 37920216

[B19] Chen J. Shen B. Jiang Z. (2022). Traditional Chinese medicine prescription Shenling BaiZhu powder to treat ulcerative colitis: clinical evidence and potential mechanisms. Front. Pharmacol. 13, 978558. 10.3389/fphar.2022.978558 PMC9494158 36160392

[B20] Chen J. Zhang H. Fu T. Zhao J. Nowak J. K. Kalla R. (2024). Exposure to air pollution increases susceptibility to ulcerative colitis through epigenetic alterations in CXCR2 and MHC class III region. EBioMedicine 110, 105443. 10.1016/j.ebiom.2024.105443 PMC11605448 39536393

[B21] Chen L. Liu D. Mao M. Liu W. Wang Y. Liang Y. (2022). Betaine ameliorates acute sever ulcerative colitis by inhibiting oxidative stress induced inflammatory pyroptosis. Mol. Nutr. Food Res. 66 (22), e2200341. 10.1002/mnfr.202200341 36069237

[B22] Chen Y. E. Xu S. J. Lu Y. Y. Chen S. X. Du X. H. Hou S. Z. (2021). Asperuloside suppressing oxidative stress and inflammation in DSS-induced chronic colitis and RAW 264.7 macrophages *via* Nrf2/HO-1 and NF-κB pathways. Chem. Biol. Interact. 344, 109512. 10.1016/j.cbi.2021.109512 33974900

[B23] Chen Y. Wang B. Yuan X. Lu Y. Hu J. Gao J. (2021). Vitexin prevents colitis-associated carcinogenesis in mice through regulating macrophage polarization. Phytomedicine 83, 153489. 10.1016/j.phymed.2021.153489 33571919

[B24] Chen Z. Wang H. Tan L. Liu X. (2024). Protective effects of four structurally distinct sanshools ameliorate dextran sodium sulfate-induced ulcerative colitis by restoring intestinal barrier function and modulating the gut microbiota. Antioxidants (Basel) 13 (2), 153. 10.3390/antiox13020153 PMC10886262 38397751

[B25] Chen Z. Huang G. Fang G. Wang W. Lan F. Lin X. (2025). Nobiletin regulates gut microbiota and mediates HIF-1α to alleviate DSS-induced ulcerative colitis by inhibiting ferroptosis. J. Agric. Food Chem. 73 (31), 19427–19441. 10.1021/acs.jafc.5c03076 40686327

[B26] Cheng C. Zhang W. Zhang C. Ji P. Wu X. Sha Z. (2021). Hyperoside ameliorates DSS-induced colitis through MKRN1-Mediated regulation of PPARγ signaling and Th17/Treg balance. J. Agric. Food Chem. 69 (50), 15240–15251. 10.1021/acs.jafc.1c06292 34878764

[B27] Cheng H. Zhang D. Wu J. Liu J. Tan Y. Feng W. (2023). Atractylodes macrocephala Koidz. volatile oil relieves acute ulcerative colitis *via* regulating gut microbiota and gut microbiota metabolism. Front. Immunol. 14, 1127785. 10.3389/fimmu.2023.1127785 PMC10187138 37205093

[B28] Cheng T. Xu C. Wu D. Yan G. Wang C. Wang T. (2023). Sodium houttuyfonate derived from Houttuynia cordata thunb improves intestinal malfunction *via* maintaining gut microflora stability in Candida albicans overgrowth aggravated ulcerative colitis. Food Funct. 14 (2), 1072–1086. 10.1039/d2fo02369e 36594429

[B29] Chi X. Chen T. Luo F. Zhao R. Li Y. Hu S. (2024). Targeted no-releasing L-arginine-induced hesperetin self-assembled nanoparticles for ulcerative colitis intervention. Acta Biomater. 190, 560–578. 10.1016/j.actbio.2024.10.037 39461688

[B30] Choi H. B. Kim R. W. Bae G. S. Jang J. H. Kim Y. S. Park B. O. (2025). Anti-inflammatory effects of amomum villosum extract on dextran sodium sulfate-induced colitis in mice. Curr. Issues Mol. Biol. 47 (6), 389. 10.3390/cimb47060389 PMC12191847 40699788

[B31] Çimen B. Aslan A. Beyaz S. Gok O. Baspinar S. Tan F. (2024). The role of Lycium barbarum (Goji Berry) in apoptosis and autophagy in the experimental ulcerative colitis model. Pharm. Chem. J. 58 (2), 282–289. 10.1007/s11094-024-03144-8

[B32] Deng B. Liao F. Liu Y. He P. Wei S. Liu C. (2023). Comprehensive analysis of endoplasmic reticulum stress-associated genes signature of ulcerative colitis. Front. Immunol. 14, 1158648. 10.3389/fimmu.2023.1158648 PMC10243217 37287987

[B33] Dong W. Xu H. Wei W. Ning R. Chang Y. (2024). Advances in the study of ferroptosis and its relationship to autoimmune diseases. Int. Immunopharmacol. 140, 112819. 10.1016/j.intimp.2024.112819 39096870

[B34] Dou W. Xu H. Feng S. Liu T. Xiao L. Wu Y. (2023). The alleviating effects and mechanisms of betaine on dextran sulfate sodium-induced colitis in mice. Mol. Nutr. Food Res. 67 (24), e2300376. 10.1002/mnfr.202300376 37815169

[B35] Duan R. Wu Y. Wang M. Wu J. Wang X. Wang Z. (2021). Association between short-term exposure to fine particulate pollution and outpatient visits for ulcerative colitis in beijing, China: a time-series study. Ecotoxicol. Environ. Saf. 214, 112116. 10.1016/j.ecoenv.2021.112116 33706140

[B36] Fang Y. Min S. Chen H. Zhu Z. Li Y. Tong Y. (2025). Isoimperatorin, a natural furanocoumarin, ameliorates ulcerative colitis by inducing treg cell generation and promoting mucosal healing. Mol. Med. 31 (1), 275. 10.1186/s10020-025-01334-y 40775284 PMC12330085

[B37] Feng Z. Jiao L. Wu Z. Xu J. Gu P. Xu S. (2021). A novel nanomedicine ameliorates acute inflammatory bowel disease by regulating macrophages and T-Cells. Mol. Pharm. 18 (9), 3484–3495. 10.1021/acs.molpharmaceut.1c00415 34310145

[B38] Fritsch J. Garces L. Quintero M. A. Pignac-Kobinger J. Santander A. M. Fernández I. (2021). Low-fat, high-fiber diet reduces markers of inflammation and dysbiosis and improves quality of life in patients with ulcerative colitis. Clin. Gastroenterol. Hepatol. 19 (6), 1189–1199.e1130. 10.1016/j.cgh.2020.05.026 32445952

[B39] Fu R. Wang L. Meng Y. Xue W. Liang J. Peng Z. (2022). Apigenin remodels the gut microbiota to ameliorate ulcerative colitis. Front. Nutr. 9, 1062961. 10.3389/fnut.2022.1062961 36590200 PMC9800908

[B40] Gao F. Zhu F. Shuai B. Wu M. Wei C. Yuan Y. (2024). Quercetin ameliorates ulcerative colitis by restoring the balance of M2/M1 and repairing the intestinal barrier *via* downregulating cGAS‒STING pathway. Front. Pharmacol. 15, 1351538. 10.3389/fphar.2024.1351538 PMC11106451 38774206

[B41] Ghasemi-Dehnoo M. Amini-Khoei H. Lorigooini Z. Anjomshoa M. Rafieian-Kopaei M. (2023). Ferulic acid ameliorates ulcerative colitis in a rat model *via* the inhibition of two LPS-TLR4-NF-κB and NF-κB-INOS-NO signaling pathways and thus alleviating the inflammatory, oxidative and apoptotic conditions in the colon tissue. Inflammopharmacology 31 (5), 2587–2597. 10.1007/s10787-023-01277-y 37432553

[B42] Gros B. Kaplan G. G. (2023). Ulcerative colitis in adults: a review. Jama 330 (10), 951–965. 10.1001/jama.2023.15389 37698559

[B43] Guo R. Meng Q. Wang B. Li F. (2021). Anti-inflammatory effects of platycodin D on dextran sulfate sodium (DSS) induced colitis and E. coli lipopolysaccharide (LPS) induced inflammation. Int. Immunopharmacol. 94, 107474. 10.1016/j.intimp.2021.107474 33611056

[B44] Guo W. Wang X. Liu F. Chen S. Wang S. Zhang Q. (2022). Acteoside alleviates dextran sulphate sodium-induced ulcerative colitis *via* regulation of the HO-1/HMGB1 signaling pathway. Mol. Med. Rep. 26 (6), 360. 10.3892/mmr.2022.12877 PMC9641715 36281914

[B45] Guo Y. Zhu Y. Zhang J. He Y. Zhao M. Lin H. (2025). Facilitation of mucosal healing by estrogen receptor β in ulcerative colitis through suppression of branched-chain amino acid transport and subsequent triggering of autophagy in colonic epithelial cells. Acta Pharm. Sin. B 15 (1), 168–187. 10.1016/j.apsb.2024.11.014 40041890 PMC11873653

[B46] Herrador-López M. Martín-Masot R. Navas-López V. M. (2023). Dietary interventions in ulcerative colitis: a systematic review of the evidence with meta-analysis. Nutrients 15 (19), 4194. 10.3390/nu15194194 37836478 PMC10574654

[B47] Hu J. Tong Y. Shen Z. Li Y. Cheng C. Au R. (2022). Gegen Qinlian decoction ameliorates murine colitis by inhibiting the expansion of Enterobacteriaceae through activating PPAR-γ signaling. Biomed. Pharmacother. 154, 113571. 10.1016/j.biopha.2022.113571 36007273

[B48] Hu S. Zhao R. Chen T. Chi X. Li Y. Wu D. (2024). Construction of chlorogenic acid nanoparticles for effective alleviation of ulcerative colitis. Food Funct. 15 (18), 9085–9099. 10.1039/d4fo02122c 39157985

[B49] Hu W. Cai Y. Cai D. Chen Z. Huang S. Zhang S. (2025). HIF-1α alleviates ferroptosis in ulcerative colitis by regulation of GPX4. Cell. Death Dis. 16 (1), 542. 10.1038/s41419-025-07883-8 40691131 PMC12280037

[B50] Hu J. Huang H. Che Y. Ding C. Zhang L. Wang Y. (2021). Qingchang Huashi formula attenuates DSS-induced colitis in mice by restoring gut microbiota-metabolism homeostasis and goblet cell function. J. Ethnopharmacol. 266, 113394. 10.1016/j.jep.2020.113394 32941971

[B51] Hu L. H. Liu J. Y. Yin J. B. (2021). Eriodictyol attenuates TNBS-induced ulcerative colitis through repressing TLR4/NF-kB signaling pathway in rats. Kaohsiung J. Med. Sci. 37 (9), 812–818. 10.1002/kjm2.12400 34042266 PMC11896476

[B52] Huang J. R. Wang S. T. Wei M. N. Liu K. Fu J. W. Xing Z. H. (2020). Piperlongumine alleviates mouse colitis and colitis-associated colorectal cancer. Front. Pharmacol. 11, 586885. 10.3389/fphar.2020.586885 33343354 PMC7748110

[B53] Huang J. Wu T. Zhong Y. Huang J. Kang Z. Zhou B. (2023). Effect of curcumin on regulatory B cells in chronic colitis mice involving TLR/MyD88 signaling pathway. Phytother. Res. 37 (2), 731–742. 10.1002/ptr.7656 36196887

[B54] Huang Y. Chen X. Liu X. Lin C. Wang Y. (2024). The coumarin component isofraxidin targets the G-protein-coupled receptor S1PR1 to modulate IL-17 signaling and alleviate ulcerative colitis. Int. Immunopharmacol. 131, 111814. 10.1016/j.intimp.2024.111814 38479159

[B55] Huang H. Yang C. Li S. Zhan H. Tan J. Chen C. (2025). Lizhong decoction alleviates experimental ulcerative colitis *via* regulating gut microbiota-SCFAs-Th17/Treg axis. J. Ethnopharmacol. 349, 119958. 10.1016/j.jep.2025.119958 40350047

[B56] Huang Y. Wang N. Ji X. Luo S. Gong L. Zhao C. (2025). Apigenin ameliorates inflamed ulcerative colitis by regulating mast cell degranulation *via* the PAMP-MRGPRX2 feedback loop. Phytomedicine 140, 156564. 10.1016/j.phymed.2025.156564 40054174

[B57] Ismail Abo El-Fadl H. M. Mohamed M. F. A. (2022). Targeting endoplasmic reticulum stress, Nrf-2/HO-1, and NF-κB by myristicin and its role in attenuation of ulcerative colitis in rats. Life Sci. 311 (Pt B), 121187. 10.1016/j.lfs.2022.121187 36403646

[B58] Ji Z. H. Guo H. X. Wang B. Ren W. Z. Hu J. P. Chen C. (2025a). Apigenin alleviates dextran sulfate sodium-induced ulcerative colitis *via* regulation of endoplasmic reticulum stress in goblet cells. Int. Immunopharmacol. 162, 115164. 10.1016/j.intimp.2025.115164 40614602

[B59] Ji Z. H. Xie W. Y. Wu H. Y. Yuan B. (2025b). Coix seed polysaccharide mitigates ulcerative colitis in mice through the modulation of gut microbiota and improvement of intestinal metabolism balance. J. Agric. Food Chem. 73 (18), 11067–11079. 10.1021/acs.jafc.5c02458 40274530

[B60] Jia Q. Fang S. Yang R. Ling Y. Mehmood S. Ni H. (2024). Genistein alleviates dextran sulfate sodium-induced colitis in mice through modulation of intestinal microbiota and macrophage polarization. Eur. J. Nutr. 63 (5), 1877–1888. 10.1007/s00394-024-03391-1 38592519

[B61] Jia B. Yang W. Li H. Chang G. Zhang X. Zhang N. (2025). Ophiopogonis radix fructan-selenium nanoparticles for dual amelioration of ulcerative colitis and anti-colon cancer. Int. J. Biol. Macromol. 307 (Pt 4), 142327. 10.1016/j.ijbiomac.2025.142327 40118427

[B62] Jiang Y. Song J. Xu Y. Liu C. Qian W. Bai T. (2021). Piezo1 regulates intestinal epithelial function by affecting the tight junction protein claudin-1 *via* the ROCK pathway. Life Sci. 275, 119254. 10.1016/j.lfs.2021.119254 33636174

[B63] Jiang Z. Yan M. Qin Y. Liu Z. Duan Y. Wang Y. (2024). Quercetin alleviates ulcerative colitis through inhibiting CXCL8-CXCR1/2 axis: a network and transcriptome analysis. Front. Pharmacol. 15, 1485255. 10.3389/fphar.2024.1485255 39717557 PMC11663639

[B64] Jiang L. Ma X. Yan Q. Pu D. Fu X. Zhang D. (2025). Dihydromyricetin/montmorillonite intercalation compounds ameliorates DSS-induced colitis: role of intestinal epithelial barrier, NLRP3 inflammasome pathway and gut microbiota. Int. J. Pharm. 670, 125155. 10.1016/j.ijpharm.2024.125155 39746581

[B65] Jiao C. Zhang Q. Yang M. Ma J. Zhao X. Tang N. (2022). Shenling Baizhu San ameliorates ulcerative colitis by regulating the gut microbiota and its tryptophan metabolites: a complementary medicine to mesalamine. J. Ethnopharmacol. 291, 115145. 10.1016/j.jep.2022.115145 35219821

[B66] Jin X. Xia X. Li J. Adu-Frimpong M. Wang X. Wang Q. (2024). Preparation, characterization, pharmacokinetics and ulcerative colitis treatment of hyperoside-loaded mixed micelles. Drug Deliv. Transl. Res. 14 (5), 1370–1388. 10.1007/s13346-023-01470-0 37957475

[B67] Joo M. K. Lee J. W. Woo J. H. Kim H. J. Kim D. H. Choi J. H. (2024). Regulation of colonic neuropeptide Y expression by the gut microbiome in patients with ulcerative colitis and its association with anxiety- and depression-like behavior in mice. Gut Microbes 16 (1), 2319844. 10.1080/19490976.2024.2319844 38404132 PMC10900276

[B68] Jori C. Ahmad A. Kumar A. Kumar B. Ali A. Ali N. (2025). Bioactive chitosan-BSA maillard-derived chrysin-loaded nanoparticles: a gastroprotective, biomucoadhesive approach for enhanced oral therapy in ulcerative colitis. Carbohydr. Polym. 359, 123537. 10.1016/j.carbpol.2025.123537 40306769

[B69] Ju T. Song Z. Qin D. Cheng J. Li T. Hu G. (2024). Neohesperidin attenuates DSS-induced ulcerative colitis by inhibiting inflammation, reducing intestinal barrier damage, and modulating intestinal flora composition. J. Agric. Food Chem. 72 (37), 20419–20431. 10.1021/acs.jafc.4c04433 39249130

[B70] Khan R. Jori C. Ansari M. M. Ahmad A. Nadeem A. Siddiqui N. (2023). α-Terpineol mitigates dextran sulfate sodium-induced colitis in rats by attenuating inflammation and apoptosis. ACS Omega 8 (32), 29794–29802. 10.1021/acsomega.3c04317 37599911 PMC10433518

[B71] Kou R. W. Li Z. Q. Wang J. L. Jiang S. Q. Zhang R. J. He Y. Q. (2024). Ganoderic acid A mitigates inflammatory bowel disease through modulation of AhR activity by microbial tryptophan metabolism. J. Agric. Food Chem. 72 (32), 17912–17923. 10.1021/acs.jafc.4c01166 39078661

[B72] Krugliak Cleveland N. Torres J. Rubin D. T. (2022). What does disease progression look like in ulcerative colitis, and how might it be prevented? Gastroenterology 162 (5), 1396–1408. 10.1053/j.gastro.2022.01.023 35101421

[B73] Kulhari U. Kundu S. Mugale M. N. Sahu B. D. (2023). Nuciferine alleviates intestinal inflammation by inhibiting MAPK/NF-κB and NLRP3/Caspase 1 pathways *in vivo* and *in vitro* . Int. Immunopharmacol. 115, 109613. 10.1016/j.intimp.2022.109613 36577154

[B74] Le Berre C. Honap S. Peyrin-Biroulet L. (2023). Ulcerative colitis. Lancet 402 (10401), 571–584. 10.1016/s0140-6736(23)00966-2 37573077

[B75] Lee H. S. Kim J. M. Lee H. L. Go M. J. Lee D. Y. Kim C. W. (2024). Eucommia ulmoides leaves alleviate cognitive dysfunction in dextran sulfate sodium (DSS)-induced colitis mice through regulating JNK/TLR4 signaling pathway. Int. J. Mol. Sci. 25 (7), 4063. 10.3390/ijms25074063 38612870 PMC11012925

[B76] Leng Y. Zhang X. Zhang Q. Xia J. Zhang Y. Ma C. (2024). Gallic acid attenuates murine ulcerative colitis by promoting group 3 innate lymphocytes, affecting gut microbiota, and bile acid metabolism. J. Nutr. Biochem. 131, 109677. 10.1016/j.jnutbio.2024.109677 38844081

[B77] Li D. Chen J. An J. Gu Y. Dong Z. Gao X. (2025). Sweroside ameliorates intestinal mucosal microcirculatory disturbances in ulcerative colitis mice by modulating the VEGF and hippo signaling pathways. Int. Immunopharmacol. 162, 115079. 10.1016/j.intimp.2025.115079 40836408

[B78] Li X. X. Chen S. G. Yue G. G. Kwok H. F. Lee J. K. Zheng T. (2021). Natural flavone tricin exerted anti-inflammatory activity in macrophage *via* NF-κB pathway and ameliorated acute colitis in mice. Phytomedicine 90, 153625. 10.1016/j.phymed.2021.153625 34256329

[B79] Li C. Zhang W. Wu X. Cai Q. Tan Z. Hong Z. (2022). Aromatic-turmerone ameliorates DSS-induced ulcerative colitis *via* modulating gut microbiota in mice. Inflammopharmacology 30 (4), 1283–1294. 10.1007/s10787-022-01007-w 35794287

[B80] Li W. Wang Y. Zhang Y. Fan Y. Liu J. Zhu K. (2024a). Lizhong decoction ameliorates ulcerative colitis by inhibiting ferroptosis of enterocytes *via* the Nrf2/SLC7A11/GPX4 pathway. J. Ethnopharmacol. 326, 117966. 10.1016/j.jep.2024.117966 38401661

[B81] Li W. Zhang Y. Wang Q. Wang Y. Fan Y. Shang E. (2024b). 6-Gingerol ameliorates ulcerative colitis by inhibiting ferroptosis based on the integrative analysis of plasma metabolomics and network pharmacology. Food Funct. 15 (11), 6054–6067. 10.1039/d4fo00952e 38753306

[B82] Li F. Yang Y. Ge J. Wang C. Chen Z. Li Q. (2024). Multi-omics revealed the mechanisms of Codonopsis pilosula aqueous extract in improving UC through blocking abnormal activation of PI3K/Akt signaling pathway. J. Ethnopharmacol. 319 (Pt 2), 117220. 10.1016/j.jep.2023.117220 37820998

[B83] Li H. Pang B. Nie B. Qu S. Zhang K. Xu J. (2022). Dioscin promotes autophagy by regulating the AMPK-mTOR pathway in ulcerative colitis. Immunopharmacol. Immunotoxicol. 44 (2), 238–246. 10.1080/08923973.2022.2037632 35174751

[B84] Li H. Wu Y. Zhou Q. Wu P. Xing Y. Zhuang Z. (2025). Polysaccharide from steamed Polygonatum sibiricum ameliorates ulcerative colitis by protecting the intestinal mucosal barrier and regulating gut microbiota. Int. J. Biol. Macromol. 301, 140343. 10.1016/j.ijbiomac.2025.140343 39880230

[B85] Li J. Wu H. Zhou J. Jiang R. Zhuo Z. Yang Q. (2024). Ruscogenin attenuates ulcerative colitis in mice by inhibiting Caspase-1-Dependent pyroptosis *via* the TLR4/NF-κB signaling pathway. Biomedicines 12 (5), 989. 10.3390/biomedicines12050989 38790951 PMC11117655

[B86] Li J. Hua L. Hu M. Zhu N. Dong S. Jing X. (2025). The protective effect of naringenin on ulcerative colitis in mice through increasing Nrf2 pathway activity. Acta Biochim. Biophys. Sin. (Shanghai) 57 (7), 1068–1080. 10.3724/abbs.2025026 40078102 PMC12367987

[B87] Li L. Ye Z. Qian H. Chen L. Hu Y. Liu X. (2025). Modified Tou Nong Powder obstructs ulcerative colitis by regulating autophagy and mitochondrial function. J. Ethnopharmacol. 340, 119220. 10.1016/j.jep.2024.119220 39645099

[B88] Li N. Ma P. Li Y. Shang X. Nan X. Shi L. (2023). Gut microbiota-derived 12-ketolithocholic acid suppresses the IL-17A secretion from colonic group 3 innate lymphoid cells to prevent the acute exacerbation of ulcerative colitis. Gut Microbes 15 (2), 2290315. 10.1080/19490976.2023.2290315 38062857 PMC10730201

[B89] Li X. Mo K. Tian G. Zhou J. Gong J. Li L. (2023). Shikimic acid regulates the NF-κB/MAPK signaling pathway and gut microbiota to ameliorate DSS-induced ulcerative colitis. J. Agric. Food Chem. 71 (23), 8906–8914. 10.1021/acs.jafc.3c00283 PMC10273308 37257042

[B90] Li X. Chen Y. Peng X. Zhu Y. Duan W. Ji R. (2024). Anti-inflammation mechanisms of a homogeneous polysaccharide from Phyllanthus emblica L. on DSS induced colitis mice *via* the gut microbiota and metabolites alteration. Food Chem. 459, 140346. 10.1016/j.foodchem.2024.140346 38981378

[B91] Li Y. Wu Y. Liang J. Chen P. Xu S. Wang Y. (2025). “Ligustilide suppresses macrophage-mediated intestinal inflammation and restores gut barrier *via* EGR1-ADAM17-TNF-α pathway in colitis mice,”, 8. Research Wash D C, 0864. 10.34133/research.0864 PMC12404647 40904624

[B92] Li Z. Chu T. Sun X. Zhuang S. Hou D. Zhang Z. (2025). Polyphenols-rich Portulaca oleracea L. (purslane) alleviates ulcerative colitis through restiring the intestinal barrier, gut microbiota and metabolites. Food Chem. 468, 142391. 10.1016/j.foodchem.2024.142391 39675274

[B93] Lin L. Li Q. Yang Y. Zhang C. Wang W. Ni F. (2025). CaGA nanozymes inhibit oxidative stress and protect mitochondrial function in ulcerative colitis therapy. Acta Biomater. 196, 380–398. 10.1016/j.actbio.2025.03.003 40044102

[B94] Liu P. Bian Y. Liu T. Zhong J. Zhong Y. Zhuang S. (2020). Huai hua san alleviates dextran sulphate sodium-induced colitis and modulates colonic microbiota. J. Ethnopharmacol. 259, 112944. 10.1016/j.jep.2020.112944 32387236

[B95] Liu Y. Li B. G. Su Y. H. Zhao R. X. Song P. Li H. (2022). Potential activity of traditional Chinese medicine against ulcerative colitis: a review. J. Ethnopharmacol. 289, 115084. 10.1016/j.jep.2022.115084 35134488

[B96] Liu Y. Dong Y. Shen W. Du J. Sun Q. Yang Y. (2023). Platycodon grandiflorus polysaccharide regulates colonic immunity through mesenteric lymphatic circulation to attenuate ulcerative colitis. Chin. J. Nat. Med. 21 (4), 263–278. 10.1016/s1875-5364(23)60435-2 37120245

[B97] Liu Y. Sun Q. Xu X. Li M. Gao W. Li Y. (2025a). Platycodon grandiflorus polysaccharides combined with hesperidin exerted the synergistic effect of relieving ulcerative colitis in mice by modulating PI3K/AKT and JAK2/STAT3 signaling pathways. Chin. J. Nat. Med. 23 (7), 848–862. 10.1016/s1875-5364(25)60913-7 40653325

[B98] Liu Y. Zhang Z. Li X. Hu Q. Jiang Z. Lv J. (2025b). Wogonin effects on the efflux transporters BCRP and MRP2, explain its effectiveness in ulcerative colitis: implications for metabolic and transport interactions. Pharmacol. Res. 212, 107570. 10.1016/j.phrs.2024.107570 39746499

[B99] Liu J. Yang C. Wu Z. Pei J. Chen Y. Huang X. (2021). Gardenia jasminoides ellis fruit extracts attenuated colitis in 2,4,6-Trinitrobenzenesulfonic acid-induced rats. Evid. Based Complement. Altern. Med. 2021, 9920379–11. 10.1155/2021/9920379 PMC8694976 34956390

[B100] Liu M. Sun H. Fu H. Fu L. Zheng X. Chen Y. (2024). 7-hydroxycoumarin ameliorates ulcerative colitis in mice by inhibiting the MAPK pathway and alleviating gut microbiota dysbiosis. BMC Gastroenterol. 24 (1), 405. 10.1186/s12876-024-03499-y 39533196 PMC11555800

[B101] Liu, T. Y. Han Z. W. Jia S. S. Ma K. Li M. Yi X. (2025). An acidic polysaccharide from Lycium barbarum L: isolation, purification, structural characterization, and therapeutic effects on ulcerative colitis. Int. J. Biol. Macromol. 319 (Pt 4), 145602. 10.1016/j.ijbiomac.2025.145602 40581006

[B102] Liu, W. J. Ma S. B. Li J. X. Fan B. S. Du Y. Xu Z. H. (2025). Explore the key targets and mechanism of danggui buxue decoction against ulcerative colitis: network pharmacology and experimental validation. J. Ethnopharmacol. 344, 119580. 10.1016/j.jep.2025.119580 40043827

[B103] Liu X. Zhang M. Chen S. Liu H. Ma H. Hu T. (2024). Grifola frondosa polysaccharide's therapeutic potential in oxazolone-induced ulcerative colitis. Carbohydr. Polym. 344, 122517. 10.1016/j.carbpol.2024.122517 39218542

[B104] Liu X. F. Qiao J. Gao J. Chen Z. J. Liu X. (2021). Protective effects of total saponins of codonopsis on ulcerative colitis induced by TNBS in rats and its mechanism. Zhongguo Ying Yong Sheng Li Xue Za Zhi 37 (4), 397–401. 10.12047/j.cjap.6051.2021.032 34374260

[B105] Liu Y. Wu J. Tan L. Li Z. Gao P. He S. (2024). (-)-Syringaresinol attenuates ulcerative colitis by improving intestinal epithelial barrier function and inhibiting inflammatory responses. Phytomedicine 124, 155292. 10.1016/j.phymed.2023.155292 38190784

[B106] Lu X. Sun Y. Zhang Z. Sun Z. Wang S. Xu E. (2025). Regulation of pyroptosis by natural products in ulcerative colitis: mechanisms and therapeutic potential. Front. Pharmacol. 16, 1573684. 10.3389/fphar.2025.1573684 40271055 PMC12014637

[B107] Luo M. Luo Y. (2021). Imperatorin relieved ulcerative colitis by regulating the Nrf-2/ARE/HO-1 pathway in rats. Inflammation 44 (2), 558–569. 10.1007/s10753-020-01353-3 33098052

[B108] Luo Y. T. Wu J. Zhu F. Y. Wu J. Q. Wu P. Liu Y. C. (2022). Gancao Xiexin decoction ameliorates ulcerative colitis in mice *via* modulating gut microbiota and metabolites. Drug Des. Devel Ther. 16, 1383–1405. 10.2147/dddt.S352467 35601674 PMC9114650

[B109] Luo R. Liu J. Cheng Q. Shionoya M. Gao C. Wang R. (2024). Oral microsphere formulation of M2 macrophage-mimetic Janus nanomotor for targeted therapy of ulcerative colitis. Sci. Adv. 10 (26), eado6798. 10.1126/sciadv.ado6798 38941458 PMC11212727

[B110] Lv Q. Xing Y. Liu J. Dong D. Liu Y. Qiao H. (2021). Lonicerin targets EZH2 to alleviate ulcerative colitis by autophagy-mediated NLRP3 inflammasome inactivation. Acta Pharm. Sin. B 11 (9), 2880–2899. 10.1016/j.apsb.2021.03.011 34589402 PMC8463273

[B111] Lv H. Jia H. Cai W. Cao R. Xue C. Dong N. (2023). Rehmannia glutinosa polysaccharides attenuates colitis *via* reshaping gut microbiota and short-chain fatty acid production. J. Sci. Food Agric. 103 (8), 3926–3938. 10.1002/jsfa.12326 36347632

[B112] Ma K. Chen M. Liu J. Ge Y. Wang T. Wu D. (2021). Sodium houttuyfonate attenuates dextran sulfate sodium associated colitis precolonized with Candida albicans through inducing β-glucan exposure. J. Leukoc. Biol. 110 (5), 927–937. 10.1002/jlb.4ab0221-324rrrr 33682190

[B113] Ma S. Yang B. Du Y. Lv Y. Liu J. Shi Y. (2023). 1,8-cineole ameliorates colon injury by downregulating macrophage M1 polarization *via* inhibiting the HSP90-NLRP3-SGT1 complex. J. Pharm. Anal. 13 (9), 984–998. 10.1016/j.jpha.2023.07.001 37842654 PMC10568110

[B114] Ma X. Li M. Wang X. Xu H. Jiang L. Wu F. (2024). Dihydromyricetin ameliorates experimental ulcerative colitis by inhibiting neutrophil extracellular traps formation *via* the HIF-1α/VEGFA signaling pathway. Int. Immunopharmacol. 138, 112572. 10.1016/j.intimp.2024.112572 38955027

[B115] Martínez-Vázquez S. E. Corral-Ceballos J. M. Yamamoto-Furusho J. K. (2024). Correlation between the quantity and type of dietary fiber with the activity in Mexican patients with ulcerative colitis (UC). Nutrients 16 (18), 3198. 10.3390/nu16183198 39339798 PMC11435422

[B116] Maslin L. A. Weeks B. R. Carroll R. J. Byrne D. H. Turner N. D. (2022). Chlorogenic acid and quercetin in a diet with fermentable fiber influence multiple processes involved in DSS-induced ulcerative colitis but do not reduce injury. Nutrients 14 (18), 3706. 10.3390/nu14183706 36145086 PMC9501002

[B117] Miao Z. Gu M. Raza F. Zafar H. Huang J. Yang Y. (2024). Isoliquiritin ameliorates ulcerative colitis in rats through caspase 3/HMGB1/TLR4 dependent signaling pathway. Curr. Gene Ther. 24 (1), 73–92. 10.2174/1566523223666230731115236 37526181

[B118] Mo J. Ni J. Zhang M. Xu Y. Li Y. Karim N. (2022). Mulberry anthocyanins ameliorate DSS-induced ulcerative colitis by improving intestinal barrier function and modulating gut microbiota. Antioxidants (Basel) 11 (9), 1674. 10.3390/antiox11091674 36139747 PMC9496020

[B119] Nakase H. Sato N. Mizuno N. Ikawa Y. (2022). The influence of cytokines on the complex pathology of ulcerative colitis. Autoimmun. Rev. 21 (3), 103017. 10.1016/j.autrev.2021.103017 34902606

[B120] Ni J. Zhang L. Feng G. Bao W. Wang Y. Huang Y. (2024). Vanillic acid restores homeostasis of intestinal epithelium in colitis through inhibiting CA9/STIM1-mediated ferroptosis. Pharmacol. Res. 202, 107128. 10.1016/j.phrs.2024.107128 38438089

[B121] Ning K. Duan Y. Tong W. Chen Y. Zhang Q. Xie Q. (2023). Protective effects of different molecular weights of purslane (Portulaca oleracea L.) aqueous extract on DSS-induced ulcerative colitis in mice. Antioxidants (Basel) 12 (7), 1400. 10.3390/antiox12071400 PMC10376347 37507939

[B122] Ning K. Shi C. Chi Y. Y. Zhou Y. F. Zheng W. Duan Y. (2024). Portulaca oleracea L. polysaccharide alleviates dextran sulfate sodium-induced ulcerative colitis by regulating intestinal homeostasis. Int. J. Biol. Macromol. 256 (Pt 2), 128375. 10.1016/j.ijbiomac.2023.128375 38000581

[B123] Niu W. Chen Y. Wang L. Li J. Cui Z. Lv J. (2022). The combination of sodium alginate and chlorogenic acid enhances the therapeutic effect on ulcerative colitis by the regulation of inflammation and the intestinal flora. Food Funct. 13 (20), 10710–10723. 10.1039/d2fo01619b 36173280

[B124] Ogata N. Maeda Y. Misawa M. Takenaka K. Takabayashi K. Iacucci M. (2025). Artificial intelligence-assisted video colonoscopy for disease monitoring of ulcerative colitis: a prospective study. J. Crohns Colitis 19 (1), jjae080. 10.1093/ecco-jcc/jjae080 38828734 PMC11725525

[B125] Ouyang Q. Li X. Liang Y. Liu R. (2024). Sea buckthorn polysaccharide ameliorates colitis. Nutrients 16 (9), 1280. 10.3390/nu16091280 38732527 PMC11085905

[B126] Panossian A. Lemerond T. (2025). Two sides of the same coin for health: Adaptogenic botanicals as nutraceuticals for nutrition and pharmaceuticals in medicine. Pharm. (Basel) 18 (9), 1346. 10.3390/ph18091346 PMC12472958 41011217

[B127] Peng L. Gao X. Nie L. Xie J. Dai T. Shi C. (2020). Astragalin attenuates dextran sulfate sodium (DSS)-induced acute experimental colitis by alleviating gut Microbiota dysbiosis and inhibiting NF-κB activation in mice. Front. Immunol. 11, 2058. 10.3389/fimmu.2020.02058 33042117 PMC7523281

[B128] Peng X. Zhong R. Dong S. Qiu Y. Shu G. Huang S. (2025). 18β-Glycyrrhetinic acid from Glycyrrhiza uralensis protects colonic epithelium in ulcerative colitis by activating Wnt/β-catenin pathway to restore tight junction. Phytomedicine 143, 156830. 10.1016/j.phymed.2025.156830 40450977

[B129] Puppala E. R. Aochenlar S. L. Shantanu P. A. Ahmed S. Jannu A. K. Jala A. (2022). Perillyl alcohol attenuates chronic restraint stress aggravated dextran sulfate sodium-induced ulcerative colitis by modulating TLR4/NF-κB and JAK2/STAT3 signaling pathways. Phytomedicine 106, 154415. 10.1016/j.phymed.2022.154415 36070663

[B130] Qian C. Hu C. Xu Y. Xu W. Wang Z. Gan W. (2025). Intestinal epithelial-derived USP13 alleviates colonic inflammation by suppressing GRP78-mediated endoplasmic reticulum stress. Adv. Sci. (Weinh) 12, e00741. 10.1002/advs.202500741 40679368 PMC12520541

[B131] Qu F. Xu B. Kang H. Wang H. Ji J. Pang L. (2025). The role of macrophage polarization in ulcerative colitis and its treatment. Microb. Pathog. 199, 107227. 10.1016/j.micpath.2024.107227 39675441

[B132] Qu S. L. Chen L. Wen X. S. Zuo J. P. Wang X. Y. Lu Z. J. (2021). Suppression of Th17 cell differentiation *via* sphingosine-1-phosphate receptor 2 by cinnamaldehyde can ameliorate ulcerative colitis. Biomed. Pharmacother. 134, 111116. 10.1016/j.biopha.2020.111116 33341041

[B133] Qu Y. Li X. Xu F. Zhao S. Wu X. Wang Y. (2021). Kaempferol alleviates murine experimental colitis by restoring gut microbiota and inhibiting the LPS-TLR4-NF-κB axis. Front. Immunol. 12, 679897. 10.3389/fimmu.2021.679897 34367139 PMC8339999

[B134] Ren J. Yue B. Wang H. Zhang B. Luo X. Yu Z. (2020). Acacetin ameliorates experimental colitis in mice *via* inhibiting macrophage inflammatory response and regulating the composition of gut microbiota. Front. Physiol. 11, 577237. 10.3389/fphys.2020.577237 33536931 PMC7848181

[B135] Ren D. D. Chen K. C. Li S. S. Zhang Y. T. Li Z. M. Liu S. (2023). Panax quinquefolius polysaccharides ameliorate ulcerative colitis in mice induced by dextran sulfate sodium. Front. Immunol. 14, 1161625. 10.3389/fimmu.2023.1161625 PMC10321667 37415978

[B136] Ru Y. Luo Y. Liu D. Huang Q. Zhou X. Linghu M. (2024). Isorhamnetin alleviates ferroptosis-mediated colitis by activating the NRF2/HO-1 pathway and chelating iron. Int. Immunopharmacol. 135, 112318. 10.1016/j.intimp.2024.112318 38795598

[B137] Salama R. M. Darwish S. F. El Shaffei I. Elmongy N. F. Fahmy N. M. Afifi M. S. (2022). Morus macroura miq. Fruit extract protects against acetic acid-induced ulcerative colitis in rats: novel mechanistic insights on its impact on miRNA-223 and on the TNFα/NFκB/NLRP3 inflammatory axis. Food Chem. Toxicol. 165, 113146. 10.1016/j.fct.2022.113146 35595039

[B138] Shen H. Zhang S. Zhao W. Ren S. Ke X. Gu Q. (2021). Randomised clinical trial: efficacy and safety of qing-chang-hua-shi granules in a multicenter, randomized, and double-blind clinical trial of patients with moderately active ulcerative colitis. Biomed. Pharmacother. 139, 111580. 10.1016/j.biopha.2021.111580 33857914

[B139] Shen J. Zhao Y. Cui W. (2024). Astragalus mongholicus bunge extract improves ulcerative colitis by promoting PLCB2 to inhibit colonic epithelial cell pyroptosis. J. Ethnopharmacol. 334, 118554. 10.1016/j.jep.2024.118554 38992398

[B140] Sheng Q. Li F. Chen G. Li J. Li J. Wang Y. (2021). Ursolic acid regulates intestinal microbiota and inflammatory cell infiltration to prevent ulcerative colitis. J. Immunol. Res. 2021, 6679316–16. 10.1155/2021/6679316 34007853 PMC8111854

[B141] Shi G. Kong J. Wang Y. Xuan Z. Xu F. (2022). Glycyrrhiza uralensis fisch. alleviates dextran sulfate sodium-induced colitis in mice through inhibiting of NF-κB signaling pathways and modulating intestinal microbiota. J. Ethnopharmacol. 298, 115640. 10.1016/j.jep.2022.115640 36030029

[B142] Song X. Wang W. Liu L. Zhao Z. Shen X. Zhou L. (2024). Poria cocos attenuated DSS-induced ulcerative colitis *via* NF-κB signaling pathway and regulating gut microbiota. Molecules 29 (9), 2154. 10.3390/molecules29092154 PMC11085930 38731645

[B143] Song Z. Qiao Z. Liu J. Han L. Chen X. Wang Y. (2025). Sea buckthorn berries alleviate ulcerative colitis *via* regulating gut faecalibaculum rodentium-mediated butyrate biosynthesis. Phytomedicine 139, 156490. 10.1016/j.phymed.2025.156490 40010029

[B144] Sun S. Wang D. Dan L. Fu T. Chen J. Zhang Y. (2024). Dietary anthocyanin intake, genetic risk, and incident ulcerative colitis: a prospective cohort study. Phytother. Res. 38 (12), 5782–5792. 10.1002/ptr.8341 39349990 PMC11634818

[B145] Tan Y. R. Shen S. Y. Li X. Y. Yi P. F. Fu B. D. Peng L. Y. (2024). Mogroside V reduced the excessive endoplasmic reticulum stress and mitigated the ulcerative colitis induced by dextran sulfate sodium in mice. J. Transl. Med. 22 (1), 488. 10.1186/s12967-024-05285-6 38773576 PMC11110204

[B146] Tang S. Liu W. Zhao Q. Li K. Zhu J. Yao W. (2021). Combination of polysaccharides from Astragalus membranaceus and Codonopsis pilosula ameliorated mice colitis and underlying mechanisms. J. Ethnopharmacol. 264, 113280. 10.1016/j.jep.2020.113280 32822821

[B147] Tang S. Zhong W. Li T. Li Y. Song G. (2023). Isochlorogenic acid A alleviates dextran sulfate sodium-induced ulcerative colitis in mice through STAT3/NF-кB pathway. Int. Immunopharmacol. 118, 109989. 10.1016/j.intimp.2023.109989 36958213

[B148] Tang L. Deng J. Shi P. Zou S. Ran H. Yin F. (2025). Rosmarinic acid improves intestinal barrier integrity through PI3K/AKT/Nrf2-mediated regulation of tight junction protein expression. Int. Immunopharmacol. 164, 115380. 10.1016/j.intimp.2025.115380 40834531

[B149] Tang R. Jiang L. Ji Q. Kang P. Liu Y. Miao P. (2025). Resveratrol targeting MDM2/P53/PUMA axis to inhibit colonocyte apoptosis in DSS-induced ulcerative colitis mice. Front. Pharmacol. 16, 1572906. 10.3389/fphar.2025.1572906 40371345 PMC12075554

[B150] Tang X. Wang K. Liu Z. Luo X. Wu M. Ding H. (2025). Functional chitosan/HP-β-CD hydrogel for targeted co-delivery of Rhubarb-derived nanovesicles and kaempferol for alleviating ulcerative colitis. Carbohydr. Polym. 352, 123206. 10.1016/j.carbpol.2024.123206 39843107

[B151] Tratenšek A. Locatelli I. Grabnar I. Drobne D. Vovk T. (2024). Oxidative stress-related biomarkers as promising indicators of inflammatory bowel disease activity: a systematic review and meta-analysis. Redox Biol. 77, 103380. 10.1016/j.redox.2024.103380 39368456 PMC11490685

[B152] Uzzan M. Martin J. C. Mesin L. Livanos A. E. Castro-Dopico T. Huang R. (2022). Ulcerative colitis is characterized by a plasmablast-skewed humoral response associated with disease activity. Nat. Med. 28 (4), 766–779. 10.1038/s41591-022-01680-y PMC9107072 35190725

[B153] Voelker R. (2024). What is ulcerative colitis? Jama 331 (8), 716. 10.1001/jama.2023.23814 38306113

[B154] Wan J. Wang F. Xiao Y. Cheng Y. Zheng S. Jiang Q. (2024). Poria cocos polysaccharide alleviates dextran sulphate sodium-induced ulcerative colitis in mice by modulating intestinal inflammatory responses and microbial dysbiosis. Int. J. Biol. Macromol. 283 (Pt 2), 137450. 10.1016/j.ijbiomac.2024.137450 39522895

[B155] Wan Q. Huang J. Xiao Q. Zhang Z. Zhang Z. Huang L. (2024). Astragalus polysaccharide alleviates ulcerative colitis by regulating the balance of mTh17/mTreg cells through TIGIT/CD155 signaling. Molecules 29 (1), 241. 10.3390/molecules29010241 38202824 PMC10780736

[B156] Wang X. Gao Y. Wang L. Yang D. Bu W. Gou L. (2021). Troxerutin improves dextran Sulfate Sodium-induced ulcerative colitis in mice. J. Agric. Food Chem. 69 (9), 2729–2744. 10.1021/acs.jafc.0c06755 33621077

[B157] Wang X. Huang S. Zhang M. Su Y. Pan Z. Liang J. (2023a). Gegen Qinlian decoction activates AhR/IL-22 to repair intestinal barrier by modulating gut microbiota-related tryptophan metabolism in ulcerative colitis mice. J. Ethnopharmacol. 302 (Pt B), 115919. 10.1016/j.jep.2022.115919 36356716

[B158] Wang X. Shen C. Wang X. Tang J. Wu Z. Huang Y. (2023b). Schisandrin protects against ulcerative colitis by inhibiting the SGK1/NLRP3 signaling pathway and reshaping gut microbiota in mice. Chin. Med. 18 (1), 112. 10.1186/s13020-023-00815-8 PMC10481484 37674245

[B159] Wang J. Zhang C. Qin J. An N. Bai M. Du R. H. (2025). Direct inhibition of the TXNIP-NLRP3-GSDMD pathway reduces pyroptosis in colonocytes and alleviates ulcerative colitis in mice by the small compound PEITC. Acta Pharmacol. Sin. 46 (9), 2436–2449. 10.1038/s41401-025-01549-z 40195510 PMC12373988

[B160] Wang M. Jiang Y. Chen Z. Jiang D. Jiang X. Ye J. (2025). Colon-targeted mucoadhesive PLGA microspheres loaded with ramulus mori alkaloids for enhanced water-soluble drug delivery in ulcerative colitis treatment. Molecules 30 (9), 1878. 10.3390/molecules30091878 PMC12073386 40363686

[B161] Wang T. Liu X. Zhang W. Wang J. Wang T. Yue W. (2024). Traditional Chinese medicine treats ulcerative colitis by regulating gut microbiota, signaling pathway and cytokine: future novel method option for pharmacotherapy. Heliyon 10 (6), e27530. 10.1016/j.heliyon.2024.e27530 38501018 PMC10945194

[B162] Wang X. Xie X. Li Y. Xie X. Huang S. Pan S. (2024). Quercetin ameliorates ulcerative colitis by activating aryl hydrocarbon receptor to improve intestinal barrier integrity. Phytother. Res. 38 (1), 253–264. 10.1002/ptr.8027 37873559

[B163] Wang Y. Zhong S. Yang K. Luo R. Dai L. Zhong W. (2024). β-1,3-d-glucan particles-based “nest” protected co-loaded Rhein and Emodin regulates microbiota and intestinal immunity for ulcerative colitis treatment. Int. J. Biol. Macromol. 260 (Pt 2), 128818. 10.1016/j.ijbiomac.2023.128818 38103669

[B164] Wei T. Wu L. Ji X. Gao Y. Xiao G. (2022). Ursolic acid protects sodium dodecyl sulfate-induced drosophila ulcerative colitis model by inhibiting the JNK signaling. Antioxidants (Basel) 11 (2), 426. 10.3390/antiox11020426 35204308 PMC8869732

[B165] Wei F. H. Xie W. Y. Zhao P. S. Ji Z. H. Gao F. Chen C. Z. (2025). Crataegus pinnatifida polysaccharide alleviates DSS-induced colitis in mice by regulating the intestinal microbiota and enhancing arginine biosynthesis. Phytomedicine 142, 156794. 10.1016/j.phymed.2025.156794 40315641

[B166] Wei Q. Jiang H. Zeng J. Xu J. Zhang H. Xiao E. (2025). Quercetin protected the gut barrier in ulcerative colitis by activating aryl hydrocarbon receptor. Phytomedicine 140, 156633. 10.1016/j.phymed.2025.156633 40088746

[B167] Wen J. Yang Y. Li L. Xie J. Yang J. Zhang F. (2024). Magnoflorine alleviates dextran sulfate sodium-induced ulcerative colitis *via* inhibiting JAK2/STAT3 signaling pathway. Phytother. Res. 38 (9), 4592–4613. 10.1002/ptr.8271 39079890

[B168] Wu X. Chen H. Gao X. Gao H. He Q. Li G. (2022). Natural herbal remedy wumei decoction ameliorates intestinal mucosal inflammation by inhibiting Th1/Th17 cell differentiation and maintaining microbial homeostasis. Inflamm. Bowel Dis. 28 (7), 1061–1071. 10.1093/ibd/izab348 35092428

[B169] Wu X. Zhang Q. Peng L. Tian Z. Gou G. Zuo W. (2024). Colon-targeted piperine-glycyrrhizic acid nanocrystals for ulcerative colitis synergetic therapy *via* macrophage polarization. J. Mater Chem. B 12 (6), 1604–1616. 10.1039/d3tb02312e 38269414

[B170] Wu, S. X. Chen Z. L. Wang X. H. Gu J. (2025). Astragaloside IV alleviates ulcerative colitis progression by inhibiting WDR5-Mediated ENO1 H3K4me3 modification. Kaohsiung J. Med. Sci. 41 (11), e70064. 10.1002/kjm2.70064 40586619 PMC12622470

[B171] Wu Y. Li Y. Ruan Z. Li J. Zhang L. Lu H. (2020). Puerarin rebuilding the mucus layer and regulating mucin-utilizing bacteria to relieve ulcerative colitis. J. Agric. Food Chem. 68 (41), 11402–11411. 10.1021/acs.jafc.0c04119 32864960

[B172] Wu Y. Tan Z. Lan Q. Liu Y. Liu C. He H. (2025). Identification and validation of feature genes related to mitochondrial dysfunction and oxidative stress in ulcerative colitis. J. Inflamm. Res. 18, 5835–5850. 10.2147/jir.S506851 40331161 PMC12050211

[B173] Wu Z. Zeng H. Zhang L. Pu Y. Li S. Yuan Y. (2020). Patchouli alcohol: a natural sesquiterpene against both inflammation and intestinal barrier damage of ulcerative colitis. Inflammation 43 (4), 1423–1435. 10.1007/s10753-020-01219-8 32388657

[B174] Xiao Q. Huang J. Zhu X. Shi M. Chen L. Chen L. (2024a). Formononetin ameliorates dextran sulfate sodium-induced colitis *via* enhancing antioxidant capacity, promoting tight junction protein expression and reshaping M1/M2 macrophage polarization balance. Int. Immunopharmacol. 142 (Pt B), 113174. 10.1016/j.intimp.2024.113174 39288627

[B175] Xiao Q. Luo L. Zhu X. Yan Y. Li S. Chen L. (2024b). Formononetin alleviates ulcerative colitis *via* reshaping the balance of M1/M2 macrophage polarization in a gut microbiota-dependent manner. Phytomedicine 135, 156153. 10.1016/j.phymed.2024.156153 39423480

[B176] Xie X. Li P. Zhao M. Xu B. Zhang G. Wang Q. (2024). Luteolin ameliorates ulcerative colitis in mice *via* reducing the depletion of NCR(+)ILC3 through Notch signaling pathway. Chin. J. Nat. Med. 22 (11), 991–1002. 10.1016/s1875-5364(24)60568-6 39510641

[B177] Xiong X. Cheng Z. Zhou Y. Wu F. Xie L. Lawless L. (2022). HuanglianGanjiang tang alleviates DSS-induced colitis in mice by inhibiting necroptosis through vitamin D receptor. J. Ethnopharmacol. 298, 115655. 10.1016/j.jep.2022.115655 35988837

[B178] Xiong Y. Cheng Z. Zhang Y. Liu T. Wan Z. Xia C. (2025). Ellagic acid alleviates DSS-induced ulcerative colitis by inhibiting ROS/NLRP3 pathway activation and modulating gut microbiota in mice. Eur. J. Nutr. 64 (1), 64. 10.1007/s00394-024-03577-7 39775279

[B179] Xiu M. Li B. He L. Shi Y. Zhang Y. Zhou S. (2025). Caffeic acid protects against ulcerative colitis *via* inhibiting mitochondrial apoptosis and immune overactivation in drosophila. Drug Des. Devel Ther. 19, 2157–2172. 10.2147/dddt.S499284 40145123 PMC11938933

[B180] Xu Y. Zhu B. W. Sun R. Li X. Wu D. Hu J. N. (2023). Colon-targeting Angelica sinensis polysaccharide nanoparticles with dual responsiveness for alleviation of ulcerative colitis. ACS Appl. Mater Interfaces 15 (22), 26298–26315. 10.1021/acsami.3c02128 37233992

[B181] Xu Z. Zhang Q. Ding C. Wen F. Sun F. Liu Y. (2023). Beneficial effects of hordenine on a model of ulcerative colitis. Molecules 28 (6), 2834. 10.3390/molecules28062834 36985809 PMC10054341

[B182] Yan H. Li Y. Li S. Wu D. Xu Y. Hu J. (2023). Phosphatidylserine-functionalized liposomes-in-microgels for delivering genistein to effectively treat ulcerative colitis. J. Mater Chem. B 11 (43), 10404–10417. 10.1039/d3tb00812f 37877170

[B183] Yan S. Yin L. Dong R. (2023). Inhibition of IEC-6 cell proliferation and the mechanism of ulcerative colitis in C57BL/6 mice by dandelion root polysaccharides. Foods 12 (20), 3800. 10.3390/foods12203800 37893693 PMC10606498

[B184] Yan X. Meng L. Zhang X. Deng Z. Gao B. Zhang Y. (2023). Reactive oxygen species-responsive nanocarrier ameliorates murine colitis by intervening colonic innate and adaptive immune responses. Mol. Ther. 31 (5), 1383–1401. 10.1016/j.ymthe.2023.02.017 36855303 PMC10188638

[B185] Yang C. Du Y. Ren D. Yang X. Zhao Y. (2021). Gut microbiota-dependent catabolites of tryptophan play a predominant role in the protective effects of turmeric polysaccharides against DSS-induced ulcerative colitis. Food Funct. 12 (20), 9793–9807. 10.1039/d1fo01468d 34664583

[B186] Yang L. Ma X. Y. Mu K. X. Dai Y. Xia Y. F. Wei Z. F. (2023). Galangin targets HSP90β to alleviate ulcerative colitis by controlling fatty acid synthesis and subsequent NLRP3 inflammasome activation. Mol. Nutr. Food Res. 67 (11), e2200755. 10.1002/mnfr.202200755 37002873

[B187] Yang X. Sun X. Zhou F. Xiao S. Zhong L. Hu S. (2023a). Protocatechuic acid alleviates dextran-sulfate-sodium-induced ulcerative colitis in mice *via* the regulation of intestinal flora and ferroptosis. Molecules 28 (9), 3775. 10.3390/molecules28093775 PMC10180256 37175184

[B188] Yang X. Wang Q. Zhang X. Li L. Cao X. Zhou L. (2023b). Purple yam polyphenol extracts exert anticolitis and anticolitis-associated colorectal cancer effects through inactivation of NF-κB/p65 and STAT3 signaling pathways. J. Agric. Food Chem. 71 (32), 12177–12189. 10.1021/acs.jafc.3c00346 37545095

[B189] Yang Y. Guo L. Wei L. Yu J. Zhu S. Li X. (2024). Da-yuan-yin decoction alleviates ulcerative colitis by inhibiting complement activation, LPS-TLR4/NF-κB signaling pathway and NET formation. J. Ethnopharmacol. 332, 118392. 10.1016/j.jep.2024.118392 38797378

[B190] Yang S. Duan H. Yan Z. Xue C. Niu T. Cheng W. (2025). Luteolin alleviates ulcerative colitis in mice by modulating gut microbiota and plasma metabolism. Nutrients 17 (2), 203. 10.3390/nu17020203 PMC11768085 39861331

[B191] Yao D. Dai W. Dong M. Dai C. Wu S. (2021). MUC2 and related bacterial factors: therapeutic targets for ulcerative colitis. EBioMedicine 74, 103751. 10.1016/j.ebiom.2021.103751 34902790 PMC8671112

[B192] Yao L. Fang J. Zhao J. Yu J. Zhang X. Chen W. (2024). Dendrobium huoshanense in the treatment of ulcerative colitis: network pharmacology and experimental validation. J. Ethnopharmacol. 323, 117729. 10.1016/j.jep.2024.117729 38190953

[B193] Ye Y. Abulizi A. Zhang Y. Lu F. An Y. Ren C. (2025). Ganoderic acid ameliorates ulcerative colitis by improving intestinal barrier function *via* gut microbiota modulation. Int. J. Mol. Sci. 26 (6), 2466. 10.3390/ijms26062466 40141109 PMC11942431

[B194] Ye X. Pi X. Zheng W. Cen Y. Ni J. Xu L. (2022). The methanol extract of Polygonatum odoratum ameliorates colitis by improving intestinal short-chain fatty acids and gas production to regulate microbiota dysbiosis in mice. Front. Nutr. 9, 899421. 10.3389/fnut.2022.899421 35634366 PMC9133717

[B195] Ye Z. Li Y. She Y. Wu M. Hu Y. Qin K. (2022). Renshen baidu powder protects ulcerative colitis *via* inhibiting the PI3K/Akt/NF-κB signaling pathway. Front. Pharmacol. 13, 880589. 10.3389/fphar.2022.880589 PMC9399752 36034874

[B196] Yi F. Chen N. Zhao M. Gu Z. Yuan Y. Tang X. (2025). Dehydrodiisoeugenol targets NOD2 exerting dual effects against colitis and colorectal cancer: a double-edged sword. Mol. Med. 31 (1), 221. 10.1186/s10020-025-01193-7 40468178 PMC12139060

[B197] Yin S. Li L. Chen X. Wang J. Mao Y. Wang J. (2025). Artesunate alleviated murine ulcerative colitis by regulating immune response through inhibiting endoplasmic reticulum stress. Front. Immunol. 16, 1545468. 10.3389/fimmu.2025.1545468 40079012 PMC11897576

[B198] Yin X. Tian L. Liu Q. Zhao H. (2025). Association between pro-inflammatory diet and ulcerative colitis: a systematic review and meta-analysis. Front. Nutr. 12, 1586691. 10.3389/fnut.2025.1586691 40607028 PMC12213411

[B199] Yu S. Qian H. Zhang D. Jiang Z. (2023). Ferulic acid relieved ulcerative colitis by inhibiting the TXNIP/NLRP3 pathway in rats. Cell. Biol. Int. 47 (2), 417–427. 10.1002/cbin.11935 36251276

[B200] Yu T. Y. Feng Y. M. Kong W. S. Li S. N. Sun X. J. Zhou G. (2023). Gallic acid ameliorates dextran sulfate sodium-induced ulcerative colitis in mice *via* inhibiting NLRP3 inflammasome. Front. Pharmacol. 14, 1095721. 10.3389/fphar.2023.1095721 36762118 PMC9905138

[B201] Yuan H. Zhang T. Huang S. Zhou J. Park S. (2020). Six gentlemen decoction adding Aucklandia and Amomum (Xiangsha Liujunzi Tang) for the treatment of ulcerative colitis: a systematic review and meta-analysis of randomized clinical trials. Eur. J. Integr. Med. 36, 101119. 10.1016/j.eujim.2020.101119

[B202] Yuan X. Chen B. Duan Z. Xia Z. Ding Y. Chen T. (2021). Depression and anxiety in patients with active ulcerative colitis: crosstalk of gut microbiota, metabolomics and proteomics. Gut Microbes 13 (1), 1987779. 10.1080/19490976.2021.1987779 34806521 PMC8632339

[B203] Yuan J. Su J. Zhong S. Yuan X. Zhu J. Lu S. (2025). Dictamnine alleviates DSS-induced colitis mice by inhibiting ferroptosis of enterocytes *via* activating Nrf2-Gpx4 signaling pathway. Eur. J. Pharmacol. 997, 177464. 10.1016/j.ejphar.2025.177464 40049578

[B204] Zang Z. Li L. Yang M. Zhang H. Naeem A. Wu Z. (2024). Study on the ameliorative effect of honeysuckle on DSS-induced ulcerative colitis in mice. J. Ethnopharmacol. 325, 117776. 10.1016/j.jep.2024.117776 38307354

[B205] Zeng X. Tang S. Dong X. Dong M. Shao R. Liu R. (2024). Analysis of metagenome and metabolome disclosed the mechanisms of Dendrobium officinale polysaccharide on DSS-induced ulcerative colitis-affected mice. Int. J. Biol. Macromol. 277 (Pt 2), 134229. 10.1016/j.ijbiomac.2024.134229 39089548

[B206] Zhang D. Duan S. He Z. Zhu Z. Li Z. Yi Q. (2023). Sijunzi decoction targets IL1B and TNF to reduce neutrophil extracellular traps (NETs) in ulcerative colitis: evidence from silicon prediction and experiment validation. Drug Des. Devel Ther. 17, 3103–3128. 10.2147/dddt.S428814 PMC10590142 37868820

[B207] Zhang D. Wu J. Feng H. Tang P. Zhou Y. Zhao C. (2025). Gastrodin ameliorates ulcerative colitis *via* modulating gut microbial tryptophan metabolism and AhR/NLRP3 pathway. Phytomedicine 147, 157217. 10.1016/j.phymed.2025.157217 40907403

[B208] Zhang J. Liang F. Chen Z. Chen Y. Yuan J. Xiong Q. (2022). Vitexin protects against dextran sodium sulfate-induced colitis in mice and its potential mechanisms. J. Agric. Food Chem. 70 (38), 12041–12054. 10.1021/acs.jafc.2c05177 36124900

[B209] Zhang C. Shu Y. Li Y. Wang F. Gan J. Wang Y. (2025). Chinese yam (Dioscorea) polysaccharide ameliorates ulcerative colitis in mice *via* modulating disorders of intestinal microecology and metabolism. Int. J. Biol. Macromol. 315 (Pt 2), 144110. 10.1016/j.ijbiomac.2025.144110 40360104

[B210] Zhang H. Kalla R. Chen J. Zhao J. Zhou X. Adams A. (2024). Altered DNA methylation within DNMT3A, AHRR, LTA/TNF loci mediates the effect of smoking on inflammatory bowel disease. Nat. Commun. 15 (1), 595. 10.1038/s41467-024-44841-y 38238335 PMC10796384

[B211] Zhang H. Wu Y. Li S. Wang D. Zheng M. Yang N. (2025). Protective effects of pachymic acid in a DSS-Induced mouse model of ulcerative colitis. Immunol. Invest. 54 (8), 1386–1398. 10.1080/08820139.2025.2536320 40704782

[B212] Zhang J. Li Z. Zhang Y. Guo Y. L. Zhu Y. R. Xia W. X. (2023). Mume Fructus (Prunus mume Sieb. et Zucc.) extract accelerates colonic mucosal healing of mice with colitis induced by dextran sulfate sodium through potentiation of cPLA2-mediated lysophosphatidylcholine synthesis. Phytomedicine 119, 154985. 10.1016/j.phymed.2023.154985 37516090

[B213] Zhang S. Ren Y. Li M. Gao X. Zhang X. Xu W. (2025). Chebulagic acid ameliorates DSS-induced colitis in mice by improving oxidative stress, inflammation and the gut microbiota. Am. J. Transl. Res. 17 (6), 4101–4118. 10.62347/qwcq2468 40672593 PMC12261200

[B214] Zhang T. Guo Z. Cheng X. Xia R. Lai S. Liao L. (2024). Protective properties of ophiopogonin D in DSS-induced colitis: insights into microbiota modulation. Inflammopharmacology 32 (5), 3553–3570. 10.1007/s10787-024-01531-x 39039348

[B215] Zhang X. Zhang F. Li Y. Fan N. Zhao K. Zhang A. (2024). Blockade of PI3K/AKT signaling pathway by Astragaloside IV attenuates ulcerative colitis *via* improving the intestinal epithelial barrier. J. Transl. Med. 22 (1), 406. 10.1186/s12967-024-05168-w 38689349 PMC11061986

[B216] Zhang X. Cai J. Chen L. Tai J. Liu J. Cao Y. (2025). Gastrodin ameliorates DSS-induced ulcerative colitis *via* enhancing the abundance of Akkermansia muciniphila and modulating the metabolites. Phytomedicine 146, 157138. 10.1016/j.phymed.2025.157138 40779823

[B217] Zhang Y. Ji W. Qin H. Chen Z. Zhou Y. Zhou Z. (2025). Astragalus polysaccharides alleviate DSS-induced ulcerative colitis in mice by restoring SCFA production and regulating Th17/Treg cell homeostasis in a microbiota-dependent manner. Carbohydr. Polym. 349 (Pt A), 122829. 10.1016/j.carbpol.2024.122829 39643403

[B218] Zhang Z. Huang J. Zhu X. Deng B. Zhao H. Wang H. (2025). Ginsenoside Rg1 alleviated experimental colitis in obesity mice by regulating memory follicular T cells *via* Bcl-6/Blimp-1 pathway. J. Nutr. Biochem. 140, 109880. 10.1016/j.jnutbio.2025.109880 40021065

[B219] Zhao Y. Luan H. Jiang H. Xu Y. Wu X. Zhang Y. (2021). Gegen Qinlian decoction relieved DSS-induced ulcerative colitis in mice by modulating Th17/Treg cell homeostasis *via* suppressing IL-6/JAK2/STAT3 signaling. Phytomedicine 84, 153519. 10.1016/j.phymed.2021.153519 33640781

[B220] Zhao J. Wu R. Wei P. Ma Z. Pei H. Hu J. (2023). Ethanol extract of Piper wallichii ameliorates DSS-induced ulcerative colitis in mice: Involvement of TLR4/NF-κB/COX-2 signaling pathway. J. Ethnopharmacol. 308, 116293. 10.1016/j.jep.2023.116293 36806346

[B221] Zhao Y. Ma Y. Pei J. Zhao X. Jiang Y. Liu Q. (2024). Exploring pyroptosis-related signature genes and potential drugs in ulcerative colitis by transcriptome data and animal experimental validation. Inflammation 47 (6), 2057–2076. 10.1007/s10753-024-02025-2 38656456

[B222] Zhao H. Fu X. Wang Y. Shang Z. Li B. Zhou L. (2025). Therapeutic potential of vanillic acid in ulcerative colitis through microbiota and macrophage modulation. Mol. Nutr. Food Res. 69 (4), e202400785. 10.1002/mnfr.202400785 39812000

[B223] Zheng S. Zhuang T. Tang Y. Wu R. Xu T. Leng T. (2021). Leonurine protects against ulcerative colitis by alleviating inflammation and modulating intestinal microflora in mouse models. Exp. Ther. Med. 22 (5), 1199. 10.3892/etm.2021.10633 34584544 PMC8422400

[B224] Zheng J. Zhang J. Zhou Y. Zhang D. Guo H. Li B. (2024). Taurine alleviates experimental colitis by enhancing intestinal barrier function and inhibiting inflammatory response through TLR4/NF-κB signaling. J. Agric. Food Chem. 72 (21), 12119–12129. 10.1021/acs.jafc.4c00662 38761152

[B225] Zheng B. X. Yi Y. Wang X. W. Li C. Y. Zhao Y. Tian J. Z. (2025). Geniposide *via* enema alleviates colitis by modulating intestinal flora and bile acid metabolites, inhibiting S100A8/S100A9/NF-κB, and promoting TGR5 inhibition of NLRP3 inflammasome. Phytomedicine 142, 156791. 10.1016/j.phymed.2025.156791 40279965

[B226] Zhong Y. Xiao Q. Huang J. Yu S. Chen L. Wan Q. (2023). Ginsenoside Rg1 alleviates ulcerative colitis in Obese mice by regulating the gut microbiota-lipid Metabolism-Th1/Th2/Th17 cells axis. J. Agric. Food Chem. 71 (50), 20073–20091. 10.1021/acs.jafc.3c04811 38064669

[B227] Zhou Q. Yu R. Liu T. Li Y. Zhong J. Zhang T. (2021). Coix seed diet ameliorates immune function disorders in experimental colitis mice. Nutrients 14 (1), 123. 10.3390/nu14010123 35010997 PMC8746960

[B228] Zhou Y. Dou F. Song H. Liu T. (2022). Anti-ulcerative effects of wogonin on ulcerative colitis induced by dextran sulfate sodium *via* Nrf2/TLR4/NF-κB signaling pathway in BALB/c mice. Environ. Toxicol. 37 (4), 954–963. 10.1002/tox.23457 35044701

[B229] Zhou C. Peng B. Zhang M. Yang Y. Yi Z. Wu Y. (2024). Ganjiang huangqin Huanglian Renshen decoction protects against ulcerative colitis by modulating inflammation, oxidative stress, and gut microbiota. Phytomedicine 135, 156172. 10.1016/j.phymed.2024.156172 39471735

[B230] Zhou Y. Xiong X. Cheng Z. Chen Z. Wu S. Yu Y. (2024). Ginsenoside Rb1 alleviates DSS-induced ulcerative colitis by protecting the intestinal barrier through the signal network of VDR, PPARγ and NF-κB. Drug Des. Devel Ther. 18, 4825–4838. 10.2147/dddt.S481769 39494151 PMC11531243

[B231] Zhou Z. J. Dong J. Y. Qiu Y. Zhang G. L. Wei K. He L. H. (2024). Sulforaphane decreases oxidative stress and inhibits NLRP3 inflammasome activation in a mouse model of ulcerative colitis. Biomed. Pharmacother. 175, 116706. 10.1016/j.biopha.2024.116706 38713944

[B232] Zhou J. Yang Q. Wei W. Huo J. Wang W. (2025). Codonopsis pilosula polysaccharide alleviates ulcerative colitis by modulating gut microbiota and SCFA/GPR/NLRP3 pathway. J. Ethnopharmacol. 337 (Pt 2), 118928. 10.1016/j.jep.2024.118928 39393557

[B233] Zhu Y. Zhao Q. Huang Q. Li Y. Yu J. Zhang R. (2022). Nuciferine regulates immune function and gut microbiota in DSS-induced ulcerative colitis. Front. Vet. Sci. 9, 939377. 10.3389/fvets.2022.939377 35909691 PMC9328756

[B234] Zhuang H. Lv Q. Zhong C. Cui Y. He L. Zhang C. (2021). Tiliroside ameliorates ulcerative colitis by restoring the M1/M2 macrophage balance *via* the HIF-1α/glycolysis pathway. Front. Immunol. 12, 649463. 10.3389/fimmu.2021.649463 33868286 PMC8044352

[B235] Zhuang B. Zhuang J. Wang Y. Zeng J. (2025). Betulinic acid-loaded carboxymethyl chitosan/sodium alginate microspheres ameliorate dextran sodium sulfate-induced colitis and anxiety-like behaviors in mice. J. Bioact. Compatible Polym. 40 (4), 355–370. 10.1177/08839115251356266

[B236] Zhuge X. Jin X. Ji T. Li R. Xue L. Yu W. (2023). Geniposide ameliorates dextran sulfate sodium-induced ulcerative colitis *via* KEAP1-Nrf2 signaling pathway. J. Ethnopharmacol. 314, 116626. 10.1016/j.jep.2023.116626 37187359

[B237] Zou Y. Ding W. Wu Y. Chen T. Ruan Z. (2023). Puerarin alleviates inflammation and pathological damage in colitis mice by regulating metabolism and gut microbiota. Front. Microbiol. 14, 1279029. 10.3389/fmicb.2023.1279029 37908541 PMC10614640

