# Supplementary material for: Bioactive metabolites and botanical preparations from plants used as medicines and foods in ulcerative colitis: a mechanism-oriented narrative review of multi-target regulation
Source: Front Pharmacol. 2026 Oct 30;17:1915609. doi: 10.3389/fphar.2026.1915609 (PMC13760205; doi:10.3389/fphar.2026.1915609)
Supplement: Supplementary file 1 [file DataSheet1.pdf]

# Supplementary evidence tables for ulcerative colitis

These tables summarize reported botanical preparations, metabolites, and combined interventions. We retain study-specific regimens and quantitative findings where verified; different preparations or studies of the same plant are separate evidence entries.

Reading the tables: UV means unverified or unavailable in the materials reviewed, not necessarily absent from the original article. Values and units are retained as reported; percentages calculated from group means are descriptive changes, not pooled or standardized effect sizes. A stated significance threshold is not an endpoint-specific P value. Arrows indicate reported direction and do not by themselves establish significance or causality. Sample size refers to the stated allocation or assay, not necessarily every experiment.

Names follow Plants of the World Online, fungal names were checked separately in Species Fungorum. Unresolved source species are not inferred from common names. Unless explicitly clinical, findings are preclinical and do not establish human efficacy, safety or achievable exposure.

Abbreviations: ABX, antibiotic treatment; DAI, disease activity index; DSS, dextran sulfate sodium; FMT, fecal microbiota transplantation; i.p., intraperitoneal; PK, pharmacokinetics; SASP, sulfasalazine; SCFA, short-chain fatty acid; SD, standard deviation; SEM, standard error of the mean; TNBS, trinitrobenzene sulfonic acid; UC, ulcerative colitis; 5-ASA, mesalamine; ↑/↓, increased/decreased.

## Table S1 Botanical formulations

| Material and reference | Natural source and reported system | Reported observations | Design and quantitative information | Verification and limitation |
|---|---|---|---|---|
| Huanglian-Ganjiang Tang (HGT) (Xiong et al., 2022) | *Coptis chinensis* Franch., *Zingiber officinale* Rosc., *Panax ginseng* C.A.Mey., *Ziziphus jujuba* Mill., *Glycyrrhiza uralensis* Fisch.; male C57BL/6 mice; 3% DSS colitis. | DAI↓, histological injury↓, inflammatory mRNA↓; high-dose VDR/E-cadherin/Occludin↑; p-RIPK1/p-RIPK3/p-MLKL↓, Caspase-8↑. | Gavage: 1.7/3.4 g crude-drug equivalents/kg/day (calculated), 10 days from DSS onset; saline controls, no positive drug. Initial n=7 normal, 9/treatment group; disease outcomes n=7–9. Colon length↑ (low $P<0.05$; high $P<0.01$); histology↓ (both $P<0.001$) versus DSS. ANOVA/Tukey or t-test; exact effect sizes unavailable. | Recipe proportions and SD/SEM reporting conflict; molecular n unclear. No VDR necessity test; preliminary higher concentration caused diarrhea. |
| Lizhong decoction (LZD) (Li et al., 2024) | *Panax ginseng* C.A.Mey., *Zingiber officinale* Rosc., *Atractylodes macrocephala* Koidz., *Glycyrrhiza uralensis* Fisch; male C57BL/6J mice, 3% DSS; RSL3-injured Caco-2 cells. | DAI/histological injury/iron/MDA↓; colon length and Nrf2/SLC7A11/GPX4-associated readouts↑, mainly at high dose. | Gavage 1.82/5.46 g/kg/day for 10 days, starting before DSS; n=8/group. Controls: normal, DSS, SASP 0.45 g/kg/day, and Fer-1 1 mg/kg/day. Cells: 1/100 μg/mL, 24 h; RSL3 15 μM; ML385 5 μM in separate assays. High-dose DAI/colon length/histology $P<0.01$ versus DSS; exact means unavailable. SEM; one-way ANOVA. | Cell biological n and dose-mass basis unclear. ML385 panel lacks LZD-alone comparator; Nrf2 necessity not established. |
| Lizhong decoction (LZD) (Huang et al., 2025) | *Panax ginseng* C.A.Mey., *Zingiber officinale* Rosc., *Atractylodes macrocephala* Koidz., *Glycyrrhiza uralensis* Fisch.; 3% DSS, antibiotic-depletion and fecal-transfer experiments. | Colitis↓; Th17/Treg-related responses and SCFA profiles altered; FFAR2/Claudin-1↑, HDAC6↓; benefit lost after antibiotics and transferable by FMT. | Gavage 1.37/2.73/5.46 g/kg/day for 10 days from DSS onset; normal/DSS and SASP 0.45 g/kg/day controls; n=6/group. Antibiotic arm: 5.46 g/kg/day, n=5/group; FMT recipients: n=5/group. Colon length $P<0.05/<0.05/<0.01$ across doses versus DSS; FMT DAI/histology $P<0.01$ versus DSS-donor FMT. ANOVA; exact means unavailable. | SD/SEM and antibiotic-arm colon-length reporting conflict. FMT supports microbial contribution, not necessity of individual taxa/metabolites. |
| Ganjiang-Huangqin-Huanglian-Renshen Decoction (Zhou et al., 2024) | *Zingiber officinale* Rosc., *Scutellaria baicalensis* Georgi, *Coptis chinensis* Franch. *Panax ginseng* C.A.Mey; male C57BL/6J mice; 3% DSS, reported for 21 days. | DAI/inflammation/oxidative injury↓; active caspase-1↓; LC3-II/I, PINK1/Parkin/Beclin-1↑, p62↓; microbiota composition altered. | Oral 4/8/16 g/kg/day on days 8–21; 5-ASA 100 mg/kg/day; high-dose+3-MA 15 mg/kg/day arm; normal/model controls. Analyzed n=6. High-dose SOD/CAT↑ and MDA/NO/ROS↓, $P<0.001$ versus model. SD; ANOVA/LSD; exact means unavailable. | Initial allocation/attrition inconsistent. No 3-MA-only arm; static markers do not establish mitophagy flux or selective dependence. |
| Shenling Baizhu San (SBS) plus mesalamine (Jiao et al., 2022) | *Panax ginseng* C.A.Mey., *Atractylodes macrocephala* Koidz., *Poria cocos* (Schw.) Wolf, *Dioscorea polystachya* Turcz., *Lablab purpureus* (L.) Sweet, *Nelumbo nucifera* Gaertn., *Coix lacryma-jobi* L. var. *ma-yuen* (Roman.) Stapf, *Amomum villosum* Lour., *Platycodon grandiflorus* (Jacq.) A.DC., *Glycyrrhiza uralensis* Fisch. Human | Endoscopic/histological activity↓; IAA/IPA↑ within combination arm. Between-group CRP/TNF-α changes were nonsignificant. | 48 patients, 24/arm; mesalamine 4 g/day±SBS 6 g orally three times/day, 8 weeks. Microbiota n=5/group. UCEIS decrease: combination 5.13±0.88 versus 3.50±1.94 (SD), $P<0.01$; calculated mean difference 1.63 points. Nancy Index decrease: 1.88±0.60 versus 1.33±0.70, $P<0.05$. t-test/ANOVA-Bonferroni. | Nonrandomized cohort; no SBS-only arm. Baseline Mayo values conflict; safety and microbial mediation remain uncertain. |

| Material and reference | Natural source and reported system | Reported observations | Design and quantitative information | Verification and limitation |
|---|---|---|---|---|
| Sijunzi Decoction (SJZ) (Zhang et al., 2023) | *Panax ginseng* C.A.Mey., *Atractylodes macrocephala* Koidz., *Poria cocos* (Schw.) Wolf, *Glycyrrhiza uralensis* Fisch.; male C57BL/6 mice, 3% DSS; differentiated HL-60 NET model. | Colon length↑, spleen index↓; inflammatory and NET-associated IL1B/TNF/NE signals↓. | Gavage 400/800 mg/kg extract; day-7 endpoint; frequency unspecified. Mesalazine 300 mg/kg; normal/model controls. Initial n=10 normal, 14/other group; qPCR n=3, ELISA n=5, cell IF n=3. Cells: PMA 50 nM/4 h; medicated-serum exposure incompletely specified. Colon length P<0.01 and spleen index P<0.05 versus model. SD; t-test/ANOVA or rank-sum. | Cell serum fraction/duration unclear. Donor dosing differs from efficacy regimen; docking/NET markers do not establish direct target necessity. |
| Renshen Baidu powder (RSBDP) (Ye et al., 2022) | *Panax ginseng* C.A.Mey., *Atractylodes macrocephala* Koidz., *Poria cocos* (Schw.) Wolf, *Glycyrrhiza uralensis* Fisch., *Bupleurum chinense* DC., *Peucedanum praeruptorum* Dunn, *Ligusticum chuanxiong* Hort., *Citrus aurantium* L. var. *amara* Engl., *Notopterygium incisum* Ting ex H.T.Chang, *Angelica pubescens* Maxim. f. *biserrate* Shan et Yuan（, *Platycodon grandiflorus* (Jacq.) A.DC., *Mentha haplocalyx* Briq., *Zingiber officinale* Rosc., *Ziziphus jujuba* Mill.; male SD rats; TNBS colitis. | Colitis and serum TNF-α/ICAM-1/endotoxin/DAO↓; phosphorylated PI3K/Akt/NF-κB↓. | 4.93/9.87/19.74 g crude drug/kg/day; SASP 360 mg/kg/day; saline controls. Diagram shows pretreatment before TNBS and dosing through day 22; text states 7-day cycle; formula route unspecified. Disease/ELISA n=6. Endpoint-dependent P<0.05/P<0.01 versus model; exact means unavailable. SEM; one-way ANOVA. | Total allocation and dosing duration inconsistent. Includes pretreatment; phosphorylation changes do not prove pathway necessity. |
| Gegen Qinlian decoction (GD) (Hu et al., 2022) | *Pueraria lobata* (Willd.) Ohwi, *Scutellaria baicalensis* Georgi, *Coptis chinensis* Franch., *Glycyrrhiza uralensis* Fisch.; male C57BL/6J mice, 2.5% DSS; NCM460 cells. | Colitis↓; PPAR-γ activity↑; iNOS/nitrate and Enterobacteriaceae/E. coli↓; T0070907 attenuated protection. | Oral 5.85/17.55 g crude drug/kg, days 8–14 after DSS; n=6–8/group; normal/DSS controls. Mechanism: T0070907 5 mg/kg; reporter cells 2.5/5/25 µg/mL, 24 h, rosiglitazone 10 µM. High-dose colon length 6.05±0.103 versus 5.186±0.1668 cm (SEM), P<0.01; calculated difference +0.864 cm. ANOVA/Tukey. | Corrected microbiota figure used. Cell units and endpoint timing conflict; no antagonist-only arm or direct-binding evidence. |
| Gegen Qinlian decoction (GQ) (Zhao et al., 2021) | *Pueraria lobata* (Willd.) Ohwi, *Scutellaria baicalensis* Georgi, *Coptis chinensis* Franch., *Glycyrrhiza uralensis* Fisch; female C57BL/6 mice; 3% DSS. | Colitis/IL-6/p-JAK2/JAK2/nuclear p-STAT3↓; both DSS-elevated Th17 and Treg proportions↓. | Gavage 1.5/7.5 g/kg/day, days 4–10; n=8/group. Berberine 200 mg/kg/day; normal/model 0.5% CMC-Na controls. Separate flow cohort: 1.5 g/kg/day. Treg decreased in Peyer patches/spleen (P<0.05); Th17 decreased (P<0.001/P<0.05) versus DSS. SD; one-/two-way ANOVA/Tukey. | Dose-mass basis and separate flow-cohort n/timing unclear. Treg expansion and pathway necessity are not demonstrated. |

| Material and reference | Natural source and reported system | Reported observations | Design and quantitative information | Verification and limitation |
|---|---|---|---|---|
| Gegen Qinlian decoction (GQD) (Wang et al., 2023) | *Pueraria lobata* (Willd.) Ohwi, *Scutellaria baicalensis* Georgi, *Coptis chinensis* Franch., *Glycyrrhiza uralensis* Fisch; male C57BL/6 mice; 3% DSS. | Permeability/bacterial translocation↓; barrier integrity/CYP1A1/IL-22+ ILC3/IL-22↑; AhR antagonist attenuated responses. | Oral 2.5/5/7.5 g crude drug/kg during 10-day experiment from DSS onset; frequency unspecified. Normal/model controls, FICZ 1 μg/mouse and CH223191 10 mg/kg+GQD arms. Disease n=6–8; permeability/ILC3 n=6; WB n=3–4. At 5 g/kg, fecal tryptophan↓ ($P<0.001$), IPA/IAA/ILA/indole-3-acetaldehyde↑ ($P<0.05$) versus model. Tukey/Dunnett T3; exact means unavailable. | No antagonist-only or IL-22 loss-of-function arm; full microbial–AhR–IL-22 causal sequence remains unproven. Error convention unclear. |
| Huaihua San (HHS) (Liu et al., 2020) | *Sophora japonica* L., *Carpesium abrotanoides* L., *Artemisia scoparia* Waldst. et Kit., *Citrus aurantium* L; male SD rats; 5% DSS. | DAI/TNF-α/CRP/histological injury↓; vascular leakage↓ at high dose; alpha-diversity recovery nonsignificant. | Gavage 2.88/5.76 g/kg/day, 8 days with pretreatment; n=10/group initially, DAI n=6, histology/permeability n=4. Normal/DSS and HHS-only controls; no positive drug. High-dose DAI and vascular leakage $P<0.05$ versus DSS; low-dose DAI nonsignificant. SD; ANOVA/Student–Newman–Keuls; exact efficacy means unavailable. | Dose/volume and 5.76 versus 5.88 g/kg reporting require clarification. Endpoint subsampling unclear; no causal microbiota experiment. |
| Gancao Xiexin decoction (GCXX) (Luo et al., 2022) | *Glycyrrhiza uralensis* Fisch., *Coptis chinensis* Franch., *Scutellaria baicalensis* Georgi, *Zingiber officinale* Rosc., *Panax ginseng* C.A.Mey., *Pinellia ternate* (Thunb.) Breit., *Ziziphus jujuba* Mill.; male C57BL/6 mice; 3% DSS; antibiotic-treated cohort. | DAI/inflammation/injury↓; acetate increase nonsignificant; detectable benefit lost after antibiotics. | Gavage 8.55 g crude drug/kg/day for 7 days after DSS; mesalazine 200 mg/kg/day and combination arms; normal/UC controls; initial n=10/group. Antibiotic cohort n=6/group. DAI $P<0.01$ versus UC. Colon shortening relative to normal: UC 24.7%, GCXX 10.8% (not a direct treatment-effect estimate). SD or median; ANOVA/LSD or Kruskal–Wallis/Dunn. | Antibiotic-treated mice are not verified germ-free; timing differs between text and diagram. No demonstrated synergy or specific microbial mediator. |
| Danggui Buxue decoction (DBD) (Liu et al., 2025) | *Astragalus membranaceus* (Fisch.) Bunge, *Angelica sinensis* (Oliv.) Diels; male C57BL/6J mice; 3% DSS. | DAI/MPO/inflammation↓; barrier proteins↑; p-PI3K/PI3K and p-AKT/AKT↓. | Gavage 1.25/2.5/5 g/kg, 7 days alongside DSS; frequency unspecified. SASP 75 mg/kg; normal/DSS controls. Disease n=10, ELISA/IF n=6, WB n=4. DAI $P<0.05/<0.01/<0.01$ across doses; colon length $P<0.05/<0.01$ at middle/high doses versus model. SEM; t-test/ANOVA-Bonferroni; exact means unavailable. | Extract/crude-drug dose basis and endpoint day unclear; randomization/blinding not described. No pathway necessity test. |
| Da-yuan-yin decoction (DYY) (Yang et al., 2024) | *Panax ginseng* C.A.Mey., *Rehmannia glutinosa* (Gaertn.) DC., *Angelica sinensis* (Oliv.) Diels, *Ophiopogon japonicus* (L.f.) Ker-Gawl., *Glycyrrhiza uralensis* Fisch.; male C57BL/6 mice, 2.5% DSS; complement-dependent hemolysis assay. | Colitis/complement/TLR4–NF-κB/NET-associated readouts↓; barrier responses improved. | Oral extract 0.4/0.8/1.6 g/kg/day; reported 7–8-day regimen; SSZ 0.3 g/kg, normal/DSS controls. Disease/ELISA n=6; tissue assays generally n=3. DAI $P<0.01/<0.0001/<0.0001$ versus DSS. Hemolysis-inhibition concentration (reported CH50) 0.218±0.033 mg/mL (SD). ANOVA/Bonferroni. | Treatment duration conflicts; hemolysis concentration basis/n unclear. No perturbation establishes the proposed complement–TLR4–NET sequence. |

| Material and reference | Natural source and reported system | Reported observations | Design and quantitative information | Verification and limitation |
| --- | --- | --- | --- | --- |
| Wumei decoction (WMD) (Wu et al., 2022) | Mume fructus, *Zingiber officinale* Rosc., *Coptis chinensis* Franch., *Scutellaria baicalensis* Georgi, *Panax ginseng* C.A.Mey., *Angelica sinensis* (Oliv.) Diels, *Aconitum carmichaelii* Debx., *Zanthoxylum piperitum* DC., *Cinnamomum cassia* Presl., *Glycyrrhiza uralensis* Fisch.; male C57BL/6 chronic-DSS mice and Rag1−/− T-cell-transfer colitis mice. | Colitis/permeability/Th1–Th17-associated responses↓; barrier integrity↑; transfer-model microbiota altered. | Daily gavage 20.48 g/kg; mesalazine 600 mg/kg plus model/normal controls. Chronic DSS: treatment throughout, day-49 table, n=12/group; transfer model: start week 4, day-56 endpoint, n=10/group. Day-49 DAI: WMD 0.72±0.25 versus DSS 1.78±0.34 (SD); calculated difference −1.06 points. $P<0.001$; footnote comparator unclear. Methods: t-tests. | Chronic endpoint: 49/56 days and SD/SEM conflict. Formula identity/dose basis needs clarification; microbial mediation untested. |
| Shengjiang Xiexin decoction (SJXXD) (Chen et al., 2025) | *Zingiber officinale* Rosc., *Glycyrrhiza uralensis* Fisch., *Panax ginseng* C.A.Mey., Zingiber officinale Rosc., *Coptis chinensis* Franch., *Scutellaria baicalensis* Georgi, *Pinellia ternate* (Thunb.) Breit., *Ziziphus jujuba* Mill.; male C57BL/6 mice, 3% DSS; mouse small-intestinal organoids. | Colitis/permeability↓; barrier and β-catenin/c-Myc/Cyclin D1 readouts↑; organoid surface area↑. | Gavage 1.25/5 g raw-drug equivalents/kg during 7-day DSS; mesalazine 100 mg/kg and water controls. Stated n=8/group; five groups listed versus six claimed. Organoids: DSS 0.001%/6 h; SJXXD 5/10/20 µg/mL/24 h; biological n unclear. Colon length $P<0.01$ at both doses; organoid area $P<0.01$ at 10/20 µg/mL. SEM; ANOVA/Tukey–Kramer. | Allocation and organoid dose/sequence unclear; histology blinded. No Wnt blockade or microbial causal test; 5 µg/mL area effect unmarked. |
| Xiangsha Liujunzi Tang (SGDAA) (Yuan et al., 2020) | *Panax ginseng* C.A.Mey., *Atractylodes macrocephala* Koidz., *Poria cocos* (Schw.) Wolf, *Glycyrrhiza uralensis* Fisch., *Citrus reticulata* Blanco, *Pinellia ternate* (Thunb.) Breit., *Aucklandia lappa* Decne., *Amomum villosum* Lour. Human, mice | Pooled curative response favored formula; add-on subgroup nonsignificant; comparative safety uncertain. | 614 participants reported; treatment 4 or 12 weeks; typically one oral prescription/day, some enemas. Comparators included Western treatment and Chinese medicine. Curative response: four trials, n=353; OR 0.52 (95% CI 0.34–0.80), $P=0.003$, $I^2=0\%$; OR defined as control relative to formula. Add-on OR 0.56 (0.27–1.17), $P=0.13$. | Mixed regimens, risk-of-bias concerns and sparse harms reporting. Participant totals/other response CIs conflict; original trials not verified. |

## Table S2 Single resource botanical preparations

| Material and reference | Natural source and reported system | Reported observations | Design and quantitative information | Verification and limitation |
|---|---|---|---|---|
| *Coix lacryma-jobi* var. *ma-yuen* (Rom.Caill.) Stapf (Zhou et al., 2021) | Coix seed feed; female Kunming mice; 5% DSS colitis. | DAI/injury/IL-6/TNF-α/IL-10/MDA↓; RBC/HGB/SOD↑; WBC/LYM↓; $CD4^+/CD3^+$, $CD8^+/CD3^+$ and double-positive/$CD3^+$ ratios↑. | Dietary seed powder replaced corn bran; 10-day pretreatment continued to the endpoint. Four randomized groups, n=8/group; flow n=5; normal/DSS and mesalazine 200 mg/kg controls. Colon length/histology: P<0.01 versus DSS. $CD4^+/CD3^+$: 26.56±1.50% versus 10.37±7.83% (SD), P<0.05. ANOVA. | Seed inclusion/intake insufficient for mg/kg calculation; preventive dietary design with nutritional differences. Mesalazine route unclear; NF-κB not verified. |
| *Portulaca oleracea* L. (Li et al., 2025) | *Portulaca oleracea* 80% ethanol extract; male C57BL/6 mice; 3% DSS; Caco-2/RIMVEC cells. | DAI/CRP/LPS/MPO/iNOS/NLRP3/permeability↓; ZO-1/Occludin/Claudin-1/TER↑; p-p65/p65 and p65 nuclear translocation↓. | Gavage 225/450/900 mg/kg/day for 7 days alongside DSS; n=8/group; normal/DSS and 5-ASA 100 mg/kg controls. Histology n=3, LPS n=7. Cells 0.1–6 mg/mL/24 h; NF-κB assays 0.5/2 mg/mL; permeability n=4, protein n=3. DAI P<0.001 at 450/900 mg/kg; colon length P<0.05 at 900 mg/kg versus DSS. SEM; ANOVA/t-test; exact efficacy means unavailable. | Concurrent-start design; no causal pathway/microbiota test. At 6 mg/mL, cell viability decreased; independent cell n unclear. |
| *Lonicera japonica* Thunb. (Zang et al., 2024) | *Lonicera japonica* flower-bud water decoction; C57BL/6J mice, sex unspecified; 2.5% DSS. | IL-6/TNF-α/MCP-1↓; inflammatory mRNA (COX-2/IL-6/iNOS/NLRP3)↓; CLDN4/MUC2/Occludin mRNA↑. | 0.15/0.75/1.5 g/kg/day throughout a 15-day experiment; normal/model controls, no positive drug; randomized, n=10/group. Route conflicts (mixed feed versus gavage); DSS reported from day 13 for 7 days. IL-6/TNF-α and high-dose MCP-1 P<0.05 versus model; exact means unavailable. SD; ANOVA; two blinded histology assessors. | Route, day numbering, and dose-mass basis unresolved; endpoint n unclear. Histology grades are not group means; microbial findings are associative. |
| *Dendrobium huoshanense* Z.Z.Tang & S.J.Cheng (Yao et al., 2024) | *Dendrobium huoshanense* ultrafine powder suspension; male C57BL/6J mice; 3% DSS. | IL-6/TNF-α↓, IL-10/TGF-β↑; SRC/STAT3/HSP90AA1/PIK3CA↓; p-AKT1/AKT1, p-PI3K/PI3K and p-AKT/AKT↓. | Oral 0.5/1/2 g/kg/day, 21 days; DSS from day 14 for 7 days. Normal/model and mesalazine 300 mg/kg controls. Initial n=12/group; disease/ELISA n=8; histology/WB n=3 per Methods. DAI P<0.001/<0.01 at 1/2 g/kg; colon length both P<0.001 versus DSS. SD; ANOVA/Duncan; exact means unavailable. | Mesalazine timing, histology n, and abstract target direction conflict. Preventive-start design; low-dose DAI/colon effects unmarked; no necessity test. |
| *Lycium barbarum* L. (Cimen et al., 2024) | *Lycium barbarum* warm-water preparation; male Wistar albino rats; 3% DSS. | p53/Beclin-1/GSH/CAT↑; TIGAR and colon/plasma MDA↓; histological injury reduced. | 2% LB drinking water for 4 weeks; 3% DSS in final week; normal, LB-only and DSS controls; animal n unreported. LB+DSS versus DSS: colon MDA 72.34±2.00 versus 82.34±3.00 nmol/g; GSH 446.17±2.00 versus 340.29±1.00 µmol/mg protein (both P<0.05). Calculated changes: −12.14%/+31.11%. ANOVA; ± error type unspecified. | Intake/animal n unknown; triplicate measurements are not biological n. Units/post-hoc methods differ between text, tables and figures; partial protection only. |

## Table S3 Alkaloids and related fractions

| Material and reference | Natural source and reported system | Reported observations | Design and quantitative information | Verification and limitation |
|---|---|---|---|---|
| Ramulus Mori alkaloids and SZ-A-loaded microspheres (Wang et al., 2025) | *Morus alba* L. branch alkaloids in PLGA/HA-SH/alginate microspheres; male C57BL/6J mice; RAW264.7 cells. | ROS/TNF-α/IL-1β/IL-6/DAI↓; Muc-2/ZO-1↑; colon length and histology improved. | Oral microspheres equivalent to SZ-A 25 mg/kg/day through day 10; DSS for the first 5 days. Six randomized groups, 30 mice (5/group by allocation); free SZ-A 25 mg/kg, SASP 300 mg/kg, carrier, and normal/DSS controls. Cells: free drug 5 μg/mL or microspheres 40 μg/mL/24 h after LPS. Colon length/histology $P<0.001$ versus DSS; exact means unavailable. SEM; ANOVA/t-test. | Carrier also active; no direct superiority test versus free drug. Cell biological n unclear; fluorescence retention is not drug PK; safety assessed only briefly. |
| Dictamnine (Yuan et al., 2025) | *Dictamnus dasycarpus* Turcz. (Rutaceae), dried root bark (Dictamni Cortex). Male C57 mice; NCM460 human colon epithelial cells. | DAI, TNF-α, IL-1β, IL-6, $Fe^{2+}$ and MDA↓; occludin, ZO-1, GSH, GPX4 and Nrf2/HO-1/NQO1 readouts↑. | Gavage 10/20/40 mg/kg/day for 10 days; 3% DSS for 7 days; n=6/group. Controls: normal, DSS, and 5-ASA 50 mg/kg. NCM460: dictamnine 10/20 μM with RSL3 1 μM, 24 h; some cellular assays, n=3. BLI/DARTS/CETSA and ML385 experiments supported Nrf2 involvement. Mean ± SD; ANOVA/Tukey, $P<0.05$ threshold; endpoint-specific effect estimates/P values not supplied. | Primary-source check pending. BLI/DARTS/CETSA and ML385 support Nrf2 involvement; exclusive pathway specificity remains unproven. |
| Nuciferine (Zhu et al., 2022; Kulhari et al., 2023) | *Nelumbo nucifera* Gaertn. leaves. Zhu: male BALB/c mice. Kulhari: male BALB/c mice; RAW264.7 cells. | Zhu: colitis↓; $B220^+$/$IgA^+$/$CD3^+$ cells↑; Th1/Th2 and Treg/Th17 balance and microbiota altered. Kulhari: colitis/inflammatory mediators/ROS↓; GSH/SOD/ZO-1/Claudin-1↑; MAPK/NF-κB phosphorylation and NLRP3/caspase-1 readouts↓. | Zhu et al. (2022): Male BALB/c mice; 5% DSS 7 days; nuciferine 10 or 20 mg/kg/day, i.p., day 8–14; n=6/group (microbiota n=4); flow cytometry and 16S rRNA. Kulhari et al. (2023): Male BALB/c mice; 4% DSS for 7 days + 3 days water; nuciferine 20 or 40 mg/kg/day, oral, day 1–10; sulfasalazine 200 mg/kg as positive control; n=6–8/group. RAW 264.7 cells with LPS (0.5 μg/mL) and nuciferine (0.31–1.25 μg/mL). | Zhu: i.p. route, no positive drug control; microbiota n=4. Kulhari: pathway protein readouts; WB n=3. Purity reported as ≥98% and >97%, respectively; regimens are not directly comparable. |
| Hordenine (Xu et al., 2023) | Phenethylamine alkaloid found in *Hordeum vulgare* L. (barley) seedlings, cacti, and other cereals. Female BALB/c mice; mouse colonic epithelial cells (MCECs) co-cultured with peritoneal macrophages. | DAI, weight loss, histological injury and IL-6/IL-1β/TNF-α↓; colon length and ZO-1/occludin↑; SPHK1/S1PR1/Rac1/p-STAT3 readouts↓. MCEC migration↑ in vitro. | 4% DSS for 7 days + 5 days water; groups: control, DSS, DSS+5-ASA (40 mg/kg), DSS+hordenine (50, 25, 12.5 mg/kg), n=10/group; oral gavage days 1–12; sacrifice day 13. MCECs: CCK-8 (500–15.625 μM), 500 μM for co-culture/scratch. DAI, H&E, PAS, ELISA, IHC, WB. | Purity ≥95%; no pathway blockade/rescue. Cellular 500 μM exposure was not related to achieved tissue concentrations. |
| Magnoflorine (Wen et al., 2024) | Magnoflorine occurs in *Phellodendron chinense* C.K.Schneid., *Botrytis cinerea* (S.Moore) L.Lian & Wei Wang, and *Zanthoxylum* spp.; commercial purified compound (≥98%). Male C57BL/6 mice; DSS colitis. | DAI, colon shortening, histological injury and inflammatory cytokines↓; p-JAK2/p-STAT3/c-Myc↓; SOCS3↑. Serum metabolomics suggested altered arachidonic acid metabolism. | 5 groups (n=10): control, DSS, 5-ASA (300 mg/kg), Mag-L (20 mg/kg), Mag-H (40 mg/kg); 3% DSS for 7 days; oral administration once daily for 7 days; sacrifice on day 7. DAI, colon length, spleen index, ELISA (IL-1β, IL-6, IL-16, TNF-α), H&E, Alcian Blue, IHC, Western blot (p-JAK2, p-STAT3, c-Myc, SOCS3), qRT-PCR, serum untargeted metabolomics (UHPLC-Q-TOF/MS). One-way ANOVA, $P<0.05$. | JAK2/STAT3 necessity untested; metabolomics limited to high dose. Systemic exposure was not quantified. |

| Material and reference | Natural source and reported system | Reported observations | Design and quantitative information | Verification and limitation |
|---|---|---|---|---|
| Piperlongumine (Huang et al., 2020) | Alkaloid isolated from *Piper longum* L. (long pepper). Male BALB/c mice (7 weeks old). | Acute DSS: symptoms and colon shortening not improved; inflammatory infiltration and COX-2/IL-6/β-catenin/Snail↓. AOM/DSS: large tumors (>2 mm)↓, without reduced tumor incidence or small tumors. | acute colitis: 2.5% DSS 1 week + water 1 week; PL 10 mg/kg i.p. daily for 2 weeks; n=5/group. AOM/DSS colorectal cancer: AOM 10 mg/kg i.p., then 3 cycles of 2.5% DSS (1 week) + water (2 weeks); PL 10 mg/kg i.p. daily for 9 weeks. H&E, IHC, Western blot (COX-2, IL-6, β-catenin, snail). Student's t-test. | Single i.p. dose, n=5/group; no positive drug control. Molecular changes did not translate into improved acute-colitis symptoms. |
| Hydroxy-α-sanshool (Chen et al., 2024) | Long-chain polyunsaturated amide from the *Zanthoxylum* genus (e.g., *Zanthoxylum bungeanum* Maxim.). Male C57BL/6J mice (20 ± 2 g). | DAI, injury and TNF-α/IL-6↓; colon length, goblet cells and tight-junction proteins↑; p-p65/p-IκB↓. IL-1β unchanged; microbiota composition altered. | Gavage 2.5 mg/kg/day during 3% DSS for 7 days; n=8/group (some assays n=6–8). Controls: normal, DSS, and other sanshools; no standard positive drug. Colon length: 7.56±1.40 versus 5.61±0.37 cm; goblet cells 1.96-fold versus DSS. ANOVA/Duncan; P<0.05 threshold, endpoint-specific P values UV. | Purity ≥98%; single dose, no standard positive drug. Microbial changes are associated. |
| Betaine (Dou et al., 2023; Chen et al., 2022) | Betaine (N, N, N-trimethylglycine), a natural product present in sugar beets and various plants; experimental betaine from Sigma-Aldrich (Dou) / Sigma Chemical Co. (Chen). Male C57BL/6J mice; Dou also used Caco-2 cells. | Dou: colitis/permeability↓; NF-κB–MLCK promoter interaction disrupted; microbiota and cellular tight-junction protein abundance not significantly changed. Chen: colitis/oxidative injury↓; NRF2-associated antioxidant responses↑; NLRP3/caspase-1/GSDMD-related readouts↓. | Dou: male C57BL/6J mice; betaine 20 mg/kg/day oral day 1–28; 3% DSS day 29–35; n=10/group; Caco-2 monolayer with 1 mM betaine + TNF-α/IFN-γ (10 ng/mL). Chen: male C57BL/6J mice; betaine 600 mg/kg/day i.p. during 4% DSS for 1 week; n=7 clinical, n=4–5 assays. DAI, colon length, H&E, FD-4, ELISA, Western blot, immunofluorescence, ChIP, 16S rRNA, TEER. One-way ANOVA or t-test, P<0.05. | Dou: preventive oral regimen; no microbiota change. Chen: i.p. dosing and no NLRP3/GSDMD blockade. Regimens cannot be directly compared. |
| Leonurine (Zheng et al., 2021) | Reported in *Paraleonurus japonicus* (Houtt.) M.L.Qian, Y.P.Chen & C.L.Xiang; C57BL/6J mice, sex unspecified; DSS colitis. | DAI, histological injury and serum TNF-α/IL-6/IL-1β↓; colon length↑; p-p65/p65↓; microbiota composition altered. | C57BL/6J mice (18–20 g, 8 weeks); 3 groups (n=5): control, DSS, DSS+YMJ. Control: water for 15 days. DSS: water for 7 days, then 2.5% DSS for 8 days. DSS+YMJ: 1 mM YMJ in drinking water for 7 days, then 2.5% DSS for 8 days. | Drinking-water concentration lacks intake-based mg/kg exposure. Sex unclear; no positive drug; microbial mediation untested. |

## Table S4 Flavonoids and flavonoid-enriched fractions

| Material and reference | Natural source and reported system | Reported observations | Design and quantitative information | Verification and limitation |
|---|---|---|---|---|
| Puerarin (Wu et al., 2020; Zou et al., 2023) | Isoflavone from *Pueraria montana* var. *lobata* (Willd.) Maesen & S.M.Almeida ex Sanjappa & Predeep root (Radix Puerariae). Wu: Female Sprague-Dawley rats; TNBS-induced colitis. Zou: Male C57BL/6J mice (6–7 weeks, 20–23 g); DSS-induced colitis. | Wu: mucus thickness/MUC2 and SCFAs↑; permeability and inflammation↓. Zou: colitis and IL-1β/IL-6/TNF-α↓; microbiota and tryptophan-related metabolites altered. | Wu: Female SD rats; groups: control, UC (TNBS 100 mg/kg intrarectal), UC+PUE (puerarin 200 mg/kg, oral, 14 days). Mucus thickness, H&E, AB-PAS, RT-qPCR, WB, ELISA, 16S rDNA, SCFAs; n=6–8. Zou: Male C57BL/6J mice; groups: CON, UC, SASP (200 mg/kg), PUE (200 mg/kg); 15% DSS gavage 3×/day for 7 days, then 2 weeks treatment; DAI, colon length, H&E, ELISA, 16S rRNA, metabolomics; n=8 for ELISA. One-way ANOVA, $P<0.05$. Wu: total SCFAs 391.80 versus 181.67 μmol/g in model; endpoint-specific P UV. | Different models/regimens; assay n varies. Purity and treatment-exposure details need primary verification; microbial/metabolic associations do not establish mediation. |
| Quercetin (Wei et al., 2025; Gao et al., 2024; Jiang et al., 2024; Wang et al., 2024) | Flavonoids are widely distributed in plants and medicinal herbs. Mouse models of DSS-induced colitis (C57BL/6 or C57BL/6J, male). Cell models: Caco-2, RAW264.7, BMDMs, primary neutrophils. | Wei: barrier markers↑, NETs and NF-κB readouts↓, AhR activation. Gao: M2/M1 balance improved; cGAS–STING readouts↓. Jiang: colitis↓; CXCL8–CXCR1/2 and IL-17A/NF-κB readouts↓. Wang: AhR-associated protection attenuated by CH223191. | Wei 2025: C57BL/6N male; 2% DSS 7 days; QUE 20/50 mg/kg oral 9 days; MES 227.5 mg/kg; n=8. Gao 2024: C57BL/6J male; 3% DSS 7 days; QUE 30 mg/kg i.p.; RU.521 10 mg/kg i.p.; n=8. Jiang 2024: C57BL/6 male; 3% DSS 7 days + water 4 days; QUE 5/10 mg/kg oral gavage 11 days; mesalazine 600 mg/kg; n=10. Wang 2024: C57BL/6 male; 3% DSS 6 days; QUE 25/50/100 mg/kg oral gavage 10 days; FICZ 1 μg/mouse i.p.; CH223191 10 mg/kg i.p.; n=8. | Distinct study-specific doses/routes; no pooled efficacy estimate. Target dependence is supported mainly by pharmacological interventions. |
| Kaempferol (Qu et al., 2021; Tang et al., 2025) | Kaempferol, a widely occurring flavonoid. Qu: female C57BL/6J mice, DSS. Tang: DSS mice/RAW264.7; kaempferol hydrogel co-loaded with rhubarb nanovesicles. | Qu: colitis/permeability↓; barrier proteins↑; LPS–TLR4–NF-κB readouts↓; FMT-transferred protection. Tang: kaempferol/rhubarb-vesicle hydrogel improved colitis and macrophage polarization; free kaempferol had limited effects. | Qu: Female C57BL/6J mice; 4 groups (n=10): NC, Kae, DSS, DSS+Kae; Kae 50 mg/kg/day oral for 14 days; 3.5% DSS day 8–14; DAI, colon length, H&E, FITC-dextran, qRT-PCR, IHC, ELISA, 16S rRNA, GC-MS, FMT. Tang: C57BL/6 mice; 3% DSS for 5 days; groups: Model, CMHCD, 5-ASA, Kae, Kae/CMCHD, Kae/CMCHD@RNs (15/30 mg/kg Kae equivalent); n=3; DAI, colon length, spleen index, MPO, H&E, ELISA, IHC (Occludin, Claudin-3, ZO-1), macrophage polarization, biodistribution. | Qu: preventive, single-dose design. Tang: n=3; rhubarb vesicles and hydrogel preclude assigning all benefit to kaempferol. |
| Astragalin (Peng et al., 2020) | Natural flavonoid found in *Moringa oleifera* Lam., Radix astragali, *Morus alba* L., *Senna alata* (L.) Roxb., etc. Male C57BL/6J mice; DSS-induced acute colitis. No cell experiments reported. | Colitis, inflammatory cytokines, LPS and NF-κB phosphorylation↓; MUC2/occludin/ZO-1 mRNA↑. Microbiota changes included Escherichia–Shigella↓ and Lachnospiraceae/Ruminococcaceae↑; associations with LPS were correlational. | 7 groups (n=8): control; DSS+PBS; DSS+DMSO; DSS+AG 50/75/100 mg/kg; DSS+5-ASA 50 mg/kg. 3% DSS in drinking water for 7 days, then normal water for 2 days. AG dissolved in 20 μL DMSO + 180 μL PBS, oral gavage daily (200 μL). | Purity >98%; microbiota assessed in three groups. No microbial transfer/depletion test. |

| Material and reference | Natural source and reported system | Reported observations | Design and quantitative information | Verification and limitation |
|---|---|---|---|---|
| Vitexin (Zhang et al., 2022) | Flavonoid (apigenin 8-C-glucoside) mainly from *Crataegus* spp. (hawthorn) and other edible plants. Male BALB/c mice; DSS-induced acute colitis. | Colitis, IL-1β/IL-6/TNF-α and p-p65/p-IκB/p-STAT1↓; barrier markers↑, permeability↓. IL-10 alone unchanged; microbiota composition altered. | 5 groups (n=8): control; 3% DSS; DSS + 5-ASA 100 mg/kg; DSS + vitexin 20 or 80 mg/kg. 3% DSS in drinking water for 7 days; oral gavage daily for 7 days; sacrifice on day 8. | Purity >98%; microbial/pathway causality untested. Differences from 5-ASA barrier readouts do not establish a distinct mechanism. |
| Hyperoside (Xia et al., 2025; Cheng et al., 2021) | Flavonol glycoside (quercetin-3-O-D-galactoside), from *Hypericum* spp., *Crataegus* spp., and other plants. Xia: male C57BL/6 mice; DSS-induced colitis; NCM460 and HT-29 cells. Cheng: male C57BL/6 mice; DSS-induced colitis; Caco-2 cells and murine colonic lamina propria cells. | Xia: barrier integrity↑; autophagy-associated Snail degradation; chloroquine reversed protection. Cheng: colitis↓, Th17↓/Treg↑; MKRN1↓ and PPARγ stabilization; T0070907 attenuated protection. | Xia: Male C57BL/6 mice; 2.5% DSS 7 days + water 3 days; groups: Normal, DSS, HYP 20/40 mg/kg, mesalazine 150 mg/kg; n=6; oral gavage day 1–10. In vitro: NCM460/HT-29; LPS 1 μg/mL; HYP 5/10/20 μM; TER, FD4, WB/IF, mRFP-GFP-LC3; CQ 40 mg/kg i.p.; CQ/MG132/3-MA in vitro. Cheng: Male C57BL/6 mice; 3.5% DSS for 7 days; groups: control, DSS, HYP 3/10/30 mg/kg, 5-ASA 200 mg/kg; n=5; HYP after DSS for 7 days; T0070907 5 mg/kg + HYP 30 mg/kg, n=6; MLN flow cytometry, Co-IP/ubiquitination, Caco-2 cells. | Xia/Cheng: small groups and pharmacological rather than genetic pathway tests; different DSS strengths, doses, and treatment windows. |
| Nobiletin (Chen et al., 2025) | Polymethoxylated flavonoid from citrus fruit peels. Female C57BL/6J mice (8 weeks); DSS-induced acute colitis. IEC-6 cells (LPS-stimulated) for in vitro studies. | Colitis and ferroptosis-associated injury↓; barrier proteins/SLC7A11/GPX4↑. CETSA/DARTS supported HIF-1α engagement; HIF-1α knockdown attenuated protection. Microbiota–ferroptosis link remains associative. | 3 groups (n=7): Control, DSS (3% DSS for 7 days), DSS + NOB (40 mg/kg/day, oral, 10 days). No positive control. DAI, colon length, spleen index, H&E, WB/IHC (ZO-1, Occludin, Claudin-1, HIF-1α, ACSL4, PTGS2, SLC7A11, GPX4), MDA, GSH, 16S rRNA, serum metabolomics. In vitro: IEC-6 cells, LPS 1 μg/mL, NOB 12.5/25/50 μM; CCK-8, C11-BODIPY, FerroOrange, GSH, WB; si-HIF-1α. Mean ± SD, t-test/one-way ANOVA, P<0.05. | Single dose, no positive drug. Microbial functional predictions and metabolomics do not establish microbial mediation of ferroptosis. |
| Naringenin (Li et al., 2025) | Flavonoid reported in citrus fruits, tomatoes and cherries; male BALB/c mice; DSS colitis. | Colitis↓; Nrf2/HO-1/NQO1↑ and NF-κB/TNF-α/IL-1β↓. SPR supported Nrf2 binding (KD 20.0 μM); ML385 partially reversed protection. | 3.5% DSS for 7 days; Exp 1 (n=8): Control, Model, SASP 200 mg/kg, NAR 25/50/100 mg/kg, oral gavage for 7 days. Exp 2 (n=8): Control, Model, SASP 200 mg/kg, NAR 100 mg/kg, ML385 30 mg/kg, NAR 100 mg/kg + ML385 30 mg/kg. | SPR/ML385 support involvement, but no genetic Nrf2 test or exposure–response analysis; network predictions remain exploratory. |
| Naringin (Cao et al., 2021) | Flavonoid from Rutaceae grapefruit fruit/peel (bitter component of citrus juices). Male C57BL/6 mice (18–20 g, 4–5 weeks); DSS-induced UC. | Colitis, cytokines, MDA, iNOS and COX-2↓; GSH/SOD/occludin/ZO-1↑. Microbiota composition altered; naringin-only group showed no colitis symptoms. | 6 groups (n=10): Control; Model (DSS 8 g/kg, 0.6 g/mL by oral gavage); Nar 25/50/100 mg/kg + DSS; Nar 100 mg/kg alone. Day 1–7: control received saline; model received DSS; Nar+DSS received DSS in the morning + naringin in the afternoon; Nar alone received naringin only. DAI, colon length/weight, H&E, IF (TNF-α, IL-6, IL-1β), serum ELISA (TNF-α, IL-6), MDA/GSH/SOD, WB (iNOS, COX-2, Occludin, ZO-1), 16S rRNA (control, model, Nar 100 mg/kg + DSS; n=5). One-way ANOVA with Tukey's test, P<0.05. | DSS delivered by gavage; no positive drug. Microbiota n=5; causal mediation untested. |

| **Material and reference** | **Natural source and reported system** | **Reported observations** | **Design and quantitative information** | **Verification and limitation** |
|---|---|---|---|---|
| Neohesperidin (Ju et al., 2024) | Flavonoid reported in citrus. Male C57BL/6J mice; DSS colitis; FMT into antibiotic-treated recipients. | Colitis, cytokines, MAPK/NF-κB phosphorylation and permeability↓; claudin-3/ZO-1↑. FMT from treated donors conferred protection. | 5 groups (n=6): Control; DSS (2.5% w/v, days 15–21); NEO 50 mg/kg alone; DSS + NEO 25 mg/kg; DSS + NEO 50 mg/kg. NEO oral gavage from day 1 to 21; sacrifice on day 22. FMT: donors (n=18) Control/PBS/NEO 50 mg/kg; recipients (n=18) antibiotic-treated for 4 weeks, then 200 µL fecal suspension daily for 14 days, followed by 2.5% DSS. One-way ANOVA with Tukey's test, $P<0.05$. | No positive drug; FMT supports microbial contribution, but necessary strains/metabolites remain unidentified. |
| Isoliquiritin (Miao et al., 2024) | Flavonoid glycoside mainly isolated from licorice (*Glycyrrhiza* spp.). Male Sprague-Dawley (SD) rats (150 ± 10 g); TNBS-induced UC. Caco-2 cells with LPS-induced inflammation for in vitro validation. | Colitis, inflammatory cytokines, and MDA↓; IL-10/SOD↑. TLR2/TLR4 and HMGB1–MyD88–NF-κB/MAPK-associated readouts↓; TLR5/TLR9 unchanged. Similar anti-inflammatory responses in Caco-2 cells. | 5 groups (n=10): NC; MC (TNBS 80 mg/kg intrarectal); PC (5-ASA 50 mg/kg); isoliquiritin-L (25 mg/kg); isoliquiritin-H (50 mg/kg). After TNBS modeling, isoliquiritin was given for 7 days. Body weight, DAI, colon length, H&E, IHC/IF, ELISA (IL-1β, IL-4, IL-6, IL-10, PGE2, TNF-α, MDA, SOD), qRT-PCR/WB (TLR2/4/5/9, MyD88, IRAK1, TRAF6, NF-κB, p38, JNK, HMGB1, caspase-1, caspase-3). Pharmacokinetics: 12 SD rats, 25/50 mg/kg gavage, HPLC-UV, Tmax ~2 h. In vitro: Caco-2 + LPS 1 µg/mL + isoliquiritin; ELISA/qRT-PCR/WB/IF. Bioinformatics: DESeq2/EBSeq, FDR<0.01, FC≥2, GO. ANOVA with Tukey's HSD, $P<0.05$. | Some assays n=3; cellular concentration UV. PK indicated low plasma exposure; causal HMGB1/TLR pathway test absent. |
| Troxerutin (Wang et al., 2021) | Flavonoid; trihydroxyethylated derivative of rutin. Female C57BL/6 mice (8 weeks, 20–22 g); DSS-induced UC. Caco-2 cells for in vitro validation. | Colitis, inflammation, oxidative injury, apoptosis, and fibrosis-associated markers↓; epithelial junction proteins↑. Nrf2/HO-1↑; AKT/MAPK phosphorylation↓. Caco-2 oxidative injury and junction disorganization reduced. | 5 groups (n=8): normal control; DSS model; DSS + troxerutin 100 mg/kg; DSS + troxerutin 200 mg/kg; DSS + 5-ASA 200 mg/kg. 2.5% DSS in drinking water for 8 days, then normal water for 2 days; oral treatment for 10 days. In vitro: Caco-2 cells, troxerutin for 24 h, 2.5% DSS for 6 h; CCK-8, ROS, JC-1, IF. Bioinformatics: 283 clinical drugs screened; 109 troxerutin targets, 83 UC-related. One-way ANOVA with Dunnett's test; $P<0.05$. | Animal experiments unblinded; data analysts blinded. Pathway changes lack causal perturbation. |
| Lonicerin (Lv et al., 2021) | Flavonoid glycoside isolated from *Lonicera japonica* Thunb. (Japanese honeysuckle) flower buds. Female C57BL/6 mice; DSS-induced UC. THP-1 cells and bone marrow-derived macrophages (BMDMs) for in vitro validation. | Colitis and macrophage NLRP3 activation↓; ATG5-dependent autophagic degradation of NLRP3↑. Target-engagement assays supported EZH2 involvement; EZH2 overexpression or ATG5 knockdown reversed protection. | Female C57BL/6 mice (6–8 weeks, 20–22 g, n=6/group). Exp 1: normal; DSS (2.5% DSS for 7 days + water for 3 days); lonicerin 3/10/30 mg/kg; 5-ASA 200 mg/kg; oral gavage daily for 10 days. Exp 2: normal; DSS; scramble; EZH2 plasmid (10 µg i.c.); lonicerin 30 mg/kg + scramble; lonicerin 30 mg/kg + EZH2 plasmid. In vitro: THP-1/BMDMs, LPS + ATP/nigericin/MSU, lonicerin 3/10/30 µmol/L. Mean ± SEM, one-way ANOVA, $P<0.05$. | EZH2 overexpression/ATG5 knockdown strengthens mechanistic evidence; cell-specific in vivo necessity and exposure remain unresolved. |

| Material and reference | Natural source and reported system | Reported observations | Design and quantitative information | Verification and limitation |
|---|---|---|---|---|
| Isorhamnetin (Ru et al., 2024) | O-methylated flavonol; intermediate metabolite of quercetin in plants and animals; found in leaves, flowers, and fruits. Male C57BL/6 mice (5–8 weeks); DSS-induced UC. MC38 and HT1080 cells for in vitro ferroptosis validation. | Colitis, cytokines, and ferroptosis-associated injury↓. Cell assays showed lipid peroxidation/$Fe^{2+}$↓ and NRF2/HO-1/GPX4↑; iron chelation demonstrated in vitro. Ferroptosis-inhibition IC50 was approximately 2.6 μM (MC38) and 4.5 μM (HT1080). | 3 groups (n=5): Control; DSS + PBS; DSS + ISO. 2.5% DSS drinking water 7 days + normal water 3 days. ISO 15 mg/kg i.p. daily in 5% DMSO/PBS; DSS groups received an equal volume of vehicle. Sacrifice on day 10. In vitro: MC38/HT1080; RSL3 1 μM, erastin 10 μM, FIN56 20 μM; ISO 0.01–10 μM; CCK-8, PI, C11-BODIPY, FerroOrange, WB, IF (NRF2), docking, UV-Vis iron chelation. One-way/two-way ANOVA, P<0.05. | Single i.p. dose, n=5; no positive drug. Tumor-cell ferroptosis assays and in vitro chelation do not establish in vivo selectivity. |
| Luteolin (Yang et al., 2025) | Flavonoid present in various vegetables and fruits, and in medicinal plants such as *Lonicera japonica* Thunb. flower buds and chrysanthemum. Male BALB/c mice (8 weeks, 18–22 g); DSS-induced UC. | Colitis/cytokines↓; mucus and tight-junction markers↑. Microbiota and plasma metabolites altered; α-diversity unchanged. Conflicting metabolite directions are not pooled. | Male BALB/c mice (8 weeks, 18–22 g, n=24; 8/group); 3 groups: Control; DSS; DSS + Lut. Lut 25 mg/kg in 5% CMC-Na, oral gavage daily for 14 days; control and DSS received equal volume of CMC-Na. From day 8–14, DSS and Lut groups received 3% DSS in drinking water; control received water. Sacrifice on day 14. | No positive drug or microbial causal test. Abstract and Results conflict on metabolite directions. |
| Dihydromyricetin (Ma et al., 2024) | Reported in *Nekemias grossedentata* (Hand.-Mazz.) J.Wen & Z.L.Nie; male C57BL/6 mice, DSS; D-HL-60/BMDN neutrophils and Caco-2/HIEC-6 barrier models. | Colitis, permeability, and NET-associated markers↓; ZO-1/occludin↑. Neutrophil-conditioned medium injured epithelial barriers; dihydromyricetin or DNase I attenuated injury. DMOG counteracted protection, supporting HIF-1α-associated involvement. | 4 groups: control; DSS; DSS + DHM 50 mg/kg; DSS + DHM 100 mg/kg. 3.5% DSS in drinking water for 7 days; DHM by gavage once daily for 7 days; control and DSS received vehicle. In vitro: D-HL-60 and BMDNs; PMA 200 nM + ionomycin 4 μM or LPS 25 μg/mL; DHM 6.25/12.5/25 μM; DNase I 5 U/mL; cf-DNA, qPCR, WB, IF. Caco-2/HIEC-6 monolayer co-culture. Network pharmacology; DMOG rescue. One-way ANOVA with Tukey's test; P<0.05. | Allocation n=8 versus figure n=6; no positive drug. DMOG supports involvement but is not a cell-specific genetic test. |
| Apigenin (Huang et al., 2025; Ji et al., 2025) | Widely occurring flavonoid. Huang: male DSS mice, mast-cell-deficient/MrgprB2 conditional-knockout models and mast/epithelial cells. Ji: male BALB/c DSS mice and LS174T cells. | Huang: colitis and mast-cell activation↓; MRGPRX2/B2 engagement supported by SPR and genetic models. Ji: goblet-cell apoptosis/ER stress↓; SERCA2↑; cellular knockdown abolished protection. Benefit persisted after antibiotic depletion. | Huang et al., 2025: Male C57BL/6, KitW-sh/W-sh, and B2-CKO mice; 5% DSS 5 days; groups: NOR, UC, SASP 100 mg/kg, API 10/20/40 mg/kg (n=6). API by gavage. | Huang: mouse/human receptor differences limit extrapolation. Ji: antibiotic-treated mice are not germ-free; no goblet-cell-specific in vivo SERCA2 test. |
| Galangin (Yang et al., 2023) | Reported in *Alpinia officinarum* Hance; female C57BL/6 DSS mice; HEK293 and THP-1 cells. Human biopsies provided target-expression data, not treatment evidence. | Colitis/permeability and NLRP3 activation↓. HSP90β engagement supported by CETSA/DARTS; FASN-related lipogenesis↓. HSP90β or FASN overexpression reversed relevant effects. | Gavage galangin 40 mg/kg/day for 10 days; 2.5% DSS for 7 days, then water for 3 days; n=6/group. Controls: normal/DSS, 5-ASA 100 mg/kg, 17-AAG 50 mg/kg i.p.; HSP90β overexpression arms. Cells: 2.5/5/10 μM; HSP90β/FASN overexpression. Mean±SEM; ANOVA/t-test; P<0.05 threshold. | Single dose; overexpression is not cell-specific. Metabolomics used THP-1 cells rather than colon tissue. |

| **Material and reference** | **Natural source and reported system** | **Reported observations** | **Design and quantitative information** | **Verification and limitation** |
|---|---|---|---|---|
| Genistein (Jia et al., 2024) | Isoflavone (4′,5,7-trihydroxyisoflavone) mainly from soybean (*Glycine max* (L.) Merr.). Male C57BL/6J mice (20–24 g); DSS-induced UC. | Colitis/cytokines↓; mucus and junction markers↑; macrophage markers shifted toward M2. Fecal acetate/propionate/butyrate↑; microbiota composition altered. | 4 groups (n=8): NOR; DSS; DSS + genistein 20 mg/kg (LGE); DSS + genistein 40 mg/kg (HGE). 3% DSS in drinking water for 10 days. Genistein in 0.5% CMC-Na by intragastric administration daily for 10 days; NOR/DSS received CMC-Na. Sacrifice on day 11. | No positive drug; main n=8, some assays n=5. Microbial and macrophage mechanisms remain associated. |
| Acacetin (Ren et al., 2020) | Flavone; reported in acacia honey and citrus fruits; also reported in *Saussurea involucrata* (Kar. & Kir.) Sch.Bip., *Turnera diffusa* Willd. ex Schult. and *Robinia pseudoacacia* L. Female C57BL/6 mice; 3.5% DSS acute colitis; RAW264.7 and BMDM cells. | ↓ weight loss, diarrhoea, colon shortening and histological injury; ↓ F4/80+ macrophage infiltration; ↓ NO/iNOS/COX-2; ↓ IL-1β/TNF-α/IL-6; ↑ microbial diversity; ↑ Firmicutes; ↓ Proteobacteria/Deferribacteres; ↓ Escherichia-Shigella and Faecalibaculum. | Oral acacetin 50 or 150 mg/kg/day, days 1–9; DSS days 3–9; 50 mg/kg selected as optimal. Initial n=10/group; disease outcomes n=7; 16S rRNA n=5; qPCR/WB n=3. In vitro: LPS-stimulated RAW264.7; IFN-γ/LPS BMDM. No positive drug control. | No positive drug control; some assays n=3–5. Microbial mediation and direct targets untested. |
| Tricin (Li et al., 2021) | Plant-derived flavone (5,7,4′-trihydroxy-3′,5′-dimethoxyflavone); isolated from wheat straw; also reported in grains and bamboo. Male BALB/c mice; 4.5% DSS acute colitis; RAW264.7 cells. | Colitis, MPO, inflammatory cytokines, and MDSCs↓; Treg and microbiota profiles altered. In cells, inflammatory mediators and nuclear p-p65↓ without inhibition of p65 translocation. | In vitro: LPS 2 μg/mL; tricin 12.5/25/50 μM; SSZ 2 mM; RAW264.7; n≥3. In vivo: oral tricin 75/100/150 mg/kg/day for 7 days with 4.5% DSS; SSZ 200 mg/kg/day; n=6–12/group; 16S rDNA n=12 total (control, DSS, tricin 75, tricin 150). | High DSS severity limited reference-drug response; no causal NF-κB or microbiota test. |
| Eriodictyol (Hu et al., 2021) | Plant-derived flavanone widely distributed in foodborne plants; exact experimental source and purity not specified. Male Wistar rats; TNBS-induced colitis; no cell model. | ↓ body weight loss, histological score, crypt destruction, mucosal ulceration and inflammatory infiltration; ↓ MPO+ cells; ↓ serum TNF-α, IL-1β, IL-6, IL-2 and IL-12; ↑ IL-10; ↑ SOD, CAT and GSH-Px; ↓ MDA; ↓ colonic TLR4, p-IκBα and p-p65/p65; ↑ IκBα. | Oral eriodictyol 5/20/50 mg/kg/day, days 1–7; TNBS 100 mg/kg in 0.25 mL 50% ethanol, intrarectal; control and TNBS groups; n=6/group; no positive drug control; ELISA, IHC and Western blot; Mean ± SD; P<0.05 or <0.01 vs TNBS; exact effect sizes not reported. | No positive drug; experimental purity/source UV. TLR4/NF-κB necessity untested. |
| Tiliroside (Zhuang et al., 2021) | Purified tiliroside >98%; experimental botanical source unspecified. Female C57BL/6 DSS mice; male BALB/c TNBS mice; BMDM/THP-1 cells. | DSS/TNBS colitis and macrophage M1 markers↓; limited direct effects on T-cell/barrier genes. Glycolysis↓ through enhanced proteasomal HIF-1α degradation. Macrophage depletion abolished protection. | DSS: 2.5% DSS 7 days + 3 days water; tiliroside 12.5/25/50 mg/kg/day oral gavage, days 1–10; 5-ASA 200 mg/kg; n=8/group. TNBS: 2 mg TNBS in 40% ethanol, intrarectal; tiliroside 25/50 mg/kg. In vitro: BMDMs/THP-1; M1: LPS 100 ng/mL + IFNγ 20 ng/mL; M2: IL-4 20 ng/mL; tiliroside 10/20/40 μM, 24 h; n=3 independent. Glycolysis: 2-NBDG, lactate, glycolytic gene qPCR, Seahorse ECAR/glycoPER. HIF-1α: CHX chase, MG132, BafA1. Macrophage depletion: clodronate liposomes. | Macrophage depletion supports involvement; direct binding and in vivo HIF-1α necessity remain untested. |

| Material and reference | Natural source and reported system | Reported observations | Design and quantitative information | Verification and limitation |
|---|---|---|---|---|
| Wogonin (Liu et al., 2025) | Plant-derived flavone; reported in *Scutellaria baicalensis* Georgi root; purified wogonin >98%. Male C57BL/6J WT and Bcrp−/−Mrp2−/− mice; 2.5% DSS UC; HT29 and Caco-2 cells with BCRP/MRP2 knockdown. | Colitis and barrier injury↓ in WT mice; protection attenuated in Bcrp/Mrp2 double-knockout mice despite higher plasma exposure. Colonic/subcellular drug distribution altered; cellular transporter knockdown changed wogonin/wogonoside accumulation. | Oral wogonin 50/100 mg/kg/day, starting 7 days before 2.5% DSS and continued to day 19; vehicle 2.5% DMSO–40% PEG-400; n=8/group; no positive drug control. PK: UHPLC-MS/MS, 12 time points (5–720 min). Subcellular fractions: nuclear, mitochondrial, cytosolic, ER, lysosomal. Cells: lentiviral BCRP/MRP2 knockdown; MTT; UHPLC-MS/MS. Proteomics: 4D label-free mitochondrial; RT-qPCR/WB/ELISA validation. | Global transporter knockout confounds systemic and intestinal effects; AFM necessity untested. |
| Formononetin (Xiao et al., 2024) | Reported in *Astragalus mongholicus* Bunge, *Pueraria montana* var. *lobata* (Willd.) Maesen & S.M.Almeida ex Sanjappa & Predeep and *Trifolium pratense* L.; commercial compound ≥98%, source plant unspecified. Male C57BL/6 DSS mice; ABX/FMT. | Colitis↓; macrophage M1/M2 balance improved; microbiota/metabolites altered. Antibiotic depletion abolished benefit, and FMT transferred protection. Docking/MD predicted interactions, without direct target validation. | DSS: 2.5% DSS for 7 days; FN 25/50/100 mg/kg/day oral gavage for 10 days; 5-ASA 300 mg/kg/day; n=10–12/group; microbiota n=5. Antibiotic arm: vancomycin 100, neomycin 200, metronidazole 200, ampicillin 200 mg/kg for 5 days, then 2% DSS for 7 days; FN 100 mg/kg/day from day 7 for 10 days. FMT: 0.2 mL fecal suspension/day for 5 days, then 2% DSS for 7 days. Flow cytometry, ELISA, IF, 16S rRNA, untargeted LC-MS, docking/MD. | FMT supports microbial contribution; residual compound/metabolites and specific mediators remain unresolved. Docking does not demonstrate binding. |

## Table S5 Terpenoids

| Material and reference | Natural source and reported system | Reported observations | Design and quantitative information | Verification and limitation |
|---|---|---|---|---|
| Oleanolic acid 28-O-β-D-glucopyranoside (Wang et al., 2025) | Semisynthetic triterpenoid glycoside, purity 98%; reported natural occurrence in *Panax ginseng* C.A.Mey. Male SD TNBS rats; RSL3-injured Caco-2 cells. | Colitis, inflammation, iron/MDA and Th17/Treg ratio↓; junction proteins, GSH/SOD and Nrf2/HO-1/GPX4/SLC7A11↑. Similar protection in RSL3-injured Caco-2 cells; CETSA supported target engagement. | TNBS 100 mg/kg in 50% ethanol intrarectal; OAG 5/10/20 mg/kg/day oral gavage from day 2 for 15 days; 5-ASA 100 mg/kg; n=10/group. Multi-omics: control, model, OAG-H (n not clearly reported). qPCR/WB validation. Caco-2: RSL3 1 μM for 24 h; OAG 4 μM pretreatment for 2 h; Fer-1 4 μM. Docking and CETSA. | Semisynthetic test material; no in vivo pathway-necessity test. CETSA and docking do not establish which target mediates efficacy. |
| β-Caryophyllene (Yeom et al., 2022) | Plant-derived sesquiterpene; extracted from cloves, the flower buds of *Syzygium aromaticum* (L.) Merr. & L.M.Perry; experimental BCP supplied by SFC Bio, purity not specified. Male C57BL/6J mice; 2.5% DSS-induced colitis; 16S rRNA microbiota analysis. | ↑ stool weight; restored colon contractility; ↑ colon length at 300 mg/kg; ↑ mucin; ↓ MPO; ↓ colonic TNF-α, IL-1β and IL-6; ↓ Proteobacteria, Enterobacteriaceae and Peptostreptococcaceae; ↑ Bacilli/Lactobacillaceae. Body-weight loss and submucosal/muscle-layer gap were not restored. | Oral BCP 30/150/300 mg/kg for 7 days before 2.5% DSS for an additional 7 days; control received water; n=12/group; no positive drug control. | No positive drug control; purity by UV. Weight loss was not restored; microbial causality untested. |
| (-)-Fenchone (Araruna et al., 2024) | Plant-derived bicyclic monoterpene; reported in *Foeniculum vulgare* Mill., *Thuja occidentalis* L. and *Lavandula stoechas* L.; experimental substance from Sigma, purity not specified. Male Wistar rats; acute and chronic-relapsing TNBS-induced colitis. | Acute and chronic: ↓ ulcerative lesion area, lesion score, colon weight/length ratio and diarrhea index; ↓ MDA and MPO; ↑ GSH and SOD; ↓ NF-κB immunostaining; ↓ IL-1β and TNF-α; maintained IL-10 and TGF-β; ↑ ZO-1 immunostaining. Effects at 150 mg/kg comparable to prednisolone 2 mg/kg. | Acute: TNBS 10 mg in 0.25 mL 50% ethanol intrarectal; (-)-fenchone 37.5/75/150/300 mg/kg oral, 48/24/1 h before TNBS and 24 h after; evaluated at 48 h; prednisolone 2 mg/kg; n=7–10/group. Chronic-relapsing: TNBS on day 1 and day 14; (-)-fenchone 150 mg/kg/day oral for 21 days; evaluated on day 21; prednisolone 2 mg/kg; n=7–10/group. | Chronic model used one dose; purity by UV. Semi-quantitative IHC lacks causal target validation. |
| Ursolic acid (Sheng et al., 2021) | Plant-derived pentacyclic triterpenoid carboxylic acid; reported in plants and foods such as lavender and apple peel; experimental UA from MedChemExpress, purity 99.27%, exact botanical source not specified. Male C57BL/6 mice; 3% DSS acute colitis; transcriptomics, 16S rRNA, and immune-infiltration analysis. | Prevention improved weight loss and colon shortening; treatment improved colon shortening only. Histological injury/IL-6↓; transcriptomic inflammatory and immune-infiltration signatures altered. Microbial richness decreased; Shannon diversity unchanged. | DSS 3% in drinking water for 5 days. UA 200 mg/kg oral gavage. Prevention: from 24 h before DSS, once daily for 7 days. Treatment: from 3 days after DSS, once daily for 4 days. Vehicle control and DSS groups; n=8/group; no positive drug control. | Preventive/therapeutic windows differ; transcriptome n=3. Immune infiltration inferred computationally; microbial causality untested. |
| Betulinic acid (Zhuang et al., 2025) | Plant-derived pentacyclic triterpenoid acid; botanical source not specified; experimental betulinic acid from MedChemExpress (HY-10529). Male C57BL/6 mice; 4% DSS acute colitis; anxiety-like behavior tests. | Free drug and BA-CS-SA microspheres improved colitis; formulation reported greater benefit. Claudin-1↑; TNF-α/IL-1β↓; anxiety-like behavior improved. Microspheres showed pH-sensitive in vitro release. | DSS 4% w/v in drinking water for 7 days. Free betulinic acid 20 mg/kg/day oral gavage for 14 days; BA-CS-SA at equivalent dose for 14 days. Groups: control, DSS, free betulinic acid, BA-CS-SA. No positive drug control; n not clearly reported. | n UV; no blank-carrier control or colonic drug quantification. Behavioral improvement may follow reduced colitis. |

| Material and reference | Natural source and reported system | Reported observations | Design and quantitative information | Verification and limitation |
|---|---|---|---|---|
| 18β-Glycyrrhetinic Acid (Peng et al., 2025) | Plant-derived pentacyclic triterpenoid aglycone; major constituent of *Glycyrrhiza uralensis* Fisch. ex DC. root/rhizome extract; experimental 18β-GA purity ≥98% (PubChem CID 10114). Male SD rats; TNBS-induced colitis (in vivo ULE); Caco-2 cells with TNBS + LPS (in vitro ULE and 18β-GA). | Extract in rats: colitis/injury↓, junction proteins↑. Isolated 18β-GA in Caco-2: WNT3A/β-catenin/ZO-1/occludin↑ and TNF-α↓; IWR-1 blocked and SKL2001 mimicked effects. | Rats: TNBS 100 mg/kg in 75% ethanol, intrarectal; ULE 50/100/200 mg/kg/day oral gavage from day 4 to day 12; SASP 0.36 g/kg/day; n=6/group. Caco-2: TNBS 250 µmol/L + LPS 10 µg/mL; ULE 1/2.5/5 µg/mL; 18β-GA 0.3/0.75/1.5 µmol/L; SASP 50 µmol/L; IWR-1 5 µmol/L; SKL2001 2.5 µmol/L; 48 h; n=3–6. | Isolated 18β-GA was tested only in cells; rat efficacy belongs to the extract. Wnt modulation was pharmacological, not a direct-binding or genetic test. |
| Pachymic Acid (Zhang et al., 2025) | Fungal triterpenoid; reported in *Wolfiporia cocos* (F.A.Wolf) Ryvarden & Gilb. sclerotium; experimental PA purity ≥98% (Chengdu Herbpurify), exact source not specified. Male C57BL/6 mice; 3% DSS acute colitis. | Colitis, cytokines, ROS/MDA and DNA-damage/apoptosis readouts↓; barrier proteins, GSH/SOD↑; NF-κB phosphorylation↓. | 3% DSS in drinking water for 7 days; PA 5 mg/kg/day oral gavage for 7 days; control 0.5% CMC; n=6/group; no positive drug control. | Single dose; WB/IF n=3; no positive drug control. NF-κB necessity untested. |
| Patchouli alcohol (Wu et al., 2020) | Plant-derived tricyclic sesquiterpene; isolated from *Pogostemon cablin* (Blanco) Benth.; experimental PA purity 98% (Chengdu Must Bio-Technology). Male SD rats (TNBS colitis); female BALB/c mice (DSS colitis). | TNBS/DSS colitis, cytokines and NF-κB phosphorylation↓; tight-junction proteins and MUC1/MUC2↑. | TNBS rats: TNBS 70 mg/kg in 50% ethanol intrarectal; PA 7.5/15/30 mg/kg/day oral gavage for 7 days starting 24 h after induction; SASP 150 mg/kg; n=8/group. DSS mice: 3.5% DSS 7 days + water 14 days + 3.5% DSS 7 days; PA 15/30 mg/kg/day oral gavage from day 14 to day 27; n=6/group; no positive drug control. Vehicle: 2% Tween 80. | Sex/species differ across models; DSS arm lacks positive drug. NF-κB necessity untested. |
| Geniposide (Zhuge et al., 2023) | Iridoid glycoside; major bioactive compound from the dried fruit of *Gardenia jasminoides* J.Ellis; experimental geniposide ≥98% (Aladdin). Male C57BL/6 mice; 3% DSS acute colitis; Caco-2 cells with LPS. | Colitis, oxidative injury, and NF-κB activation↓; barrier markers/Nrf2-associated antioxidant responses↑. KEAP1 engagement supported by co-IP/DARTS/CETSA; ML385 reversed cellular protection. | Mice: 3% DSS for 7 days; geniposide 20 or 60 mg/kg/day oral gavage for 14 days; control and DSS groups; n=8/group; no positive drug control. Caco-2: geniposide 5 or 10 µM for 24 h, then LPS 1 µg/mL for 2 h; ML385 used as an Nrf2 inhibitor; n=3 independent. | ML385 supports Nrf2 involvement; in vivo genetic necessity and pharmacological exposure remain unresolved. |
| Sweroside (Li et al., 2025) | Secoiridoid glycoside reported in *Swertia mussotii* Franch., *Swertia pseudochinensis* H.Hara, *Lonicera japonica* Thunb., *Cornus officinalis* Siebold & Zucc. and *Plumeria rubra* L.; commercial compound ≥98%. Male C57BL/6J DSS mice; HUVECs. | Colitis/permeability↓; barrier integrity and microcirculation improved. VEGF/Hippo-associated signals and endothelial angiogenic behavior altered; VEGF165 and GA-017 partially reversed cellular effects. | Mice: 3% DSS; SOS 25 or 50 mg/kg/day i.p.; mesalazine 400 mg/kg oral gavage; control, DSS, DSS+Mesa, DSS+SOS-L, DSS+SOS-H and SOS-H alone; n=10/group. HUVECs: 0.5% DSS; SOS 100 µM; VEGF165 20 ng/mL; GA-017 10 µM. | i.p. compound versus oral comparator; duration UV. HUVECs are not intestinal endothelium; docking remains predictive. |

| Material and reference | Natural source and reported system | Reported observations | Design and quantitative information | Verification and limitation |
|---|---|---|---|---|
| Perillyl alcohol (Puppala et al., 2022) | Plant-derived monoterpene; reported in lavender, peppermint, and cherries; experimental POH from Sigma-Aldrich, 96% pure, exact botanical source not specified. Male C57BL/6 mice; chronic restraint stress + 2.5% DSS colitis; HEK293 NF-κB reporter cells. | Stress-associated colitis, cytokines, and TLR4/NF-κB/JAK2/STAT3 readouts↓; oxidative and behavioral outcomes improved. TNF-α-induced NF-κB reporter activity↓ in HEK293 cells. | Mice: RS 6 h/day, day 1–28; 2.5% DSS day 8–14; POH 100/200 mg/kg/day oral from day 14/15 to day 28; vehicle corn oil; groups: vehicle, RS, DSS, RS+DSS, RS+DSS+POH 100, RS+DSS+POH 200; n=8/group; no positive drug control. In vitro: HEK293 NF-κB reporter; TNF-α 30 ng/mL; POH 6.25–100 μM; ROS and IF at 25/50 μM; n=3. | Combined stress/DSS model, no positive drug. Reporter cells are nonintestinal; direct brain effects cannot be separated from secondary improvement. |
| Ganoderic acid (Ye et al., 2025) | Fungal triterpenoid from *Ganoderma lucidum* (Curtis) P.Karst. fruiting bodies; experimental GA isolated/purified, purity >98%, exact molecular identity/composition not specified. Male C57BL/6J mice; 2.5% DSS acute colitis; co-housing and FMT; Caco-2/NCM460 monolayers. | Colitis/permeability↓; junction proteins↑. Co-housing/FMT transferred protection. Indole-3-acetaldehyde/glutamine/glutathione↑ and deoxycholic acid↓; separate metabolite treatments altered epithelial barrier function. | Mice: 2.5% DSS for 7 days; GA 16.5/50/150 mg/kg oral; SASP 150 mg/kg; preventive and therapeutic protocols; n=12/group main; co-housing and FMT used 50 mg/kg. 16S rDNA and untargeted metabolomics n=5–8. Caco-2/NCM460: 100 μM IAAld/Gln/GSH/DCA for 12 h, then TNF-α + IFN-γ 10 ng/mL for 24 h. | Ganoderic-acid identity/composition incompletely specified. FMT/co-housing cannot exclude non-microbial transfer; metabolite tests were separate cellular interventions. Fungal name retained as reported; specimen identity not reauthenticated. |
| Limonene (Adriana et al., 2021) | Limonene reported in *Agastache mexicana* subsp. *mexicana* and *Agastache mexicana* subsp. *xolocotziana* Bye, Linares & T.Ram. essential oils; commercial compound, purity UV. Male BALB/c mice; oxazolone colitis. | Oxazolone colitis: DAI/injury/hyperalgesia↓; lesion area 18±4% versus 100% in model. IL-13↓, IL-5↑; TNF-α unchanged. | Oral limonene 10–300 mg/kg/day for 7 days, starting 24 h after oxazolone; SASP 100 mg/kg; n≥6/group. Presensitization: 3% oxazolone; induction: 1% intrarectal. Lesion area reported as 18±4% versus 100%; error convention/endpoint-specific P UV. | Oxazolone colitis only for the UC-related evidence; essential-oil effects cannot be assigned to limonene. No causal pathway test; TNF-α unchanged and IL-5 increased. |
| 1,8-cineole (Ma et al., 2023) | Plant-derived monoterpenoid oxide; main active compound of *Wurfbainia compacta* (Sol. ex Maton) Škorničk. & A.D.Poulsen fruit volatile oil (ACO); experimental 1,8-cineole from Merck (PHR1828), purity not specified. Male C57BL/6J mice; 2.5% DSS colitis; BMDMs, RAW264.7, Caco-2 and THP-1 cells. | Colitis/permeability and macrophage M1-associated responses↓; junction proteins/mucin↑. HSP90 engagement and ATPase inhibition supported; HSP90–NLRP3–SGT1 complex disrupted. Knockdown/overexpression supported cellular involvement; M2 effects were compartment-dependent. | Mice: 2.5% DSS for 7 days; ACO or 1,8-cineole oral gavage daily in 1% Tween 80; exact in vivo doses not stated in main text (see Supplementary Fig. S2); groups: CON, DSS, ACO, Cineole; no positive drug control; n not stated in main text. Cells: 1,8-cineole 50/100/200 μM for 12 h, then LPS 1 μg/mL + IFN-γ 50 μg/mL; BMDMs, RAW264.7, Caco-2/THP-1. | In vivo dose/n require supplementary-source verification. Volatile oil and isolated cineole are distinct interventions; genetic validation was cellular. |

| Material and reference | Natural source and reported system | Reported observations | Design and quantitative information | Verification and limitation |
|---|---|---|---|---|
| α-Terpineol (Khan et al., 2023) | Plant-derived monoterpene alcohol; reported in several plants (e.g., *Origanum vulgare* L., *Ocimum americanum* L.); experimental αTL source and purity not specified. Male Wistar rats; 4% DSS-induced colitis; no cell model. | ↓ DAI, colonic tissue damage and goblet-cell disintegration; ↓ mast-cell infiltration, MPO and NO; ↓ caspase-9 and caspase-3 activities; ↓ NF-κB-p65, iNOS, COX-2, p53, caspase-9 and cleaved caspase-3 immunostaining; ↑ connexin-43, survivin and Bcl-2 immunostaining; improved colon histoarchitecture. | Rats: control (corn oil 5 mL/kg, oral, 14 days); DSS (4% in drinking water, days 7–14); DSS + αTL (αTL 50 mg/kg/day, oral, days 1–14 + 4% DSS days 7–14); n=6/group; no positive drug control; sacrifice day 15. | Semi-quantitative tissue readouts; no causal perturbation. Abstract/Results marker directions conflict; Results used. |
| ar-Turmerone (Li et al., 2022) | Plant-derived sesquiterpene; main component of essential oil from rhizomes of *Curcuma longa* L., a plant used as food and medicine; experimental ar-Turmerone isolated from essential oil by column chromatography, purity not specified. Male KM mice; 2% DSS-induced colitis. | ↓ colon shortening, colonic tissue damage and histological score; ↓ colonic TNF-α and COX-2 immunostaining. Modulated gut microbiota: ↑ uncultured Bacteroidales bacteria Alloprevotella, Muribaculaceae, Lachnospiraceae and Ruminococcaceae; ↓ Helicobacteraceae, Desulfovibrionaceae and Marinifilaceae. PCA showed the ar-Turmerone group closer to control than to DSS. | Mice: 6–8 weeks, 36–40 g; groups: Con, DSS, DSS+ME (mesalazine 0.4 g/kg), DSS+TEO (2.64 mg/kg/day), DSS+ar-Turmerone-L/M/H (10/20/40 mg/kg/day). Days 1–7: oral gavage of vehicle or test agents; days 8–14: 2% DSS in drinking water + continued gavage; mesalazine started on day 8. Sacrifice on day 14. | n/purity UV; pretreatment differs from mesalazine timing. Taxon directions vary between analyses; microbial causality untested. |
| Asperuloside (Chen et al., 2021) | Plant-derived iridoid glycoside; reported in *Scleromitrion diffusum* (Willd.) R.J.Wang; experimental ASP from Baoji Chenguang Biotechnology, HPLC ≥98% (CAS 14259-45-1), exact botanical source not specified. Male KM mice; 2% DSS-induced chronic colitis; LPS-stimulated RAW264.7 cells. | Colitis/inflammation and oxidative injury↓; Nrf2/HO-1/NQO1↑; NF-κB activation↓. Nrf2 siRNA attenuated cellular effects; docking remains predictive. | Mice: 2% DSS-induced chronic colitis; ASP 125 or 500 µg/kg/day oral once daily (route reported for vehicle groups; exact ASP route not explicitly stated); VD3 4 IU/day as reference; groups: control, DSS, DSS+VD3, DSS+ASP 125, DSS+ASP 500; n=10/group in Methods but figure legends state n=11. In vitro: RAW264.7 pretreated with ASP 5/10/20 µM for 24 h, then LPS 1 µg/mL for 6 h; n=3. | Reported µg/kg doses retained without conversion. Route/chronic-DSS schedule unclear; Methods n=10 versus figures n=11. In vivo necessity untested. |
| β-myrcene (Almarzooqi et al., 2022) | Plant-derived monoterpene; reported in lemongrass oil (*Cymbopogon citratus* (DC.) Stapf), rosemary (*Salvia rosmarinus* Spenn.), hop and bay oils; experimental β-myrcene gift from Coastside Bio Resources, purity not specified. Male C57BL/6J mice; 2% DSS acute colitis; TNF-α-challenged HT-29 cells. | In vivo: ↑ colon length; ↓ DAI, MPO, colon inflammation score and histological damage; ↓ IL-6, IL-1β and TNF-α (protein and mRNA); ↓ COX-2 and iNOS (protein and mRNA); ↓ p-ERK, p-JNK, p-p38 and p-NF-κB p65 (Ser536). In vitro: no cytotoxicity up to 100 µM; ↓ TNF-α-induced COX-2, CXCL-1 and IL-8 mRNA. | Mice: 2% DSS for 7 days; β-myrcene 50 or 100 mg/kg body weight; sulfasalazine 50 mg/kg as positive control; control, DSS, DSS+β-myrcene 50, DSS+β-myrcene 100, DSS+SAZ; n=8/group for DAI/colon/MPO/ELISA, n=6 for mRNA, n=4–5 for WB; route/frequency not clearly stated in main text. Cells: HT-29; TNF-α 1 ng/mL for 24 h, then β-myrcene 25/50 µM for 12 h; viability tested up to 100 µM; n=6. | Route/frequency and purity UV; n varies by assay. HT-29 cells are tumor-derived; pathway necessity untested. |

**Table S6 Polysaccharides and polysaccharide-based delivery materials**

| Material and reference | Natural source and reported system | Reported observations | Design and quantitative information | Verification and limitation |
|---|---|---|---|---|
| Purslane polysaccharides (Ning et al., 2024) | *Portulaca oleracea* L. aqueous polysaccharide-enriched fraction; carbohydrate 40.12±1.49%, Mw 26.5 kDa. Male C57BL/6J mice; 4% DSS. | Colitis, cytokines, and oxidative injury↓; mucus/junction markers↑. SCFAs/retinol and microbiota profiles altered; CD19+ B cells/IgA↑, CD138+ plasma cells↓. | Mice: 4% DSS days 8–15; POP 400 or 800 mg/kg/day oral for 8 days; SASP 200 mg/kg/day; control, DSS, POPL, POPH, SASP; n=8/group. | The fraction contains only ~40% carbohydrate; activity cannot be assigned to polysaccharide alone. No microbial causal test or B-cell depletion. |
| *Astragalus* polysaccharide (Zhang et al., 2025) | *Astragalus mongholicus* Bunge root arabinogalactan-rich fraction; Mw 104.9 kDa. Male BALB/c mice; 3% DSS; ABX/FMT. | Colitis, inflammation, and permeability-associated readouts↓; SCFAs/FFAR2/FFAR3↑; Th17↓/Treg↑. Antibiotic depletion abolished protection; FMT transferred benefit. TLR4/NF-κB/HDAC3/JAK2/STAT3-associated readouts↓. | Mice: 3% DSS days 8–14; APS 200 (L) or 400 (H) mg/kg/day oral gavage days 1–14; 5-ASA 100 mg/kg days 8–14; groups: control, DSS, 5-ASA, L-APS, H-APS; n=12/group. ABX: ampicillin 2 mg + neomycin 2 mg + metronidazole 2 mg + vancomycin 1 mg per mouse, oral gavage twice daily for 10 days; DSS days 11–20; APS 400 mg/kg days 1–20; n=10/group. FMT: donor APS group; recipient FMT group; n=10/group. | ABX/FMT support microbial contribution; specific SCFA–immune mediation untested. Fluorescent retention does not quantify intact-polysaccharide PK. |
| *Codonopsis pilosula* (Franch.) Nannf. polysaccharide (Zhou et al., 2025) | *Codonopsis pilosula* root crude polysaccharide, water extraction/ethanol precipitation; Mw 29.386 kDa, PDI 4.852. Male C57BL/6 mice; 3% DSS; FMT. | Colitis/cytokines↓; acetate/butyrate and GPR43/GPR109A↑; NLRP3/caspase-1/IL-1β/IL-18↓. FMT from treated donors conferred protection. | Mice: 3% DSS days 1–5, water days 6–7, sacrifice day 8; CPPS 300/600/1200 mg/kg/day (route not explicitly stated in Methods; described as gavage in Results) for 7 days; SASP 200 mg/kg as positive control; groups: control, DSS, CPPSL, CPPSM, CPPSH, SASP; n=8/group. FMT: donors DSS or CPPSH; recipients antibiotic-treated (metronidazole 1 g/L, vancomycin 0.5 g/L, ampicillin 1 g/L, neomycin sulfate 1 g/L) for 7 days, then 3% DSS for 5 days + 0.2 mL fecal suspension daily for 5 days; n=6/group. | Crude heterogeneous fraction; route/timing incompletely explicit. FMT supports microbial contribution; SCFA-receptor/NLRP3 necessity untested. |
| Coix seed polysaccharide (Ji et al., 2025) | *Coix lacryma-jobi* var. *ma-yuen* (Rom.Caill.) Stapf seed crude polysaccharide; Mw 663.414 kDa, glucose 99.79%. Male BALB/c mice; 3% DSS; ABX/FMT. | Colitis, LPS and cytokines↓; junction proteins/mucus↑. Microbiota and metabolites altered; antibiotic depletion abolished benefit, and FMT transferred protection. | CSP 200 mg/kg/day oral gavage; groups: NC, DSS, DSS+CSP (n=8/group). CSP given before DSS (starting day 1); 3% DSS from day 14. Antibiotic clearance: streptomycin 1 g/L + ampicillin 0.5 g/L + gentamicin 1 g/L + vancomycin 0.5 g/L in drinking water for 14 days; groups ABX, ABX+DSS, ABX+DSS+CSP. FMT: antibiotics 10 days, pure water 3 days, then 100 mg feces + 1 mL sterile saline, filtered 70 μm, centrifuged 600 g for 5 min; 10 μL/g/day supernatant by gavage every 2 days for 7 administrations; then 3% DSS 7 days. | Preventive, crude-fraction design. FMT supports contribution but cannot exclude residual material/metabolites; specific metabolite mediation untested. |

| **Material and reference** | **Natural source and reported system** | **Reported observations** | **Design and quantitative information** | **Verification and limitation** |
|---|---|---|---|---|
| *Dioscorea* polysaccharide (Zhang et al., 2025) | *Dioscorea* sp. (Ma yam cultivar); purified CYP-A, Mw 1540 kDa, total sugar 84.27%, protein 4.21%. Male C57BL/6J mice; 3% DSS. | Colitis and oxidative injury↓; mucus, ZO-1/claudin-1/Lgr5/sIgA↑. Microbiota and amino-acid/steroid-associated metabolites altered; associations were correlational. | Groups (n=10): Control, DSS, 5-ASA (100 mg/kg), L-CYP-A (100 mg/kg), M-CYP-A (200 mg/kg), H-CYP-A (400 mg/kg). First and third weeks: free water; second week: 3% DSS (w/v); CYP-A by gavage for 21 days; 5-ASA for 14 days in weeks 2–3; sacrifice on day 22. | Source species unresolved; purified fraction is cultivar-specific. No causal microbiota/metabolite intervention. |
| Mulberry polysaccharides (Feng et al., 2021) | *Morus alba* L. branch polysaccharide (96.5%) in PLGA nanoparticles (~202 nm). Female ICR mice, systemic LPS jejunal injury; RAW264.7 cells. | PLGA-RMP reduced jejunal injury/cytokines after systemic LPS; macrophage M2/Treg-associated responses↑. Formulation reported greater effects than free RMP; sustained release demonstrated in vitro. | In vitro: RAW264.7; LPS; PLGA-RMP non-toxic at 125 µg/mL; uptake assessed with PLGA-DEX-FITC; phenotype/cytokine assays (exact PLGA-RMP concentration not clearly stated). In vivo: female ICR mice (7 weeks, 20–22 g); groups n=6: PBS, PLGA-RMP 30 mg/kg, RMP 30 mg/kg, PLGA 30 mg/kg; oral gavage for 7 days; day 8 LPS 20 mg/kg i.p.; sacrifice 6 h later. | Systemic-LPS jejunal injury is not a conventional colitis model. Formulation and free polysaccharide must be distinguished; cellular dose UV. |
| *Platycodon grandiflorus* (Jacq.) A.DC. polysaccharide (Liu et al., 2023) | *Platycodon grandiflorus* root crude polysaccharide; carbohydrate 78%. Male BALB/c mice, 3% DSS; lymphatic-ligation/perfusion and splenocyte assays. | Colitis/inflammation↓; Th1/Th17↓, Th2/Treg↑. Mesenteric lymphatic ligation attenuated colonic immune effects; splenocyte assays supported altered proliferation/differentiation. | 3% DSS for 7 days; PGP 100/200/400 mg/kg by gavage for 7 days starting the second day after DSS cessation; SASP 200 mg/kg as positive control; control and DSS received saline; n=6/group; sacrifice day 15. | Crude fraction; lymphatic ligation can have indirect effects. No direct lymphatic tracking or subset-specific immune-necessity test. |
| LBP 2-1 (Liu et al., 2025) | *Lycium barbarum* L. fruit acidic polysaccharide; Mw 102.2 kDa, PDI 1.247, carbohydrate 75.2%. Female C57BL/6J mice; 3.5% DSS. | Colitis and histological injury↓; microbial diversity↑ and composition altered. Cytokine, barrier-protein, and SCFA effects not established. | 4 groups n=10: NC, DSS, 5-ASA, LBP 2-1. 3.5% DSS days 1–7; sterile saline days 8–11; LBP 2-1 200 mg/kg/day oral gavage; 5-ASA positive control (dose not stated in main text); sacrifice day 12. | Single dose; 5-ASA dose UV. Microbiota changes lack causal tests; cytokine/barrier mechanisms were not measured. |
| *Wolfiporia cocos* (F.A.Wolf) Ryvarden & Gilb. polysaccharides (Wan et al., 2024) | *Wolfiporia cocos* sclerotium crude polysaccharide; hot-water extraction/ethanol precipitation. Male C57BL/6J mice; 3% DSS; ABX/FMT. | Colitis, cytokines, and NF-κB phosphorylation↓; junction proteins and fecal SCFAs↑. DAO/TGF-β/IFN-γ unchanged. Antibiotic depletion abolished benefit; FMT transferred protection. | groups: CON, DSS, DSS+PCP (n=10/group); PCP 800 mg/kg BW/day oral gavage; 3% DSS days 8–14; sacrifice after day 14. ABX: streptomycin 1.0 g/L + penicillin 1.0 g/L + metronidazole 1.0 g/L + vancomycin 0.5 g/L for 7 days, then 3% DSS for 7 days; groups ABX vs ABX-PCP (n=10/group). FMT: donors FMT vs PCP-FMT; recipients antibiotic-treated, transplanted once daily with 0.2 mL fecal slurry days 8–14, then 3% DSS for 1 week; n=10/group. | Single crude-fraction dose; FMT supports microbial contribution. Specific SCFA/receptor or NF-κB mediation untested. |
| Steamed *Polygonatum sibiricum* Redouté polysaccharide fraction PSSP-EF (Li et al., 2025) | Steamed *Polygonatum sibiricum* rhizome galactan-rich fraction; Mw 22.4 kDa, galactose 80.55 mol%. Male Kunming mice; 4% DSS. | Colitis, cytokines, LPS, and indoxyl sulfate↓; junction proteins↑. Microbiota composition altered and cecal SCFAs↑. | 4% DSS in drinking water for 9 days; groups (n=10): control, model, positive (mesalazine 100 mg/kg), PSSP-EF high (400 mg/kg) and low (200 mg/kg); oral gavage once daily for 9 days. | Processed fraction differs from raw rhizome polysaccharides. Microbiota/SCFA correlations lack causal intervention. |

| Material and reference | Natural source and reported system | Reported observations | Design and quantitative information | Verification and limitation |
|---|---|---|---|---|
| *Houttuynia cordata* Thunb. polysaccharides (Cen et al., 2022) | Plant-derived crude polysaccharide; whole plant of *Houttuynia cordata* (Saururaceae); mainly Glc, Gal, Ara and Rha (3.40:2.14:1.17:1.00) with trace Man and Xyl; exact source/purity not specified. Male C57BL/6 mice; 2.5% DSS-induced acute colitis; IEC-6 and Ana-1 cells. | Colitis, epithelial apoptosis, and cytokines↓; mucus/junction markers↑. Th17/Treg-associated readouts altered; protection lost in Foxp3- or RORγt-deficient mice. Cellular macrophage inflammation↓; microbial mediation untested. | 2.5% DSS for 10 days; HCPs 40 or 80 mg/kg/day oral for 10 days; SSZ 125 mg/kg/day as positive control; groups: control, DSS, HCPs 40, HCPs 80, SSZ; n=6/group. Transgenic mice: RORγt−/− and Foxp3−/−; control, DSS, HCPs 80; n=6/group. Cells: IEC-6 with $H_2O_2$ (1 or 1.5 mM) and HCPs 1/10/100 μg/mL; Ana-1 with LPS 1 μg/mL and HCPs 1/10/100 μg/mL. | Global Foxp3/RORγt deficiency may have developmental confounding. Microbial causality and direct target engagement untested. |
| *Phyllanthus emblica* L. polysaccharide (Li et al., 2024) | *Phyllanthus emblica* fruit purified polysaccharide; Mw 3670 kDa, carbohydrate 92.5%, uronic acid 68.7%. Male C57BL/6J mice; repeated DSS. | Chronic colitis and TNF-α/IL-6↓; colonic IL-10↑; AST/ALT unchanged. Microbiota and fecal metabolites altered, including deoxycholic acid↑; associations are study-specific. | 1.5% DSS drinking water for four cycles (4 days DSS + 7 days normal water, last cycle without recovery); groups (n=10): Control (AIN-93G diet + water), Model (AIN-93G diet + DSS water), PEPL (100 mg/kg PEP-1 + DSS), PEPH (200 mg/kg PEP-1 + DSS); PEP-1 administered daily (route not explicitly stated in main text; likely oral gavage); no positive drug control. | High-Mw fraction; route: UV. Chronic-model microbial/metabolite associations lack causal intervention. |
| *Crataegus pinnatifida* Bunge polysaccharide (Wei et al., 2025) | *Crataegus pinnatifida* fruit polysaccharide (CPP); Mw 81.247 kDa, GalA 55.14%. Male BALB/c mice; 3% DSS; antibiotic depletion/FMT and separate L-arginine arm. | Colitis, cytokines, and oxidative injury↓; mucus/junction markers↑. Antibiotic depletion abolished benefit; FMT transferred protection. Colonic L-arginine/ASS1↑; separate L-arginine treatment partly reproduced benefit. | Gavage CPP 100/200 mg/kg/day, days 0–21; 3% DSS days 14–21; 5-ASA 100 mg/kg days 14–21. n=8/group stated; group counts inconsistent. ABX/FMT and L-arginine arms n=6/group; L-arginine 2 g/kg/day. Mean±SD; t-test/ANOVA–Tukey; $P<0.05$ threshold; exact efficacy means UV. | Preventive design; allocation count inconsistent. FMT supports microbial contribution; specific taxa and ASS1 necessity were not directly tested. |
| Seabuckthorn polysaccharides (Ouyang et al., 2024) | Sea buckthorn berry (attributed to *Hippophae rhamnoides* L.; species authentication UV) polysaccharide (SBP); material preparation insufficiently described. Male C57BL/6J mice; 2.5% DSS; FMT. | Colitis, inflammation, and oxidative injury↓; barrier integrity and SCFAs↑. FMT from SBP-treated donors conferred protection, supporting a microbial contribution. | Oral SBP 200 mg/kg/day for 21 days before 2.5% DSS on days 22–28; day-29 endpoint. Normal/saline and DSS controls; no positive drug; n=10/group, histology n=6. FMT 200 μL/day for 3 weeks before DSS. Mean±SEM; one-/two-way ANOVA; $P\leq0.05$ threshold; exact efficacy means UV. | Single-dose pretreatment; material identity/composition incompletely reported. FMT does not identify necessary taxa/SCFAs or exclude transferred non-microbial factors. |
| Dandelion Root Polysaccharides (Yan et al., 2023) | *Taraxacum* sect. *Taraxacum* F.H.Wigg. root crude polysaccharide; carbohydrate 97.1%. Male C57BL/6 DSS mice; $H_2O_2$-injured IEC-6 cells. Reported as *Taraxacum officinale*; microspecies not resolved. | Colitis, iron/lipid-peroxidation injury and cytokines↓; GPX4/SLC7A11/Nrf2-associated readouts and barrier proteins↑. Similar responses in injured IEC-6 cells. Microbiota and fecal organic-acid profiles altered. | 3% DSS days 1–5; DP 150 or 300 mg/kg/day by gavage days 7–14; control received saline; groups: control, model, low-DP, high-DP; n=6/group. No positive drug control. In vitro: IEC-6 cells; $H_2O_2$ injury; DP 5, 10, 20 μg/mL; CCK-8, flow cytometry apoptosis, ELISA (4-HNE, MDA, GSH), RT-qPCR, WB, TEM, iron assay. | No positive drug; Nrf2/ferroptosis necessity untested. Organic-acid panel includes noncanonical SCFAs; source resolves only to a Taraxacum section. |
| Turmeric polysaccharide (Yang et al., 2021) | Turmeric rhizome polysaccharide; species and structural characterization unspecified. C57BL/6 mice, sex unspecified; repeated 1.5% DSS. | Repeated-DSS colitis↓; junction proteins/SCFAs/IAA/IAAld/AhR/IL-22↑. TNF-α, IL-17 and IL-10↓. Microbiota–tryptophan–AhR sequence proposed, without causal intervention. | Mice: C57BL/6; repeated 1.5% DSS cycles; TPS 100/200/400 mg/kg by gavage; groups: control, DSS, TPS 100, 200, 400; no positive drug control; n not clearly stated. | Species, n, and structural characterization incomplete. No causal microbial, AhR, or metabolite intervention. |

| Material and reference | Natural source and reported system | Reported observations | Design and quantitative information | Verification and limitation |
|---|---|---|---|---|
| *Dendrobium officinale* Kimura & Migo polysaccharide (Zeng et al., 2024) | *Dendrobium officinale* polysaccharide; Mw 14.3 kDa, mainly mannose/glucose. Female BALB/c mice; 4% DSS. | DAI and bleeding↓; goblet cells/junction transcripts↑. Microbiota/metabolites altered, including Enterobacteriaceae/Enterococcus↑. High-dose serum biochemical changes require safety interpretation. | 6 groups: CON, DSS, MES (mesalazine 100 mg/kg/day), DPL/DPM/DPH (DOP 50/100/200 mg/kg/day); normal water days 8–14; 4% DSS days 15–21; DOP and MES by gavage; sacrifice day 22; 120 mice total, but n per endpoint not clearly stated. | Endpoint n and dosing window unclear; high-dose biochemical changes require scrutiny. Microbial/metabolic mediation untested. |
| *Panax quinquefolius* L. polysaccharides (Ren et al., 2023) | *Panax quinquefolius* root water-soluble polysaccharide; carbohydrate 85.2%, uronic acid 31.9%, protein 2.1%. Male C57BL/6J mice; 2.5% DSS. | Colitis/cytokines↓; junction proteins and fecal SCFAs↑. High-dose microbiota shifts included Bacteroides/Shigella↑ and Lactobacillus↓; these directions should not be generalized as beneficial. | Groups n=8: C (control), DSS (2.5% DSS 7 days), Y (mesalazine 100 mg/kg/day), L/M/H WQP (50/100/200 mg/kg/day) for 7 days after DSS; no blank vehicle control beyond saline; survival differed among groups (DSS 2 deaths; Y/L/M 3; H 1). | Unequal mortality and unclear endpoint n may bias results. Microbial/SCFA associations lack causal intervention. |
| *Rehmannia glutinosa* (Gaertn.) Libosch. ex DC. polysaccharides (Lv et al., 2023) | *Rehmannia glutinosa* sulfated acidic polysaccharide; Mw ~65 kDa, reported purity 98%. Male ICR DSS mice; RAW264.7 cells. | Colitis/cytokines and NF-κB phosphorylation↓; occludin/ZO-1 and SCFAs↑; claudin-1 trend nonsignificant. Cellular IL-1β/IL-6↓, TNF-α unchanged. | Groups (n=8: control, DSS, DSS+RGP (400 mg/kg/day by gavage for 21 days before and during 7-day 3% DSS), RGP alone (400 mg/kg/day); no positive drug control. In vitro: RAW264.7; LPS 1 μg/mL; RGP 40/80/160 μg/mL. | Single-fraction dose; no positive drug. Microbiota/SCFA/NF-κB mediation untested. |

## Table S7 Saponins: related sapogenins and total saponin fractions

| Material and reference | Natural source and reported system | Reported observations | Design and quantitative information | Verification and limitation |
|---|---|---|---|---|
| Astragaloside IV (Zhang et al., 2024) | Plant-derived triterpenoid saponin; from roots of *Astragalus mongholicus* Bunge (a plant used as medicine); purity >98%. Human UC colon biopsies; male C57BL/6 mice; 2.5% DSS-induced acute colitis; Caco-2 cells with LPS. | Colitis/permeability↓; epithelial junction markers↑; PI3K/AKT phosphorylation↓. Pharmacological PI3K/AKT modulation supported involvement; microbiota changes were associative. | 2.5% DSS 7 days; AS-IV 100 mg/kg/day oral gavage; 5-ASA 150 mg/kg/day positive control; n=10/group. In vitro: Caco-2; LPS 100 μg/mL; AS-IV 150 μM. | Human biopsies are observational, not treatment evidence. Cellular pharmacological tests support involvement; microbial mediation untested. |
| Total saponins of Codonopsis (Liu et al., 2021) | *Codonopsis pilosula* (Franch.) Nannf. root total saponin fraction; yield 1.616%, constituent composition unspecified. Male Wistar rats; TNBS/ethanol colitis. | DAI, macroscopic/histological injury, MDA and IL-6/TNF-α/NF-κB readouts↓; SOD/IL-10↑; effects varied with dose. | 5 groups (n=10): control, TNBS model, SASP (0.3 g/kg/day), TSC low (0.4 g/kg/day, calculated as crude drug, ~10× adult clinical dose), TSC high (1.2 g/kg/day, ~30× adult clinical dose). TNBS 100 mg/kg in 50% ethanol, 0.25 mL, intrarectal ~8 cm; drugs by gavage once daily for 5 days before and 21 days after modeling. | Constituent composition unspecified; doses expressed as crude-drug equivalents. TNBS model and NF-κB expression alone do not establish UC-specific efficacy or causality. |
| Mogroside V (Tan et al., 2024) | Plant-derived triterpenoid saponin; from *Siraitia grosvenorii* (Swingle) C.Jeffrey ex A.M.Lu & Zhi Y.Zhang (monk fruit), a plant used as food and medicine; experimental MV purity >99%. Female C57BL/6J mice; 3% DSS-induced acute colitis; no cell model. | Colitis/permeability and ER-stress/apoptosis readouts↓; junction proteins↑. Tunicamycin reversed benefit; 4-PBA gave no significant additional benefit. | 5 groups (n=6): control, DSS, DSS+MV 25, 50, 100 mg/kg; 3% DSS for 7 days; MV by gavage during DSS; no positive drug control. Second experiment: control, DSS, DSS+MV 100 mg/kg, DSS+MV+4-PBA 500 mg/kg, DSS+MV+tunicamycin 1 mg/kg. | No positive drug; tunicamycin/4-PBA have broader effects. No genetic ER-stress necessity test. |
| Dioscin (Li et al., 2022) | Plant-derived steroidal saponin; reported in *Dioscorea* species and other plants used as medicines; experimental dioscin from Topscience (Shanghai), purity not specified. Male C57BL/6 mice; 2.5% DSS-induced acute colitis; no cell model. | ↓ body-weight loss, DAI, MDI, colon shortening and histological score; ↓ MDA; ↑ GSH and SOD; ↑ p-AMPK/AMPK; ↓ p-mTOR/mTOR; ↑ Beclin-1, LC3-II/I and ATG5; ↓ p62. AMPK inhibitor compound C reversed these effects. | Groups (n=8: control, dioscin alone, DSS, DSS+dioscin. Dioscin 40 mg/kg oral gavage for 7 days; 2.5% DSS for 5 days, then water for 2 days. Mechanism experiment: compound C 20 mg/kg i.p. daily for 7 days. | Single dose, no positive drug. Compound C has off-target effects; AMPK specificity remains unresolved. |
| Platycodin D (Guo et al., 2021) | Plant-derived triterpenoid saponin; from roots of *Platycodon grandiflorus* (Jacq.) A.DC., a plant used as medicine; experimental PLD purity ≥98%. Male C57BL/6J mice; 3% DSS-induced acute colitis; LPS-stimulated RAW264.7 macrophages; macrophage-depletion experiment. | Colitis/barrier injury↓; macrophage M1↓/M2↑; PI3K/Akt phosphorylation↑ and NF-κB activation↓. Macrophage depletion attenuated protection; cellular AMPKα knockdown reversed polarization effects. | Groups (n=8: control, 3% DSS, 3% DSS+PLD. 3% DSS days 7–14; PLD 10 mg/kg/day oral gavage days 0–13; no positive drug control. Macrophage depletion: clodronate liposomes or PBS liposomes 0.2 mL i.p. on days 6, 8, and 10; groups n=4. In vitro: RAW264.7; LPS 1 μg/mL for 6 h; PLD 2.5 or 5 μM pretreatment for 12 h; AMPKα siRNA transfection. | No positive drug; AMPKα knockdown was cellular. Clodronate depletion is not selective for one macrophage subset. |

| Material and reference | Natural source and reported system | Reported observations | Design and quantitative information | Verification and limitation |
|---|---|---|---|---|
| Ginsenoside Rb1 (Zhou et al., 2024) | Plant-derived triterpenoid saponin; *Panax ginseng* C.A.Mey. root; GRb1 CAS 41753-43-9, HPLC ≥98%. Male C57BL/6 mice; acute DSS colitis. | Body-weight loss↓, DAI↓, colon shortening↓, histological score↓; TNF-$\alpha$↓, IL-6↓, IL-10↑; ZO-1↑, occludin↑, E-cadherin↑; VDR↑, PPAR$\gamma$↑, p-NF-$\kappa$B p65↓. RNA-seq/network pharmacology/docking suggest a VDR–PPAR$\gamma$–NF-$\kappa$B network. | GRb1 20/40/80 mg/kg gavage, 10 days; control/DSS saline; n=6/group; no positive drug. | No positive drug or causal VDR/PPAR$\gamma$/NF-$\kappa$B intervention; mechanism remains associative/predictive. |
| Ginsenoside Rg1 (Zhang et al., 2025) | Plant-derived triterpenoid saponin; *Panax ginseng* C.A.Mey. root/stem; G-Rg1 CAS 22427-39-0, purity 98%. Male obese B6/JGpt-Lepem1cd25/Gpt mice; DSS colitis. | DAI↓, colon length↑, colonic weight↓, histological score↓; IL-10↑, IL-6↓, IL-1$\beta$↓, IL-17A↓. mTfh subset balance modulated; abstract reports cmTfh↑ and emTfh↓, but some subset directions conflict with Results. Bcl-6↓, Blimp-1↑; FOXO1A↑, Roquin1/2↑, p-STAT3/STAT3↓. | G-Rg1 200 mg/kg gavage; 2.5% DSS; n=10/group; no positive drug. | Obese-mouse model; dosing schedule and mTfh directions conflict. No Bcl-6 necessity test. |
| Ruscogenin (Li et al., 2024) | Plant-derived steroidal sapogenin from *Ophiopogon japonicus* (Thunb.) Ker Gawl. root; purity 99%; $C_{27}H_{42}O_4$, MW 430.62. Male C57BL/6 mice (6–8 weeks, 18–25 g); DSS colitis; THP-1 and HIEC cells. | In vivo: body-weight loss↓, DAI↓, colon length↑, histological score↓; IL-6/TNF-$\alpha$/MCP-1↓; ZO-1/E-cadherin/occludin↑; NLRP3, caspase-1 p20, GSDMD, IL-1$\beta$, IL-18↓; TLR4/NF-$\kappa$B p65↓. In vitro: LPS/nigericin-induced caspase-1-dependent pyroptosis↓; caspase-4 non-canonical pyroptosis largely unaffected; TLR4 overexpression reversed protection. | Mice: 3.5% DSS 7 days + normal water 1 day; Rus 0.5/1/2 mg/kg gavage; groups Ctrl/DSS/Rus; day 8 endpoint; n UV; no positive drug. Cells: THP-1/HIEC; LPS 100 ng/mL + nigericin 1 µM; Rus 2.5/5/10 µM; TLR4 overexpression plasmid. | n UV; no positive drug. TLR4 overexpression was cellular; in vivo necessity remains unresolved. |
| Ophiopogonin D (Zhang et al., 2024) | Plant-derived steroidal saponin from *Ophiopogon japonicus* (Thunb.) Ker Gawl. root. Male C57BL/6 mice (6 weeks, 18±2 g); DSS colitis; 16S rRNA. | Colitis/inflammatory readouts↓; mucus/junction proteins↑; microbiota altered. Separate propionate treatment improved colitis but did not establish mediation of ophiopogonin D effects. | 3% DSS 7 days; OphD 10/40/160 mg/kg screen, 40 mg/kg effective, 20/40/80 mg/kg safety (13 days); n=6/group; no positive drug. | No positive drug; separate propionate arm does not establish mediation. CALP nomenclature is inconsistent in the source. |

## Table S8 Phenolic metabolites and enriched fractions

| Material and reference | Natural source and reported system | Reported observations | Design and quantitative information | Verification and limitation |
|---|---|---|---|---|
| Purple yam polyphenol (Yang et al., 2023) | *Dioscorea alata* L. tuber polyphenol-enriched 80% methanol extract; 16 constituents profiled. Female C57BL/6 mice; DSS and AOM/DSS models. | DSS colitis: body-weight loss↓, DAI↓, colon length↑, histopathological score↓; E-cadherin↑, occludin↑, claudin-1↑; $CD11b^{+}/F4/80^{+}$ macrophage infiltration↓; TNF-α↓, IL-6↓, IL-1β↓, IL-17A↓, CXCL1↓, MCP-1↓; p-NF-κB p65↓, p-STAT3↓. AOM/DSS CAC: tumor number/size↓, PCNA↓; p-NF-κB p65↓, p-STAT3↓; survival 90% vs 70%. | DSS: female C57BL/6, n=8/group; 3% DSS 7 days; PYPE 50/100 mg/kg gavage 10 days; no positive drug. CAC: female C57BL/6, n=10/group; AOM 10 mg/kg + 3 cycles of 3% DSS; PYPE 100 mg/kg daily; endpoint week 10. | Polyphenol mixture; active constituents undefined. No positive drug or causal pathway test; CAC is a separate outcome. |
| Gallic acid (Leng et al., 2024; Yu et al., 2023) | Commercial gallic acid. Leng: male C57BL/6J mice, DSS; ABX/FMT and splenocytes. Yu: male BALB/c DSS mice; RAW264.7 cells; purity 98.5% in vivo and ≥98% in vitro. | Leng: colitis/permeability↓, ILC3↑; microbiota/bile acids altered; ABX removal benefit and FMT-transferred protection. Yu: colitis/cytokines and NLRP3-associated readouts↓; LPS-induced macrophage inflammatory responses↓. | Leng: 100 mg/kg from day 0; 2.5% DSS for 8 days; 5-ASA 60 mg/kg; disease n=6, histology n=3, flow n=4; route/frequency UV. Yu: oral 40/80/120 mg/kg/day on days 7–12 after 7-day 3.5% DSS; 5-ASA 100 mg/kg; disease n=6, molecular n=3. At 120 mg/kg, colon length $P<0.05$ versus DSS; histology at all doses $P<0.01$. Cells: 50/100/200 µM, 24 h; mean±SD. | Leng: ABX/FMT support microbial contribution, not specific bile-acid/ILC3 necessity. Yu: NLRP3-associated changes lack pathway blockade; results must remain study-specific. |
| Ellagic acid (Xiong et al., 2025) | Plant-derived polyphenol (ellagic acid, dimeric derivative of gallic acid), widely distributed in fruits and nuts; experimental source/purity UV. Male BALB/c mice (8 weeks, 40 ± 2 g); DSS-induced UC; 16S rRNA. | Colitis, inflammation, and oxidative injury↓; Nrf2↑; NF-κB/NLRP3/pyroptosis-associated readouts↓. Microbiota composition altered; short-term safety endpoints showed no detected abnormalities at 35 mg/kg. | Experiment 1: CON vs EA 35 mg/kg oral gavage for 15 days, n=10. Experiment 2: CON, DSS, EA + DSS, EA (n=10/group); 3% DSS in three stages (days 1–3, 6–9, 11–14), EA 35 mg/kg oral gavage for 15 days; no positive control. Statistics: one-way ANOVA + Dunnett's, Mean ± SEM, P<0.05/0.01. | Single dose; no positive drug. NLRP3 and microbial causality untested; short-term findings do not establish long-term safety. |
| Chebulagic acid (Zhang et al., 2025) | Hydrolyzable tannin from *Terminalia chebula* Retz. fruits; HPLC ≥98%. Male C57BL/6 mice (8–10 weeks, 23 ± 2 g); DSS-induced acute colitis; 16S rDNA. | Body weight loss↓, DAI↓, colon length↑, histology score↓; MDA↓, SOD↑, GSH-PX↑, MPO↓; TNF-α↓, IL-1β↓, IL-6↓; ZO-1↑, occludin↑; TUNEL+ cells↓, Bax↓, cleaved caspase-3↓, Bcl-2↑; p-P38↓, p-ERK↓, p-JNK↓; nuclear NRF2↑, HO-1↑; p-NF-κB p65↓. Microbiota: Firmicutes↑, Proteobacteria↓; Escherichia_Shigella↓, Streptococcus↓, Clostridium_sensu_stricto_1↓; Faecalibaculum↑, Dubosella↑, Muribaculaceae↑. | 3% DSS for 7 days; CA 20/100 mg/kg gavage from 7 days before DSS; mesalazine 100 mg/kg positive control; n=8/group (some assays n=6–8; 16S n=3–4). | Pathway/microbiota changes lack causal intervention; microbial n=3–4. |

| Material and reference | Natural source and reported system | Reported observations | Design and quantitative information | Verification and limitation |
|---|---|---|---|---|
| 6-Gingerol (Li et al., 2024) | Plant-derived phenolic compound from *Zingiber officinale* Roscoe; purity >98%. Male C57BL/6J mice (6–8 weeks, 24–26 g); DSS-induced UC; plasma metabolomics; network pharmacology; Caco-2 cells. | Colitis/cytokines and ferroptosis-associated injury↓; GSH/SOD↑. Plasma lipid metabolites altered; ALOX5/ALOX15/PTGS2 interactions predicted by docking. | Mice: 3% DSS for 7 days; groups: control, DSS, SASP 0.45 g/kg, 6-G 50 mg/kg, 6-G 100 mg/kg; n=8; treatment 10 days. Statistics: Mean ± SEM; one-way ANOVA. | Docking/network predictions and ferroptosis markers do not establish target-mediated protection in vivo. |
| Ferulic acid (Ghasemi-Dehnoo et al., 2023) | Widely occurring phenolic acid; commercial ferulic acid ≥99.0%. Male Wistar rats; acetic acid colitis. | FA dose-dependently reduced macroscopic and histopathological damage; decreased MDA and NO; increased TAC, SOD, and CAT; downregulated mRNA expression of TLR4, TNF-α, IL-1β, IL-6, iNOS, NF-κB, caspase-3, caspase-8, and Bax; Bcl-2 mRNA unchanged. Proposed mechanism: inhibition of LPS-TLR4-NF-κB and NF-κB-iNOS-NO signaling pathways. | 48 rats, 6 groups (n=8): normal, model (0.8 mL 7% acetic acid intrarectally), FA 20/40/60 mg/kg by gavage 1 h after induction for 5 days, dexamethasone 2 mg/kg i.p. as standard drug; euthanized on day 6. Statistics: one-way ANOVA + Tukey's test; Mean ± SEM; P<0.05. | Acetic-acid injury model; mechanism inferred largely from mRNA. Reference drug did not improve all biochemical endpoints. |
| Caffeic acid (Xiu et al., 2025) | Plant-derived polyphenol (hydroxycinnamic acid); CAS 331-39-5, C9H8O4, MW 180.16, purity ≥98%; specific botanical source not stated. Adult *Drosophila melanogaster*; DSS-induced intestinal injury/UC model. | Drosophila intestinal injury, ROS and barrier leakage↓; survival/function↑. Mitochondrial and Toll/Imd-associated readouts altered. Docking-predicted interactions; this is not a mammalian UC model. | Adult flies, 4% DSS; caffeic acid 0.5/1 mM. Functional outcomes: n=6 vials; intestinal length/cell death n=12 flies; TUNEL n=5 flies; transcriptome n=3 vials. Duration UV. Log-rank/t-test/ANOVA–Tukey; mean±SEM. | Invertebrate intestinal injury only; no mammalian validation. Vials and individual flies are different experimental units. |
| Rosmarinic acid (Tang et al., 2025) | Plant-derived polyphenol; abundant in Lamiaceae; purity ≥98%. Male ICR mice (6–8 weeks, 25–35 g); DSS-induced colitis; NCM460 human colon epithelial cells stimulated with LPS. | RA attenuated colitis: body weight loss↓, DAI↓, colon shortening↓, histopathological score↓. Restored ZO-1, occludin, claudin-1. Inhibited p-AKT and increased Nrf2. PI3K inhibitor LY294002 abolished RA-induced upregulation of Nrf2 and TJPs. | Mice: 3% DSS for 7 days; RA 20/40 mg/kg/day by gavage from day 3; AZA 13 mg/kg/day positive control; n=10/group. Cells: LPS 20 µg/mL; RA 0.25–1 µM; AZA 1 µM; LY294002 10–20 µM. WB for TJPs, p-AKT, AKT, Nrf2. Statistics: one-way ANOVA + Tukey; Mean ± SD; P<0.05. | Cellular inhibitor results and p-AKT direction require context; no in vivo PI3K/Nrf2 necessity test. |
| Resveratrol (Tang et al., 2025) | Polyphenol from grapes, berries, *Reynoutria japonica* Houtt.; Sigma #R5010, purity UV. Male C57BL/6 mice (25 ± 2 g); DSS UC; isolated colonocytes; AAV-MDM2. | Colon length↑, inflammatory infiltration↓, mucosal injury↓, occludin/ZO-1↑. scRNA-seq: colonocyte apoptosis/P53 pathway↓. In vitro: DNA fragmentation↓, apoptosis↓, MMP↑, G1 arrest; MDM2↑, P53/PUMA/BAX↓, BCL2↑. Nutlin-3a reversed; AAV-MDM2 protected. | Mice: 5% DSS d1–7; resveratrol 50 mg/kg i.p. d8–24; n=6. AAV-MDM2/GFP 1 × 10^11 vg i.v.; 5% DSS 7 days; n=6. scRNA-seq n=2. In vitro: TGF-β 30 ng/mL 24 h; resveratrol 50/75/100 µM 24 h. | Single i.p. dose; scRNA-seq n=2. No in vivo MDM2 loss-of-function test. |

| Material and reference | Natural source and reported system | Reported observations | Design and quantitative information | Verification and limitation |
|---|---|---|---|---|
| Vanillic acid (Zhao et al., 2025) | Phenolic compound found in various plants; MCE, purity UV. Male C57BL/6J mice (6 weeks); DSS-induced acute UC; FMT. | Colitis/cytokines↓; junction proteins↑; cPLA2/COX-2/PGE2↓; macrophage M2-associated responses↑. Akkermansia muciniphila enriched; FMT transferred protection. | Six groups (n=6): control, DSS, SASP 200 mg/kg, VA 100/200/400 mg/kg; 2.5% DSS days 8–14; daily gavage. FMT: antibiotic depletion 7 days, FMT 3 weeks, then 2.5% DSS 1 week. Statistics: one-way ANOVA or t-test; Mean ± SEM. | FMT cannot exclude residual material/metabolites; no macrophage-necessity test. Network/docking evidence is predictive. |
| Curcumin (Zhang et al., 2025) | Polyphenol from *Curcuma longa* L.; Guangrun Biotechnology, UV purity. Male BALB/c mice (8 weeks); DSS-induced UC; RNA-seq; network pharmacology; docking. | Colonic injury, cytokines, and MPO↓; SphK1/p-p65 readouts↓. SphK1/NF-κB interactions were predicted by docking, but binding was not demonstrated. | Groups (n=6): Control, DSS, curcumin-L 100 mg/kg/day, curcumin-H 200 mg/kg/day; 30-day treatment; route: UV. Statistics: one-way ANOVA; Mean ± SEM. | Route/DSS regimen and purity UV; no positive drug or causal SphK1 test. |
| Protocatechuic acid (Yang et al., 2023) | Natural phenolic acid (3,4-dihydroxybenzoic acid) found in edible vegetables, fruits, and nuts; PCA >98% (MACLIN). Male C57BL/6 mice (6–8 weeks); DSS-induced UC; Caco-2 cells with Erastin-induced ferroptosis. | Body weight↑, colon length↑, DAI↓; IL-6↓, IL-12↓, TNF-α↓; occludin↑; Bacteroidetes↑, Verrucomicrobiota↑; iron↓, MDA↓, GSH↑, GPX4↑; mitochondrial damage↓. In Caco-2: viability↑, GSH↑, GPX4↑, occludin↑, MDA↓, ROS↓. | Mice: 48 mice, 6 groups (n=8): control, DSS, DSS + SASP 100 mg/kg, DSS + PCA 5/10/20 mg/kg; 3.5% DSS for 7 days; intragastric daily. Statistics: one-way ANOVA or rank-sum; Mean ± SD. | Doses >30 mg/kg reportedly worsened bleeding. Microbial and in vivo ferroptosis mediation untested. |
| Acteoside (Guo et al., 2022) | Phenylethanoid glycoside from *Ligustrum expansum* Rehder (a plant used as a beverage); CAS 61276-17-3; purity UV. Male C57BL/6 mice (8 weeks, 20–24 g); DSS-induced UC; Caco-2 cells with DSS. | Body weight loss↓, colon shortening↓, DAI↓, mucosal inflammation↓; TNF-α↓, IL-1β↓, IL-6↓; MDA↓, CAT↑, GSH↑, SOD↑; occludin↑, ZO-1↑, claudin-2↓; TUNEL+ cells↓, Bax↓, cleaved caspase-3↓, Bcl-2↑; HO-1↑, HMGB1↓. SnPP (HO-1 inhibitor) reversed ACT effects on GSH, apoptotic proteins, and HMGB1, indicating HO-1-dependent HMGB1 inhibition. | Mice: 4 groups (n=6): control, DSS, DSS + ACT 30 mg/kg, DSS + ACT 60 mg/kg; 4% DSS for 7 days; ACT i.p. daily for 7 days. Statistics: one-way ANOVA + Tukey; Mean ± SD. | i.p. dosing; no positive drug. SnPP supports involvement but does not establish HO-1 specificity. |
| Gastrodin (Zhang et al., 2025) | Phenolic glycoside from *Gastrodia elata* Blume (plant used as medicine and food); purity >98%. Male C57BL/6 mice; DSS UC; 16S rRNA; metabolomics; MALDI-MSI; FMT. | Colitis/barrier injury↓; microbial tryptophan metabolites and AhR↑; NLRP3-associated readouts↓. FMT transferred protection; antibiotics attenuated benefit. | 6 groups (n=6): Control, DSS (2.5% DSS 7 days), GAS 25/50/100 mg/kg, 5-ASA 300 mg/kg; gavage 7 days. FMT: donor DSS/GAS; recipients DSS-FMT, GAS-FMT, GAS-FMT-ABX; ABX 3 days before and throughout for ABX; 0.2 mL daily for 7 days. | FMT/ABX support microbial contribution; specific strain/metabolite and AhR necessity remain unresolved. |
| Isochlorogenic acid A (Tang et al., 2023) | Plant-derived dicaffeoylquinic acid; reported in *Lonicera japonica* Thunb. flower buds and chicory; CAS 2450-53-5, purity ≥98%. Male C57BL/6 mice (6–8 weeks); DSS-induced acute colitis; RAW264.7 and J774A.1 macrophages. | DAI↓, colon shortening↓, histological damage↓; peripheral neutrophils↓, colonic neutrophil and macrophage infiltration↓; TNF-α↓, IL-6↓, IL-1β↓, IFN-γ↓, CCL7↓, iNOS↓; Muc-2↑, ZO-1↑, ZO-2↑, occludin↑; p-STAT3/STAT3↓, p-p65/p65↓, p-IκBα↓, IκBα↑. No obvious hepatorenal toxicity. | Mice: 5 groups (n=6): control, DSS (2.5% DSS for 7 days), ICGA-A 25/50 mg/kg, SASP 100 mg/kg; treatment from day 3 for 5 days. In vitro: LPS 200 ng/mL + IFN-γ 20 ng/mL; ICGA-A 50/100/200 μM; BAY 11-7082 5 μM. | Cellular inhibitor evidence does not establish in vivo STAT3/NF-κB necessity; route/duration details require source check. |

## Table S9 Coumarins

| Material and reference | Natural source and reported system | Reported observations | Design and quantitative information | Verification and limitation |
|---|---|---|---|---|
| 7-Hydroxycoumarin (Liu et al., 2024) | Plant-derived coumarin (umbelliferone), present in medicinal plants and vegetables; source plant UV; purity >98%. Male ICR mice (6–8 weeks); DSS-induced UC; 16S rRNA; network pharmacology; docking/MDS. | Colitis, cytokines, and MPO↓; SOD↑. AKT1/EGFR and MAPK-associated readouts↓; microbiota composition altered. Network/docking predictions do not establish target necessity. | Mice: 5 groups (n=6): Control, DSS (3% DSS days 1–7), 7-HC 25/50/100 mg/kg orally days 1–10; no positive drug control. ELISA/qPCR n=4–6; WB n=3; 16S n=4. Network pharmacology, docking, MDS predictive. Statistics: ANOVA + LSD/Dunnett's T3; Mean ± SD. | No positive drug; microbial/target causality untested. Network/docking predictions are exploratory. |
| Imperatorin (Luo and Luo, 2021) | Plant-derived furocoumarin from *Angelica dahurica* (Hoffm.) Benth. & Hook.f. ex Franch. & Sav.; experimental source/purity UV. Male Sprague-Dawley rats (180–200 g); TNBS-induced colitis. | IMP reduced DAI and colon weight/length ratio, alleviated histopathological damage, and decreased ROS, TNF-α, and IL-6. Nrf2/ARE/HO-1 was biphasic: on day 3, IMP increased Nrf2, ARE, HO-1, and active ARE; on day 7, no significant difference; on day 14, the model increased Nrf2, ARE, and HO-1, and IMP dose-dependently suppressed them. | 72 male SD rats, 6 groups (n=4 per time point): control, TNBS model (70 mg/kg in 50% ethanol, intrarectal), IMP 15/30/60 mg/kg i.p. daily from day 2, SASP 50 mg/kg positive control; assessed at days 3, 7, 14. Statistics: t-test/one-way ANOVA; Mean ± SD; $P<0.05$. | Nrf2-associated response differs by time point; a uniform activation claim is inappropriate. No causal pathway test; purity UV. |
| Isoimperatorin (Fang et al., 2025) | Plant-derived furanocoumarin from *Angelica dahurica* (Hoffm.) Benth. & Hook.f. ex Franch. & Sav. (Baizhi), a plant used as medicine and food; MedChemExpress HY-N0286, purity UV. Male C57BL/6 mice; DSS-induced UC; NCM460 cells; Treg depletion. | Colitis/cytokines↓; Treg/Foxp3/IL-10 and epithelial repair markers↑. Treg depletion attenuated protection. Epithelial migration and Treg generation↑ in vitro; Th17 proportion largely unchanged. | Mice: 5 groups (n=6): normal, model (2.5% DSS 7 days + water 7 days), isoimperatorin 20/40 mg/kg, 5-ASA 100 mg/kg; gavage days 8–14. Treg depletion: anti-CD25 200 µg/mouse i.v. Day 7. In vitro: NCM460; CCK-8 0.1–100 µM; scratch/Transwell 0.3/1/3 µM; naive CD4+ T cells + CD3/CD28/TGF-β/IL-2; isoimperatorin 0.3/1/3 µM 72 h. Statistics: one-way ANOVA + Tukey; Mean ± SEM. | Anti-CD25 depletion is not fully Treg-specific; induction mechanism unresolved. Chemical spelling in the original title is inconsistent. |
| Isofraxidin (Huang et al., 2024) | Reported in *Eleutherococcus* sp. (originally Acanthopanax); commercial isofraxidin, purity UV. Male C57BL/6 DSS mice; NCM460 cells. | Reduced body weight loss, DAI, colon shortening, histological injury; ↓ TNF-α, IL-1β, IL-6; ↓ apoptosis (TUNEL, Bax, cleaved caspase-3; ↑ Bcl2); ↓ IL-17, HSP90, p-ERK, p-p38, p-JNK; ↑ ZO-1; ↓ S1PR1. S1PR1 overexpression reversed isofraxidin effects on inflammation, apoptosis, and IL-17 signaling. | Mice: 5 groups (n=6): control, DSS (2.5% DSS for 1 week + water for 3 days), isofraxidin 5/10/15 mg/kg orally once daily for 10 days; no positive drug control. Cells: NCM460; DSS 0.2 µg/mL; isofraxidin 5/10/15 µM; S1PR1 overexpression plasmid. Statistics: t-test or one-way/two-way ANOVA + Tukey; Mean ± SD; $n \geq 3$. | Botanical species/purity UV. S1PR1 overexpression was cellular; no in vivo necessity test. |

| Material and reference | Natural source and reported system | Reported observations | Design and quantitative information | Verification and limitation |
|---|---|---|---|---|
| Scopoletin (Alqudah et al., 2025) | Scopoletin; source plant/purity UV. Male mice; DSS colitis. | Reduced body weight loss, DAI, colon shortening, histological injury, and MPO activity; ↓ TNF-α, IL-1β, IL-12 (protein/mRNA in colon and MLN); ↑ PPARγ; ↓ p-P65 and p-IκB; ↓ NLRP3, ASC, caspase-1, IL-1β; ↓ E. coli, ↑ Lactobacillus and Bifidobacteria; ↑ ZO-1 and occludin. | Six groups (n=10): control, DSS (2.5% for 7 days), scopoletin 1/1.5/2 mg/kg orally from 14 days before and during DSS, dexamethasone 2 mg/kg positive control; 21 days. Statistics: one-way ANOVA + Dunnett's test; $P \leq 0.05$. | Microbiota assessed by culture, not sequencing. No causal microbial/pathway intervention; purity UV. |

## Table S10 Lignans

| Material and reference | Natural source and reported system | Reported observations | Design and quantitative information | Verification and limitation |
|---|---|---|---|---|
| Dehydrodiisoeugenol (Yi et al., 2025) | Reported in *Myristica fragrans* Houtt.; purified dehydrodiisoeugenol ≥99.98%. DSS mice; RAW264.7/BMDM inflammatory assays. Separate tumor models are not UC evidence. | DSS colitis: injury/cytokines↓; junction proteins↑. In macrophages, inflammatory mediators and NF-κB/MAPK/NOD2 readouts↓. Cancer-cell and xenograft effects are distinct from the colitis evidence. | DSS 3.5%; oral dehydrodiisoeugenol 5/10/20 mg/kg, days 1–8; SASP 50 mg/kg; normal/model controls; n=5/group. LPS/IFN-γ-stimulated macrophages: 2.5–10 μM. Cancer doses omitted from the UC comparison. | n=5/group; no in vivo NOD2 loss-of-function test. Cancer outcomes cannot be treated as colitis efficacy. |
| Sesamin (Cao et al., 2025) | Plant-derived lignan from sesame seeds/oil; purity UV. Male BALB/c mice; DSS-induced UC; LPS-stimulated macrophages. | Colitis/barrier injury↓; macrophage M2 markers/DUSP1↑ and p-ERK↓. DUSP1 knockdown affected cellular responses; CETSA supported ERK engagement. FMT transferred protection; microbial–DUSP1 mediation remains unresolved. | Mice: 3% DSS for 7 days; groups (n=6): control, DSS, SSM 25/100 mg/kg, 5-ASA 5 mg/kg; gavage for 7 days. FMT groups (n=6): control, DSS, SSM, SSM-FMT; antibiotics then FMT. Statistics: one-way ANOVA/t-test + Tukey; Mean ± SD; $P<0.05$. | DUSP1 knockdown was cellular; FMT cannot exclude residual compound/metabolites. Microbial–DUSP1 mediation unresolved. |
| (–)-Syringaresinol (Liu et al., 2024) | Lignan phytoestrogen from various medicinal plants; in-house purified, 98.2% purity. LPS-induced Caco-2 cells; DSS-induced UC male BALB/c mice. | Caco-2: ↑TEER; ↓TNF-α, IL-6, IFN-γ, COX-2; ↑ZO-1, occludin, claudin-1, E-cadherin. Mice: ↑body weight, colon length, tight junction proteins; ↓DAI, spleen coefficient, serum cytokines, bacterial translocation, intestinal damage; preserved colonic ultrastructure. Transcriptomics/WB: PI3K-Akt/MAPK/Wnt; ↓p-PI3K, p-Akt, p-NF-κB, p-p38 MAPK, p-GSK-3β, β-catenin; ↑E-cadherin. | Caco-2: LPS 50 μg/mL; SYR 25/50/100 μM; TEER, ELISA, IF. Mice: 6 groups (n=10): control, DSS (3.5% for 7 days), SASP 200 mg/kg, SYR 10/20/40 mg/kg orally for 10 days. | No causal in vivo pathway test; enantiomer-specific effects were not compared. |
| Schisandrin (Wang et al., 2023) | Lignan from *Schisandra chinensis* (Turcz.) Baill. fruit; purity >98%. Male C57BL/6 mice (5–6 weeks, 18–20 g); DSS-induced UC; THP-1 cells; SD rats for PK. | Colitis, NLRP3-associated inflammation, and SGK1 readouts↓; mucus↑; microbiota/bile acids altered. Cellular inflammasome responses↓; docking predicted SGK1 interaction. | Mice: 6 groups (n=8): control, model (3% DSS 7 days), 5-ASA 50 mg/kg/day, SCH 20/40/80 mg/kg/day; SCH pre-treated 3 days before DSS; daily gavage. Nigericin experiment: 4 groups (n=6): control, colitis, Nig+SCH 40, Nig. PK: SCE 0.95 g/kg, HPLC-MS/MS. In vitro: THP-1 + PMA/LPS/ATP, SCH 20/40/80 μM. | No causal microbial or in vivo SGK1 test. PK arm used Schisandra extract (SCE), so it does not establish absolute bioavailability of isolated schisandrin. |

## Table S11 Other metabolites

| Material and reference | Natural source and reported system | Reported observations | Design and quantitative information | Verification and limitation |
|---|---|---|---|---|
| Myristicin (Ismail and Mohamed, 2022) | Natural allylbenzene reported in nutmeg/mace and other spices; experimental myristicin from Sigma-Aldrich, purity UV. Male Sprague-Dawley rats (180–220 g); acetic acid (AA)-induced UC. | Myristicin 150 mg/kg reduced DAI; ↓ GRP78, CHOP, NF-κB mRNA; ↓ DNA fragmentation, MDA, MPO, TNF-α, IL-1β, COX-2; ↑ IL-10, colonic Nrf2 and HO-1, blood SOD and GPx. Docking showed binding to GRP78, Nrf2, and NF-κB. Effects comparable to mesalazine. No obvious abnormalities at 150 mg/kg. | Rats: 6 groups (n=10): normal, UC control, mesalazine pre/post, mesalazine post, myristicin pre/post, myristicin post; AA 3% intrarectal single dose; myristicin or mesalazine 150 mg/kg/day orally for 2 weeks before and/or 2 weeks after induction. | Single dose in acetic-acid injury; no causal target test. Docking alone does not demonstrate binding. |
| Cinnamaldehyde (Qu et al., 2021) | Major bioactive compound from cinnamon (*Cinnamomum osmophloeum* Kaneh.) essential oil; source: Shanghai Standard Technology, purity UV. Male BALB/c mice and S1P2−/− mice (C57BL/6 background); DSS-induced chronic colitis; naive CD4+ T cells. | CA 10 mg/kg ↓ body weight loss, DAI, histological damage; ↓ TNF-α, IL-1β, IL-6 mRNA; ↓ leukotriene A4 and arachidonic acid; ↓ Th17 cells in spleen and MLN, ↓ colonic CD4+IL-17A+ cells; ↓ IL-17A mRNA; ↓ S1P2, p-STAT3, p-RAC1, CDC42, RhoA, RhoB, RhoC. In vitro, CA (20 μM) ↓ Th17 differentiation, ↓ S1P2, ↓ Rho-GTPase; JTE-013 (S1P2 antagonist) blocked CA effect; S1P2 deficiency blocked CA-mediated Th17 inhibition; CA modulated lncRNA H19 and MIAT. | Mice: 3% DSS with or without CA 10 mg/kg for 7 or 14 days; S1P2−/− vs WT; endpoints: body weight, DAI, colon length, H&E, flow cytometry (Th17, Tfh, Treg), immunofluorescence, RT-qPCR, Western blot, LC-MS metabolomics. In vitro: naive CD4+ T cells under Th17-polarizing conditions ± CA 20 μM ± JTE-013 for 4 days. Statistics: t-test or one-way ANOVA; Mean ± SEM; P<0.05. | Global S1P2 knockout is not cell-specific; n/route details: UV. No positive drug control. |
| Sodium houttuyfonate (Cheng et al., 2023) | Derivative of *Houttuynia cordata* Thunb.; *Candida albicans*-overgrowth aggravated DSS-induced UC; female C57BL/6 mice (co-housing); non-co-housing sex UV. | SH 20 mg/kg improved pathological signs; ↓ LPS, MDA, MPO; ↓ TNF-α, IL-1β, IL-6; ↑ IL-10; ↑ ZO-1, occludin, claudin-1; modulated microbiota: ↑ Lactobacillus, Alloprevotella, Lachnospiraceae_NK4A136_group, Intestinimonas; ↓ Klebsiella, Bacteroides. Cohousing reproduced efficacy. | Mice: C. albicans SC5314 1×10^8 CFU on days 1, 3, 5, 7 + 2.5% DSS for 11 days; SH 20 mg/kg or fluconazole 2 mg/mL; co-housing and non-co-housing. Statistics: ANOVA/Tukey or Kruskal–Wallis/Dunn; Mean ± SD; n=3–10. | Candida-aggravated model; co-housing permits non-microbial transfer. 16S data describe bacteria, not fungal-community composition. |
| Ligustilide (Li et al., 2025) | Phthalide from *Angelica sinensis* (Oliv.) Diels and *Conioselinum anthriscoides* (H.Boissieu) Pimenov & Kljuykov; purity >98%. Male C57BL/6J mice (6–8 weeks, 20–22 g); DSS-induced UC; RAW264.7 and HEK293T cells. | Colitis/macrophage inflammation and barrier leakage↓; junction proteins↑. EGR1 engagement supported by TPP/CETSA/DARTS/SPR (KD 50.57 μM); EGR1–ADAM17–TNF-α responses↓. EGR1 overexpression reversed the effects. | Mice: 6 groups: control, model (3% DSS for 7 days), ligustilide 12.5/25/50 mg/kg, SASP 200 mg/kg; ligustilide for 10 days. In vitro: RAW264.7; LPS 1 μg/mL; ligustilide 25/50/100 μM. | Overexpression supports involvement; no EGR1 loss-of-function test. In vivo route/n require verification. |

| Material and reference | Natural source and reported system | Reported observations | Design and quantitative information | Verification and limitation |
|---|---|---|---|---|
| Shikimic acid<br>(Li et al., 2023) | Shikimic acid, purity 99.3%; reported source "anise", species unspecified. Male C57BL/6J mice; DSS colitis. | SA dose-dependently ↓ weight loss, DAI, colonic damage, MPO; ↓ TNF-α, IL-1β, IFN-γ; ↑ IgG; ↑ ZO-1, claudin-1, occludin; ↓ p-NF-κB P65, p-p38, p-JNK, p-ERK. Microbiota: ↑ Bacteroidetes and richness; ↓ Proteobacteria and Shigella at 50 mg/kg. | Six groups (n=6): control, DSS (3% DSS), SA 10/30/50 mg/kg, 5-ASA 40 mg/kg; gavage; endpoints: body weight, DAI, H&E, MPO, ELISA, Western blot (n=3), 16S rDNA (V3–V4). Statistics: one-way ANOVA + Tukey; Mean ± SD; $P<0.05$. | Source "anise" does not identify a species. Treatment duration and microbial causality unresolved. |
| Sulforaphane<br>(Zhou et al., 2024) | Isothiocyanate from cruciferous plants (broccoli, cabbage, radish); purity ≥98%. Male C57BL/6 mice (8 weeks, 18–20 g); DSS-induced colitis; RAW264.7 cells. | SFN (25, 50 mg/kg, oral gavage) reversed body weight loss, reduced DAI and colon weight/length ratio, improved histology; ↓ IL-6, IFN-γ, TNF-α, MPO, iNOS, p-NF-κB-p65; ↑ occludin, ↓ MLCK; ↓ TUNEL+ cells; ↓ NLRP3, ASC, caspase-1, IL-1β, IL-18. In vitro, SFN (5, 20 μM) ↓ LPS/nigericin-induced ROS and NLRP3 inflammasome activation. | Gavage with sulforaphane 25/50 mg/kg/day for 7 days starting 1 day after DSS induction; SASP 500 mg/kg; normal/model controls; n=6/group. DSS 4% in Methods versus 2.5% in the diagram. RAW264.7: 5/20 μM, 12 h before LPS/nigericin; WB n=3, ELISA n=6. Mean±SD; endpoint-specific efficacy P values UV. | Sulforaphane identity confirmed. DSS concentration differs between Methods and diagram; cellular LPS units also need clarification. No in vivo necessity test. |
| Panaxynol<br>(Bullard et al., 2024) | Polyacetylene (falcarinol) from American ginseng (*Panax quinquefolius* L.); Biopurify BP1656, UV purity. Female C57BL/6 mice (14 weeks); chronic and acute DSS colitis. | Improved DAI and endoscopic scores in both models; reduced crypt distortion, crypt widening, intercellular edema, goblet cell loss, and mucus loss. Microbiota: ↓ colitis-enriched Enterococcus, Eubacterium, Ruminococcus; ↑ Akkermansia, Adlercreutzia, Clostridium. Immune: ↓ macrophages (CD11b+CD68+) in colonic lamina propria; ↑ TCRβ+FoxP3+ regulatory T cells. | Chronic colitis: three cycles of DSS (1%, 1.5%, 1%) with 7 days of water between; panaxynol 2.5 mg/kg oral 3×/week from day 1; n=6 control, 19 DSS+Veh, 19 DSS+Pax. Acute colitis: 2% DSS 7 days + water 7 days; same dosing; n=10 control, 16 DSS+Veh, 16 DSS+Pax. | Preventive design; no positive drug. Mechanisms studied only in acute model; microbial/immune mediation untested. |

## Table S12 Extracts

| Material and reference | Natural source and reported system | Reported observations | Design and quantitative information | Verification and limitation |
|---|---|---|---|---|
| Sea buckthorn berry extract (Song et al., 2025) | *Hippophae rhamnoides* L. fruit, a plant used as medicine and food; 95% ethanol reflux extract; components profiled by UPLC-Q-TOF/MS. Male C57BL/6 mice; DSS-induced UC; antibiotic-treated mice; co-housing; *Faecalibaculum rodentium* colonization. | Colitis/barrier injury↓; F. rodentium/butyrate↑. Antibiotics attenuated benefit; live, but not heat-killed, F. rodentium restored protection. GPR109A antagonist blocked benefit; co-housing transferred responses. | Mice: control, vehicle/DSS (2.5% DSS 7 days + 3 days water), SBE 50/100/200 mg/kg, 5-ASA 50 mg/kg; oral gavage 10 days. Pseudo-germ-free: broad-spectrum antibiotics. Co-housing: SBE-treated or vehicle donors 1:1 with antibiotic-treated recipients + DSS. F. rodentium: vancomycin 50 mg/L; colonization $1\times10^8$ CFU daily for 10 days; MPN 5 mg/kg i.p. daily for 10 days. | Antibiotic-treated mice are not germ-free. Active extract constituents remain unidentified; colonization evidence does not establish human relevance. |
| *Glycyrrhiza uralensis* Fisch. ex DC. aqueous extract (Shi et al., 2022) | Roots and rhizomes of *Glycyrrhiza uralensis*; water extract by cold percolation; quality markers: liquiritin and glycyrrhizic acid. Male BALB/c mice (6–8 weeks, 20 ± 2 g); DSS-induced UC. | LWE improved body weight, colon length, histological damage, and mucosal barrier: ↑ occludin, claudin-1, ZO-1; ↓ IL-1β, IL-8, IL-18, TNF-α; ↑ IL-10; ↓ Th17/Treg ratio, ↓ ROR-γt, ↑ Foxp3; ↓ TLR4, MyD88, NF-κB p65. Microbiota: ↓ Bacteroidetes, ↑ Verrucomicrobiota and Akkermansia; ↑ A. muciniphila, norank_f_Muribaculaceae, Candidatus Saccharimonas, Enterorhabdus. | Mice: 6 groups (n=10): control, model (3% DSS), mesalazine 200 mg/kg/day, LWE 2/4/8 g/kg/day; gavage for 7 days after modeling. | Extract composition complex; no causal microbial or immune-pathway test. |
| *Eucommia ulmoides* Oliv. leaf aqueous extract (Lee et al., 2024) | *Eucommia ulmoides* leaf water extract, phenolics profiled by UPLC-Q-TOF/MS. Male C57BL/6 mice; DSS colitis. | Colitis/barrier injury and inflammatory/oxidative readouts↓; mucus/junction proteins and acetate/butyrate↑. Cognitive/brain endpoints improved but do not establish a direct gut–brain mechanism. | Mice: 5 groups (n=10): Con, NS (AEEL 400 mg/kg), DSS (2% DSS 5 days), DSS + AEEL 200 mg/kg, DSS + AEEL 400 mg/kg; AEEL orally for 3 weeks. | No positive drug; constituent contributions undefined. Cognitive changes do not prove a direct gut–brain mechanism. |
| *Wolfiporia cocos* (F.A.Wolf) Ryvarden & Gilb. extract (Song et al., 2024) | Fungal sclerotium of *Wolfiporia cocos*; mixed ethanol and water extract (PCEE + PCWE); polysaccharide content ~80%; 52 compounds identified by UPLC-Q-Exactive-MS. Male BALB/c mice (6–8 weeks, 18–22 g); DSS-induced acute UC. | PCE improved body weight loss, DAI, perianal prolapse/hematoma, spleen coefficient, colon length, and histological damage; ↑ mucus secretion and goblet cells. ↓ serum and colonic IL-1β, TNF-α, IL-6 (protein and mRNA); ↓ p-IκBα/IκBα and NF-κB p65; ↑ ZO-1 and claudin-1. Microbiota: ↑ diversity (Shannon, Simpson), ↑ Firmicutes, ↓ Proteobacteria and Pseudomonas; ↑ Oscillospira, Prevotella, Ruminococcus, unidentified Lachnospiraceae. | Mice: 6 groups (n=10): control, DSS (3% DSS for 7 days), SASP 200 mg/kg, PCE-L/M/H (20.46/40.92/81.84 mg/kg/day PCEE + 35.5/71.0/142.0 mg/kg/day PCWE); oral gavage for 7 days. Statistics: one-way ANOVA + Tukey; Mean ± SD; $P<0.05$. | Mixed aqueous/ethanol material; composition basis requires verification. Microbial/pathway mediation remains associative. |

| Material and reference | Natural source and reported system | Reported observations | Design and quantitative information | Verification and limitation |
|---|---|---|---|---|
| *Codonopsis pilosula* (Franch.) Nannf. aqueous extract (Li et al., 2024) | Roots of *Codonopsis pilosula*; aqueous extract (1 g/mL); source: Gansu Kailen Pharmaceutical. Male SD rats (180–220 g); TNBS/ethanol-induced UC. | Colitis/inflammation/oxidative injury↓. Metabolomic and transcriptomic pathways altered; PI3K/AKT/JAK2-associated protein readouts↓. Network-predicted glycitein targets were not individually validated. | 72 male SD rats; groups (n=12): control, model, SASP 0.3 g/kg, DS 18/9/4.5 g/kg; TNBS/ethanol; gavage once daily for 7 days. | Multicomponent preparation in TNBS injury; omics/network associations do not establish target necessity or glycitein efficacy. |
| *Astragalus mongholicus* Bunge extract (Shen et al., 2024) | Root extract of *Astragalus mongholicus*; source/purity UV. C57BL/6 mice (sex UV); DSS-induced UC; CP-M030 and NCM460 colonic epithelial cells. | Colitis/cytokines and NLRP3/caspase-1/GSDMD-N readouts↓; PLCB2↑ in colon and epithelial cells. Cell viability improved; PLCB2 necessity remains untested. | Mice: 6 groups (n=12/group; 6 for histology, 6 for homogenization): control, DSS (3% DSS for 5 days, then water for 2 days), AM 250/350/450 mg/kg, SASP 250 mg/kg; oral gavage days 1–7. Cells: 3% DSS 24 h; AM 200 μg/mL; qRT-PCR, CCK-8, ELISA. Statistics: one-way/two-way ANOVA + Tukey. | Mouse sex and extract characterization are incomplete. PLCB2 necessity not tested. |
| *Portulaca oleracea* L. leaf ethanolic extract (Alfwuaires et al., 2021) | *Portulaca oleracea* leaf 70% ethanol Soxhlet extract; HPLC/GC-MS profiling. Male albino mice; acetic acid colitis. | POE 200 mg/kg modulated body weight and colon length, reduced colon weight/length ratio; ↓ IL-1, IL-6, IL-17, TNF-α, IFN-γ, NF-κB; ↓ MPO and fecal calprotectin; ↓ total colonic bacterial load; improved colonic histology. | 24 mice, 4 groups (n=6): control, AA, POE alone, AA + POE. AA 3% (2 mL) intrarectal; POE 200 mg/kg in 0.5 mL PBS orally for 7 days from AA induction. Statistics: ANOVA/LSD or Kruskal–Wallis. | Single dose, no positive drug. Total bacterial load does not establish community-level changes or mediation. |
| *Wurfbainia villosa* (Lour.) Škorničk. & A.D.Poulsen aqueous extract (Choi et al., 2025) | Aqueous extract from fruits of *Wurfbainia villosa* (Zingiberaceae); HPLC marker vanillic acid 1.09 ± 0.01 mg/g. Male C57BL/6J mice (8 weeks, 20 ± 2 g); DSS-induced colitis. | AVE dose-dependently improved body weight and colon length; ↓ MPO; ↓ serum and colonic IL-6, IL-1β; ↓ TNF-α mRNA/protein and COX-2; ↑ ZO-1 and occludin mRNA/protein; ↓ p-ERK and p-p38. | Six groups (n=8): control, DSS (2.5% DSS days 15–20), AVE 100/200/500 mg/kg, 5-ASA 75 mg/kg; oral gavage once daily for 20 days. | Vanillic acid is a quality marker, not a proven active mediator. No causal pathway test. |
| *Piper wallichii* (Miq.) Hand.-Mazz. ethanol extract (Zhao et al., 2023) | *Piper wallichii* stem 70% ethanol extract; 14 constituents profiled. Male BALB/c DSS mice; RAW264.7 cells. | Colitis, apoptosis, and inflammatory cytokines↓; occludin/Ki67↑; Th1/Th17↓, Th2↑. TLR4/NF-κB-associated readouts↓; cellular TNF-α/IL-6↓. | Mice: normal (n=6), DSS (4% DSS for 12 days; n=7), tofacitinib 30 mg/kg (n=7), EEPW 25/50/100 mg/kg (n=7); oral daily. Cells: LPS 2 μg/mL; EEPW 10–80 μg/mL; ELISA. Histology n=4; flow cytometry n=3; Western blot n=3. | Active constituents not individually tested; pathway/microbial necessity unresolved. |
| *Prunus mume* (Siebold) Siebold & Zucc. ethanol extract (Zhang et al., 2023) | Ethanol extract of *Prunus mume* fruit (plant used as medicine and food); main components: citric acid 131.93 mg/g, malic acid 2.39 mg/g, chlorogenic acid 0.37 mg/g. Female C57BL/6 mice; DSS-induced colitis. | Colitis↓; epithelial healing/occludin/EGF↑. Colonic LPC/PLA2↑ and LPCAT3↓; cellular migration improved without increased proliferation. cPLA2 inhibitor attenuated cellular effects. | Mice: normal, DSS (2.5% DSS 7 days + water 3 days), MF 200/400/800 mg/kg, 5-ASA 100 mg/kg; n=6–10/group. Cells: NCM-460 and HT-29; MF 10/30/100 μg/mL; LPC 0.3/1/3 μM; AACOCF3 10 μM, VPLB 3 μM, BEL 0.3 μM; wound healing, Transwell, EdU. | Multicomponent extract; inhibitor findings were cellular. No in vivo cPLA2 necessity test. |

| Material and reference | Natural source and reported system | Reported observations | Design and quantitative information | Verification and limitation |
|---|---|---|---|---|
| *Morus macroura* Miq. Fruit extract (Salama et al., 2022) | 70% ethanolic extract of *Morus macroura* ripe fruits; HPLC-ESI-MS/MS identified phenolic acids, organic acids, flavonoids, 2-arylbenzofurans, and fatty acids. Male Wistar rats (200 ± 20 g); acetic acid (AA)-induced UC. | MFE 300 mg/kg reduced DAI, macroscopic score, ulcer area, and ulcer index; improved histopathology; ↓ MDA and total nitrate/nitrite; ↑ GSH and SOD; ↑ miRNA-223; ↓ TNFR1, p-NFκB p65, NLRP3, cleaved caspase-1, TNFα, IL-1β, and IL-18. Effects were comparable to sulfasalazine. | Rats: control, MFE 300 alone, AA, AA + MFE 100/200/300 mg/kg, AA + sulfasalazine 100 mg/kg; MFE oral for 7 days before AA (2 mL 4% intrarectal, day 8); n=8/group. | Preventive acetic-acid model; no miRNA-223 manipulation. Comparative responses do not establish clinical equivalence. |
| *Polygonatum odoratum* (Mill.) Druce methanol extract (Ye et al., 2022) | *Polygonatum odoratum* methanol extract; yield 20%, 55 constituents profiled. Male BALB/c mice; DSS colitis. | YZM increased colon length, improved DAI, and improved histopathology. Modulated microbiota: ↓ Campylobacterota, Desulfobacterota, Ruminococcus; ↑ Actinobacteriota, Muribaculaceae, Alloprevotella. ↓ $H_2S$ and $NH_3$; ↑ SCFAs (acetic and propionic acids). | Mice: 5 groups (n=6): control, model (3% DSS 8 days), SASP 50 mg/kg, YZM 200/400 mg/kg; intragastric. | Methanol extract; microbiota–metabolite–gas correlations lack causal intervention. |
| *Gardenia jasminoides* J.Ellis fruit extracts. (Liu et al., 2021) | *Gardenia jasminoides* fruit 70% ethanol extract; yield 21.39%, 56 constituents profiled. Male SD rats; TNBS/ethanol colitis. | GFE (50, 100 mg/kg) decreased DAI, macroscopic score, and histological damage; ↓ MPO, NO, MDA, IL-1β, TNF-α, and IL-6; ↑ SOD. | Five groups (n=7): control, TNBS, SASP 500 mg/kg, GFE 50 mg/kg, GFE 100 mg/kg; oral administration once daily for 7 days after induction; sacrifice on day 8. | TNBS injury model; constituent contributions and pathway necessity untested. |

## Table S13 Combined interventions

| Material and reference | Natural source and reported system | Reported observations | Design and quantitative information | Verification and limitation |
|---|---|---|---|---|
| Piperine and Glycyrrhizic acid (Wu et al., 2024) | Piperine from Piperaceae; glycyrrhizic acid from *Glycyrrhiza*; nanocrystals stabilized with TPGS and coated with Eudragit S100; female C57BL/6J mice; DSS-induced colitis; LPS-stimulated RAW264.7 and Caco-2 cells. | Coated nanocrystals improved colitis/barrier integrity and M2-associated polarization; mTOR↑, HIF-1α↓. Release/distribution findings support delivery potential, but drug PK is needed. | 3% DSS for 7 days; oral treatment from day 3 for 7 days; groups: control, PBS, PIP 10 mg/kg, PIP+GA, PIP/GA NCs, ES100-PIP/GA NCs (10 mg/kg PIP + 5 mg/kg GA); in vitro: LPS-induced RAW264.7 and Caco-2, 5 μg/mL; endpoints: DAI, colon length, spleen, H&E, ELISA, flow cytometry, IHC, GIT distribution, HPLC. | n UV; no standard positive drug. Coating/carrier contributions and mTOR/HIF-1α necessity unresolved. |
| Rhein and Emodin (Wang et al., 2024) | Rhein and emodin (from rhubarb) co-loaded into HA-Cys-RH/EMO nanoparticles, encapsulated in β-1,3-D-glucan yeast cell wall microparticles (YPs). Male ICR mice (22–25 g); DSS-induced UC; RAW264.7 macrophages. | Combination formulation improved colitis, cytokines, and Th17/Treg balance; PI3K/Akt phosphorylation↓; microbiota altered. Reported Q-based interaction depends on endpoint and dose; carrier contribution remains relevant. | Mice: control, DSS (3% DSS 7 days), free RH, free EMO, free RH+EMO, HA-RH/EMO NPs, HA-RH/EMO NYPs; n=6/group; RH or EMO 25 mg/kg orally from day 3 to day 10. Cells: LPS-induced RAW264.7; NPs 5 μg/mL. | No standard positive drug; carrier contribution remains relevant. Q-based interaction is endpoint-specific; mechanism lacks causal validation. |
| *Platycodon grandiflorus* (Jacq.) A.DC. polysaccharides and Hesperidin (Liu et al., 2025) | PGP: plant-derived polysaccharide from *Platycodon grandiflorus*; Hesp: plant-derived flavonoid from *Citrus × aurantium* L. Male BALB/c mice (6–8 weeks, 20 ± 2 g); DSS-induced UC; LPS-induced RAW264.7 and IEC6 cells. | Combination improved colitis and barrier/immune readouts; PI3K/AKT and IL-6/JAK2/STAT3 phosphorylation↓, PTEN↑. Greater responses than single agents do not alone establish synergy. | Mice: 6 groups (n=6): CTL, DSS (3% DSS 7 days, then water 7 days), SASP 200 mg/kg, PGP 400 mg/kg, Hesp 80 mg/kg, PGP + Hesp (400 + 80 mg/kg); oral gavage days 7–14. Cells: RAW264.7/IEC6; LPS 2 μg/mL; PGP 100 μg/mL; Hesp 25 μg/mL; PGP + Hesp (100 + 25 μg/mL); LY294002, AG490, 740 Y-P, colivelin. | Heterogeneous polysaccharide plus purified flavonoid; pharmacological mechanism tests, no genetic necessity. Greater combination response is not proof of synergy. |
| Sodium alginate and chlorogenic acid (Niu et al., 2022) | Chlorogenic acid: plant-derived polyphenol, exact source species UV. Sodium alginate: soluble dietary fiber polysaccharide from brown seaweed, species UV. LPS-stimulated RAW264.7 cells; male BALB/c mice; DSS-induced UC. | CA+SA reduced NO and IL-6 in LPS-stimulated RAW264.7 cells more than CA alone. In DSS mice, CA+SA improved body weight loss, DAI, colon length, and histology; ↓ serum IL-6, TNF-α, IL-1β; ↑ IL-10; ↓ colonic MPO; ↑ SOD; modulated microbiota (↑ Actinobacteriota, Lactobacillus, Bifidobacterium; ↓ Bacteroides, Subdoligranulum, Streptococcus). | Cells: RAW264.7; LPS 1 μg/mL; CA 6.25–400 μg/mL; SA 6.25–400 μg/mL; CA+SA. Mice: 6 groups (n=5): control, model (2.5% DSS days 1–7), mesalazine 200 mg/kg, CA 100 mg/kg, SA 150 mg/kg, CA+SA (100 + 150 mg/kg); oral gavage from day 3 for 9 days. | n=5; no dose-response or causal molecular-pathway test. Alginate properties not optimized. |
| *Astragalus mongholicus* Bunge polysaccharides and *Codonopsis pilosula* (Franch.) Nannf. polysaccharides (Tang et al., 2021) | *Astragalus mongholicus* and *Codonopsis pilosula* polysaccharide-enriched extracts. Male C57BL/6 mice; 2.5% DSS. | A3C6 most consistently improved colitis/barrier markers; cytokines↓, IL-10/IL-22/p-AhR↑. Butyrate/isovalerate and microbiota were altered; single preparations had limited effects. | 9 groups (n=8): control, model (2.5% DSS 5 days + water 2 days), 5-ASA 100 mg/kg/day, A3 (300), A6 (600), C6 (600), A3C3 (300+300), A3C6 (300+600), A6C3 (600+300); gavage days 1–7. | Heterogeneous extracts; no causal AhR or microbial/SCFA intervention. Combination effects cannot be assigned to either constituent alone. |
